# Coexpression Network Analysis in Abdominal and Gluteal Adipose Tissue Reveals Regulatory Genetic Loci for Metabolic Syndrome and Related Phenotypes

**DOI:** 10.1371/journal.pgen.1002505

**Published:** 2012-02-23

**Authors:** Josine L. Min, George Nicholson, Ingileif Halgrimsdottir, Kristian Almstrup, Andreas Petri, Amy Barrett, Mary Travers, Nigel W. Rayner, Reedik Mägi, Fredrik H. Pettersson, John Broxholme, Matt J. Neville, Quin F. Wills, Jane Cheeseman, The GIANT Consortium, The MolPAGE Consortium, Maxine Allen, Chris C. Holmes, Tim D. Spector, Jan Fleckner, Mark I. McCarthy, Fredrik Karpe, Cecilia M. Lindgren, Krina T. Zondervan

**Affiliations:** 1 The Wellcome Trust Centre for Human Genetics, University of Oxford, Oxford, United Kingdom; 2 Department of Statistics, University of Oxford, Oxford, United Kingdom; 3 Department of Molecular Genetics, Novo Nordisk A/S, Maaloev, Denmark; 4 Oxford Centre for Diabetes, Endocrinology, and Metabolism, Churchill Hospital, Oxford, United Kingdom; 5 Estonian Genome Center, University of Tartu, Tartu, Estonia; 6 NIHR Oxford Biomedical Research Centre, ORH Trust, Churchill Hospital, Oxford, United Kingdom; 7 Twin Research Unit, King's College London, London, United Kingdom; University of California Los Angeles School of Medicine, United States of America

## Abstract

Metabolic Syndrome (MetS) is highly prevalent and has considerable public health impact, but its underlying genetic factors remain elusive. To identify gene networks involved in MetS, we conducted whole-genome expression and genotype profiling on abdominal (ABD) and gluteal (GLU) adipose tissue, and whole blood (WB), from 29 MetS cases and 44 controls. Co-expression network analysis for each tissue independently identified nine, six, and zero MetS–associated modules of coexpressed genes in ABD, GLU, and WB, respectively. Of 8,992 probesets expressed in ABD or GLU, 685 (7.6%) were expressed in ABD and 51 (0.6%) in GLU only. Differential eigengene network analysis of 8,256 shared probesets detected 22 shared modules with high preservation across adipose depots (D_ABD-GLU_ = 0.89), seven of which were associated with MetS (FDR P<0.01). The strongest associated module, significantly enriched for immune response–related processes, contained 94/620 (15%) genes with inter-depot differences. In an independent cohort of 145/141 twins with ABD and WB longitudinal expression data, median variability in ABD due to familiality was greater for MetS–associated versus un-associated modules (ABD: 0.48 versus 0.18, P = 0.08; GLU: 0.54 versus 0.20, P = 7.8×10^−4^). *Cis*-eQTL analysis of probesets associated with MetS (FDR P<0.01) and/or inter-depot differences (FDR P<0.01) provided evidence for 32 eQTLs. Corresponding eSNPs were tested for association with MetS–related phenotypes in two GWAS of >100,000 individuals; rs10282458, affecting expression of *RARRES2* (encoding chemerin), was associated with body mass index (BMI) (P = 6.0×10^−4^); and rs2395185, affecting inter-depot differences of *HLA-DRB1* expression, was associated with high-density lipoprotein (P = 8.7×10^−4^) and BMI–adjusted waist-to-hip ratio (P = 2.4×10^−4^). Since many genes and their interactions influence complex traits such as MetS, integrated analysis of genotypes and coexpression networks across multiple tissues relevant to clinical traits is an efficient strategy to identify novel associations.

## Author Summary

Metabolic Syndrome (MetS) is a highly prevalent disorder with considerable public health concern, but its underlying genetic factors remain elusive. Given that most cellular components exert their functions through interactions with other cellular components, even the largest of genome-wide association (GWA) studies may often not detect their effects, nor necessarily provide insight into the complex molecular mechanisms of the disease. Rather than focusing on individual genes, the analysis of coexpression networks can be used for finding clusters (modules) of correlated expression levels across samples. In this study, we used a gene network–based approach for integrating clinical MetS, genotypic, and gene expression data from abdominal and gluteal adipose tissue and whole blood. We identified modules of genes related to MetS significantly enriched for immune response and oxidative phosphorylation pathways. We tested SNPs for association with MetS–associated expression (eSNPs), and tested prioritised eSNPs for association with MetS–related phenotypes in two large-scale GWA datasets. We identified two loci, neither of which had reached genome-wide significance levels in GWAs, associated with expression levels of *RARRES2* and *HLA-DRB1* and with MetS–related phenotypes, demonstrating that the integrated analysis of genotype and expression data from relevant multiple tissues can identify novel associations with complex traits such as MetS.

## Introduction

Genome-wide association (GWA) studies are routinely employed to identify common genetic variants contributing to complex diseases. Many replicated GWA signals have been found for metabolic traits including high-density lipoprotein (HDL), low-density lipoprotein, body mass index (BMI), triglycerides (TG) and blood pressure, greatly enhancing the understanding of the genetic basis of these traits [1]–[6]. However, statistically significant signals resulting from GWA studies do not necessarily lead directly to the identification of genes associated with disease or provide limited insights into the molecular mechanisms of the disease phenotype. In addition, the associated SNPs explain a very small proportion of the heritability estimated for the complex trait [7]. For example, a GWA study for BMI in up to ∼250,000 individuals identified 32 loci that, together, explain only 4.5% of the phenotypic variation or ∼6–11% of the genetic variation in BMI [3]. The 1000 Genomes Project Consortium tested ∼95% of common variation using low-coverage sequencing of 167 individuals and showed that only one-third of complex trait associations are likely to be caused by common coding variation, indicating that most contributions of common variation to complex traits are regulatory in nature [8].

eQTL (expression Quantitative Trait Loci) studies could help in understanding how known genetic variants identified by GWA studies influence clinical traits through gene expression, or suggest potential biological pathways. eQTLs have already been associated to several complex traits including Type I diabetes [9], asthma [10] and obesity [11]. In addition, eQTL studies have shown that top hits from GWA studies are more likely to be eQTLs [12], [13] and eSNPs are enriched for association to Type 2 diabetes (T2D) [14]. Focusing on SNPs that have been associated with an expression trait in the relevant tissue and testing whether such eSNPs are associated with disease could highlight novel genetic loci that fail to meet the stringent genome-wide significance level of GWA studies.

Metabolic syndrome (MetS) is highly prevalent, occuring in up to 22% of US individuals, and is a serious public-health problem world wide [15]. Defined by The International Diabetes Federation (IDF), it is characterized by central obesity plus the presence of two of four heritable metabolic abnormalities: raised TG; reduced HDL; hypertension; and hyperglycaemia. It is considered a serious risk factor of both T2D and cardiovascular disease [16]. Traditional approaches have highlighted insulin resistance, obesity, inflammation, and glucose and/or lipid metabolism to be important to the pathophysiology of the MetS [17], [18]. Body fat distribution plays an important role due to its association with metabolic disorders. Individuals with increased intra-abdominal/visceral fat (high waist-to-hip ratio (WHR)) are at high risk of MetS, whereas those with increased subcutaneous fat in the gluteofemoral region (low WHR) are at little or no risk of MetS [19], [20]. The adverse metabolic risk of visceral fat has been attributed to distinct metabolic properties of adipocytes in this fat depot compared with those in other sites, including differences in metabolic responses, gene expression, adipokine secretion and insulin action [21]–[23]. In addition, GWA studies have identified multiple loci that modulate body fat distribution independent of overall adiposity [24].

The estimated heritability of MetS ranges from 0.10 to 0.51, whilst that of the individual traits that constitute MetS range from 0.13 to 0.72 [25]–[27]. The clinical clustering of individual MetS traits may be explained by shared genetic and environmental factors contributing to their origin [25]. GWA studies on the individual metabolic traits have identified many genetic loci, however, a much smaller number of genetic factors that influence MetS as a clinical entity have been identified (www.genome.gov/gwastudies, accessed 02-03-2011 [28]).

It is likely that for a clinical entity such as MetS comprising multiple complex trait components, there is considerable genetic heterogeneity in causal pathways. Therefore examining many genes simultaneously using a systems-based approach, such as weighted gene co-expression network analysis [29], may be more powerful than analysing single-gene effects. A previous eQTL study in adipose tissue has led to the identification of a macrophage-enriched metabolic network which was enriched for eQTL signals and associated with obesity-related traits [11]. However, the focus of most eQTL studies so far has been on single tissue networks ignoring the fact that complex clinical entities such as MetS are the result of interactions of multiple molecular networks operating within and between tissues. In addition, it is not often recognised that molecular phenotypes such as gene expression traits are influenced by biological and technical variation, affecting power of association detection.

To uncover eQTLs in tissues relevant to MetS, we analysed gene expression profiles in abdominal (ABD) and gluteal (GLU) adipose tissue, and whole blood (WB), from 73 individuals, allowing the investigation of differential regulation between different adipose depots applying both single-gene and a network approaches. Using a second independent cohort comprising 145 and 141 twins with ABD and WB expression data collected longitudinally across two visits, we demonstrate the relative contribution of familial, environmental, and experimental variability to the MetS-associated expression phenotypes. Lastly, we identify a set of SNPs associated with the expression of genes in selected MetS-associated modules showing differential expression between the adipose depots, and test these eSNPs for association with MetS-related phenotypes in two large GWA cohorts.

## Results

### Single-gene associations between MetS and gene expression

**Figure 1:**
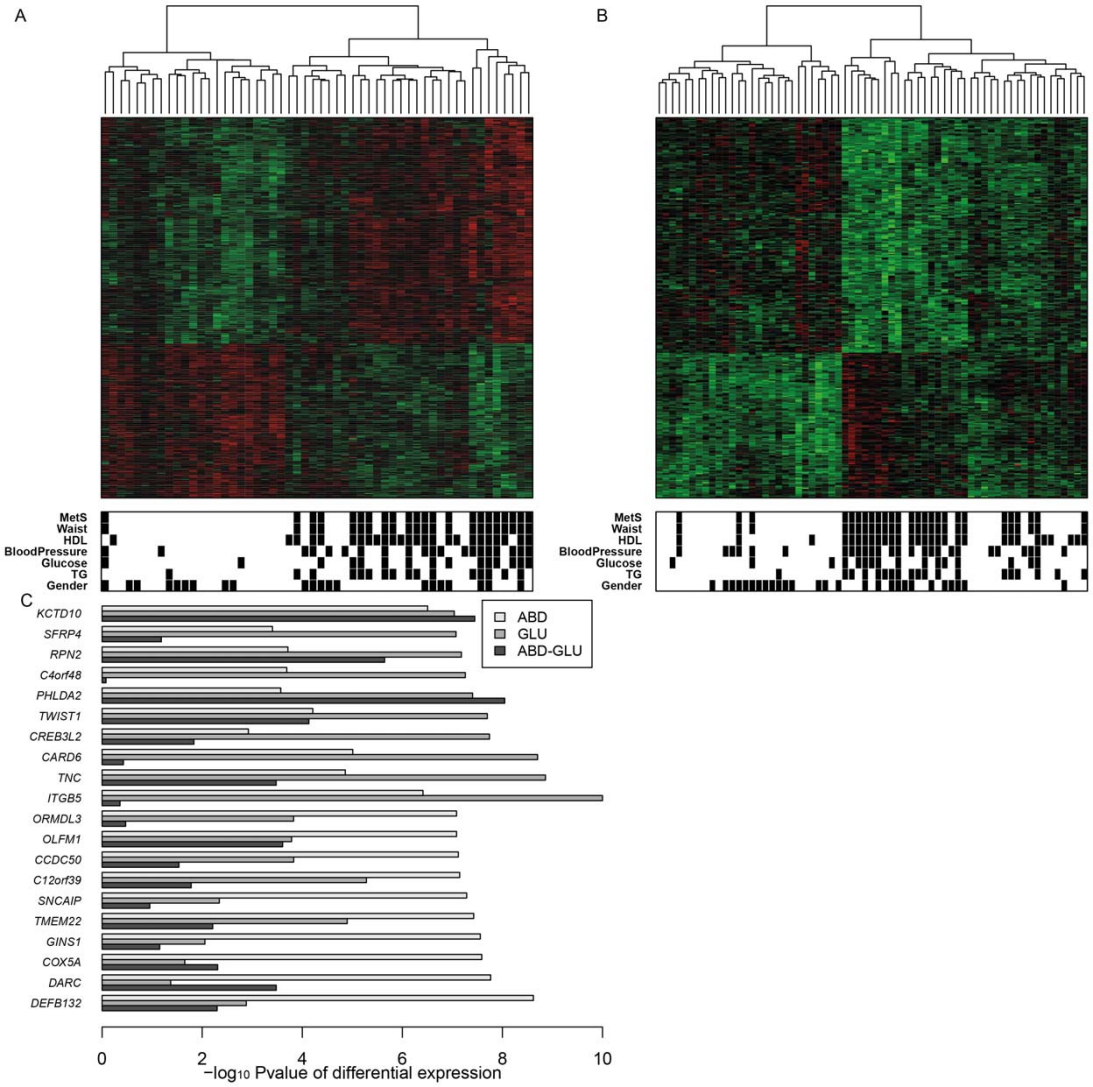
Gene expression changes related to MetS in ABD and GLU. A,B. Heatmap of scaled expression values of 893 and 335 differentially expressed genes between MetS cases and controls in ABD (A) and GLU (B) samples. The dendogram depicts hierarchical clustering of the differentially expressed genes. The bottom bars show black boxes for MetS, the presence of the MetS components (reduced HDL, raised TG, raised fasting glucose, raised blood pressure) and gender (females). C. The top 10 genes differentially expressed in ABD and the top 10 genes differentially expressed in GLU. The horizontal bars displays −log_10_ Pvalues for differential expression between MetS cases and controls in ABD and GLU and between depots.

Figure S1 shows the study design. Using Affymetrix hgu133plus2 arrays, we analysed ABD, GLU and WB samples from 29 MetS cases and 44 controls from the MolOBB study. For each subject, we collected six quantitative traits used to define MetS including waist circumference, systolic and diastolic blood pressure, TG, HDL and fasting glucose levels. We defined MetS according to IDF Criteria [16]: central obesity, as assessed by waist circumference plus any two of the following four components (raised TG, reduced HDL cholesterol, raised blood pressure, raised fasting plasma glucose) (Table 1). After filtering for high-quality array data, we limited further analyses to 54, 65, and 68 individuals and those probesets that showed a mean intensity above 4 arbitrary units of log_2_ (intensity) in at least 10% of individuals resulting in 8941 (ABD), 8307 (GLU) and 6909 (WB) gene expression profiles (probesets were mapped to Entrez Genes), respectively (see Methods). We identified 893 and 335 genes showing significant expression changes with MetS in ABD and GLU applying a 1% False Discovery Rate (FDR) correction [30] with 210 genes overlapping (‘single-gene analysis’) (Figure 1, Table S1, Table S2). Hierarchical clustering of the differentially expressed genes showed distinct clustering of the majority of MetS cases. Clustering was independent of gender or the presence of specific subsets of the two MetS components as defined in the IDF criteria (waist circumference plus any two of the four MetS components) (Figure 1).

**Table 1:** Characteristics of the participants included in ABD gene expression analyses (with both nonmissing expression and phenotype data).

Sample characteristics	All cases	All controls	Male cases	Female cases	Male controls	Female controls
Sample size (N)	22	32	13	9	20	12
Age (years)	48±5	49±5	47±4	49±6	49±5	49±5
Waist (cm)	107±14	90±11	108±13	105±16	93±10	87±12
HDL (mmol/l)	0.99±0.25	1.41±0.33	0.88±0.15	1.15±0.27	1.29±0.33	1.63±0.19
TG (mmol/l)	2.1±1.3	1.1±0.3	2.5±1.5	1.7±0.9	1.0±0.3	1.1±0.4
Diastbp (mm Hg)	90±11	77±6	89±12	91±11	76±5	79±6
Systbp (mm Hg)	134±16	120±11	132±16	136±17	120±7	122±15
Glucose (mmol/l)	5.9±0.8	5.1±0.3	5.7±0.6	6.1±1.0	5.1±0.3	5.0±0.3
Reduced HDL (N)*	20	5	12	8	5	0
Raised TG (N)	15	1	10	5	0	1
Raised Blood Pressure (N)*	14	6	7	7	3	3
Raised Glucose (N)	14	1	7	7	1	0
T2D (N)*	1	0	1	0	0	0

Values are means ± standard deviation for each quantitative variable.

*Individuals (N = 7) with treatment for lipid abnormalities or hypertension were assigned as having reduced HDL or raised blood pressure. T2D is defined as fasting blood glucose >7.0 mmol/l or antiglycemic treatment).

The large overlap between the two fat depots supports the robustness of the data and consistency across fat depots. Among the top 10 genes that were associated with MetS was *KCTD10*; SNPs at this locus were previously associated with HDL [31] (Figure 1C). To validate these findings further, we compared previously implicated MetS-associated loci [32] and obesity-related gene expression differences [11] with the MetS-associated expression differences in our study. This comparison revealed consistency for MetS-associated expression differences including *LPL*, *C3AR1*, *HSD11B1*, and *FAT3* in ABD and *APOE*, *FAT3* and *FNDC4* in GLU (Table S3, Table S4).

None of the genes was differentially expressed between MetS cases and controls in WB although for the individual MetS components (122 genes for waist circumference, nine genes for TG and one gene for HDL) significant gene expression differences were identified (not shown). Only two of these genes, *ATP5E* and *BLVRB* showed significant expression changes with multiple MetS components (waist circumference (FDR P = 9.0 *10^−3^) and TG (FDR P = 0.01); waist (FDR P = 5.7 *10^−3^) and HDL (FDR P = 4.3 *10^−3^) indicating that MetS gene expression differences were more pronounced in the fat depots, which became the main focus of further analysis.

The distribution of adipose tissue between ABD and GLU depots varies between individuals and this variation is associated with MetS and some of the WHR-associated loci have shown depot-specific differences in expression patterns and/or an enrichment of associations with metabolic phenotypes [24]. This led us to test the hypothesis that MetS-associated expression differences found for genes expressed in both depots might reflect depot-specific expression differences. Whereas 8.9% and 13% of the genes associated with MetS in ABD or GLU only exhibited depot-specific differential expression, 44 of the 210 overlapping MetS-associated genes (21%) including *KCTD10* and *C3AR1* showed evidence for depot-specific expression changes. These findings support the hypothesis that, at least for some genes, the associations with MetS reflect depot-specific differences.

### Construction of weighted coexpression networks

A Pearson correlation matrix (containing an estimate of each pairwise correlation between gene expression levels, irrespective of MetS status) was calculated and transformed into a matrix of connection strengths using a power function resulting in a weighted network (see Methods and [33]). Using these connection strengths, genes were clustered in distinct groups of highly connected genes (modules). For each gene in a module, we calculated the Module Membership (MM) by correlating its gene expression profile with the module eigengene (the first principal component of the gene expression profiles in each module). Genes with high MM values to the respective module are considered hubgenes (see Methods and [30]).We constructed gene networks separately for each of the three MolOBB tissue datasets and identified 20, 26 and 18 modules in ABD, GLU and WB, respectively. To distinguish between modules, each module was assigned an arbitrary color. Figure S2 depicts a hierarchically clustered connectivity matrix of the ABD dataset and the 20 identified modules.

### Biological significance of network modules

**Figure 2:**
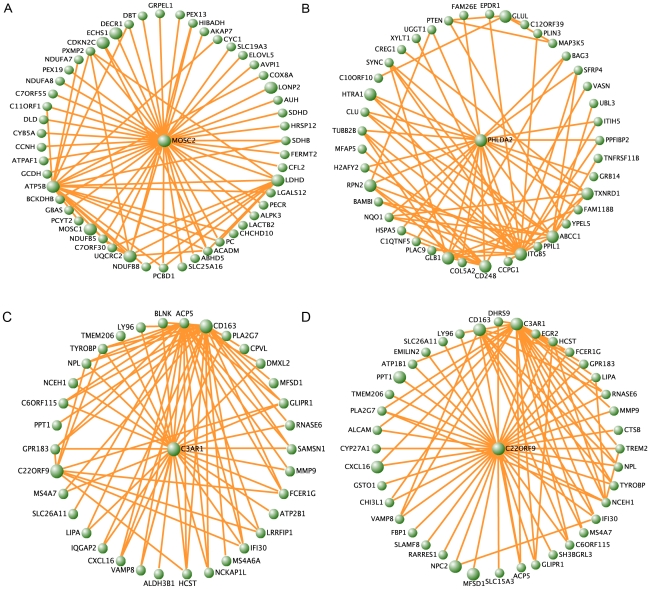
MetS-associated modules found in the different fat depots. Visualization of the ABD brown (A), GLU darkgreen (B), consensus yellow in ABD (C) and consensus yellow in GLU (D) modules, respectively. For each module the top 150 pairwise correlations (intramodular connectivities) are shown. Genes with the top 10 highest ranked module membership are displayed in larger circles.

We examined the biological significance of the identified modules by testing for 1) association with MetS 2) enrichment of Gene Ontology (GO) terms 3) hubgenes and 4) previous implicated genes. For the former, we extracted the first principal component of the gene expression profiles in each module (module ‘eigengene’) and tested its association with MetS (see Methods). From the 20, 26 and 18 modules found in ABD, GLU and WB, nine, six and zero modules, respectively, were associated with MetS (FDR P<0.01, Table 2). Four of the nine ABD MetS-associated modules and two of the six GLU MetS-associated modules showed a significant (FDR P<0.01) enrichment of GO terms (Table 3). In the ABD brown module, the eigengene was downregulated for MetS cases compared to MetS controls indicating that genes positively correlated with the eigengene (MM>0) were downregulated in MetS cases. This module was enriched for GO Biological Processes related to oxidative phosphorylation pathways (GO: 0006082 organic acid metabolic process (FDR P = 7.9*10^−7^) and GO:0006091 generation of precursor metabolites and energy (FDR P = 7.5*10^−7^). For these GO categories, the majority of the genes (more than 84% of genes with MM>0) were downregulated in MetS cases. The genes in these GO categories had a higher median MM (MM>0.74) than the 877 genes in the module (MM = 0.51) showing functional relevance of these genes in the module. Furthermore, among the top 10 genes with the highest rank of membership (hubgenes), eight were previously implicated in mitochondrial processes including generation of metabolites and energy (*ATP5B*, *ACO2*, *SUCLG1* and *UQCRC*), oxidation reduction (*MOSC1*, *MOSC2*, *LDHD*) and fatty acid oxidation (*ECHS1*) (Table S5, Figure 2A). This module contained the genes *LPL*, *FAT3* and *PPMG1* for which SNPs were previously associated with MetS (Table S4) [32].

**Table 2:** Modules for which eigengenes were significantly correlated (FDR p<0.01) with MetS in ABD (N = 9) and GLU (N = 6).

Depot	module	N genes	N DE genes*	MetS	Waist	HDL	TG	Diast bp**	Syst bp***	Glucose
ABD	brown	877	69 (8%)	2.5E-05	1.5E-06	1.0E-07	6.5E-03	5.2E-03	0.08	0.19
ABD	cyan	582	63 (11%)	4.0E-05	2.2E-09	6.1E-09	3.6E-04	4.0E-04	0.02	0.02
ABD	black	1065	142 (13%)	7.1E-05	2.2E-09	1.4E-04	6.5E-03	5.5E-05	4.2E-04	0.02
ABD	pink	331	13 (4%)	1.4E-04	9.7E-03	2.9E-03	0.43	0.02	0.13	0.19
ABD	darkred	133	22 (17%)	7.7E-04	4.0E-03	1.1E-04	4.2E-03	0.04	0.26	0.19
ABD	blue	1231	57 (5%)	9.8E-04	4.5E-06	7.2E-06	0.19	9.7E-03	0.12	0.19
ABD	darkgrey	290	63 (22%)	1.7E-03	1.2E-09	1.7E-04	3.6E-04	2.7E-04	9.4E-05	0.02
ABD	royalblue	156	9 (6%)	5.0E-03	0.28	0.01	0.71	0.50	0.96	0.90
ABD	purple	248	32 (13%)	5.5E-03	8.1E-04	0.07	0.30	1.5E-03	0.02	0.03
GLU	darkgreen	107	28 (26%)	1.4E-08	2.2E-13	3.7E-07	5.1E-06	3.9E-07	2.5E-06	2.1E-04
GLU	darkred	411	68 (17%)	2.7E-05	2.7E-08	3.7E-07	5.1E-06	2.4E-04	2.7E-04	0.03
GLU	royalblue	141	27 (19%)	2.7E-05	8.6E-09	1.7E-05	2.3e-04	2.4E-04	1.6E-04	0.04
GLU	black	338	17 (5%)	2.3E-03	9.9E-05	1.7E-04	0.02	1.7E-03	5.8E-03	0.21
GLU	brown	771	72 (9%)	2.9E-03	2.3E-06	2.1E-05	1.2E-03	5.8E-03	3.5E-03	0.23
GLU	cyan	211	59 (28%)	5.4E-03	5.1E-05	0.08	0.13	5.8E-03	0.02	0.04

FDR corrected pvalues for the associations with MetS and six quantitative metabolic traits are shown.

*DE = differentially expressed between adipose depots;

**Diast bp = diastolic blood pressure;

***Syst bp = systolic blood pressure.

**Table 3:** Biological Processes GO terms were significantly enriched (FDR P<0.01) in 15 modules associated with MetS in ABD and GLU.

Depot	Module	Term	Count	%	P value*	FE**	FDR P
ABD	brown	GO:0006091∼generation of precursor metabolites and energy	52	5.9	1.1E-09	2.5	7.5E-07
ABD	brown	GO:0006082∼organic acid metabolic process	69	7.9	2.3E-09	2.1	7.9E-07
ABD	brown	GO:0042180∼cellular ketone metabolic process	68	7.8	1.4E-08	2.0	3.1E-06
ABD	brown	GO:0022900∼electron transport chain	26	3.0	3.2E-07	3.1	5.4E-05
ABD	brown	GO:0051186∼cofactor metabolic process	31	3.5	1.7E-05	2.3	2.3E-03
ABD	cyan	GO:0009611∼response to wounding	48	8.2	3.9E-10	2.7	2.5E-07
ABD	cyan	GO:0006959∼humoral immune response	15	2.6	7.8E-08	5.8	2.5E-05
ABD	cyan	GO:0006952∼defense response	45	7.7	7.9E-08	2.4	1.7E-05
ABD	cyan	GO:0006956∼complement activation	10	1.7	1.9E-06	7.6	3.1E-04
ABD	cyan	GO:0048731∼system development	99	17.0	7.0E-05	1.4	9.0E-03
ABD	black	GO:0009889∼regulation of biosynthetic process	232	21.8	3.7E-06	1.3	2.6E-03
ABD	black	GO:0060255∼regulation of macromolecule metabolic process	257	24.1	8.5E-06	1.3	3.0E-03
ABD	black	GO:0080090∼regulation of primary metabolic process	255	23.9	1.9E-05	1.2	4.5E-03
ABD	black	GO:0051171∼regulation of nitrogen compound metabolic process	217	20.4	2.1E-05	1.3	3.7E-03
ABD	blue	GO:0002504∼antigen processing and presentation of peptide or polysaccharide antigen via MHC class II	10	0.8	6.0E-07	6.7	4.5E-04
GLU	darkred	GO:0009611∼response to wounding	41	10.0	1.2E-11	3.3	6.7E-09
GLU	darkred	GO:0006952∼defense response	38	9.3	2.6E-09	3.0	7.4E-07
GLU	darkred	GO:0009653∼anatomical structure morphogenesis	51	12.4	1.3E-06	2.0	2.5E-04
GLU	darkred	GO:0006956∼complement activation	9	2.2	2.3E-06	9.1	3.3E-04
GLU	darkred	GO:0009887∼organ morphogenesis	29	7.1	6.2E-06	2.6	7.0E-04
GLU	darkred	GO:0006959∼humoral immune response	11	2.7	7.4E-06	6.0	7.0E-04
GLU	darkred	GO:0048731∼system development	75	18.3	1.7E-05	1.6	1.4E-03
GLU	darkred	GO:0030198∼extracellular matrix organization	13	3.2	1.8E-05	4.6	1.3E-03
GLU	darkred	GO:0048513∼organ development	61	14.9	2.0E-05	1.7	1.3E-03
GLU	darkred	GO:0002252∼immune effector process	14	3.4	5.9E-05	3.8	3.4E-03
GLU	darkred	GO:0050778∼positive regulation of immune response	13	3.2	9.5E-05	3.9	4.9E-03
GLU	darkred	GO:0048583∼regulation of response to stimulus	25	6.1	1.0E-04	2.4	4.9E-03
GLU	brown	GO:0045913∼positive regulation of carbohydrate metabolic process	9	1.2	5.8E-06	7.2	3.8E-03

*P value = Fisher Exact Test;

**FE = Fold Enrichment.

The eigengene of the ABD cyan module was upregulated for MetS cases compared to MetS controls and genes in this module were enriched for immune response related GO categories. *CD163*, *C1QB*, *C1QC* and *C3AR1*, which are involved in the inflammatory response and/or complement cascade were among the strongest hubgenes (Table S5). This module contained three of seven reported genes (*C3AR1*, *HSD11B1* and *CD68*) with a highly significant MM (MM>0.75) from a previously identified macrophage-enriched metabolic network in subcutaneous adipose tissue in humans and mice that was associated with obesity-related traits and enriched for inflammatory response and macrophage activation pathway [11] (Table S3). In addition, *CD163* encodes a monocyte/macrophage specific receptor whose soluble form (sCD163) is elevated in T2D and obesity [34]. Although, gene identity and connectivity strength of the previously published module was not available, which is required for a comprehensive comparison between studies, the modules might represent similar immune-response related processes in ABD.

For the GLU samples, the brown module showed an enrichment for glucose metabolic processes (GO:0045913 positive regulation of carbohydrate metabolic process (FDR P = 3.8*10^−3^)). For the darkgreen GLU module eigengene, the strongest MetS-associated module in GLU and associated with all MetS components (FDR P<0.01), an upregulation among MetS cases was observed as compared to controls. No significant enrichment of GO terms was observed among the genes in the module (Table 3). Among the top 10 of hubgenes in the darkgreen GLU module, were *GLUL* (MM = −0.89) and *PHLDA2* (MM = 0.84) (Table S5, Figure 2B). Both these genes are highly differentially expressed between depots (FDR P<0.01). *GLUL*, encoding glutamate synthase, showed lower expression levels in GLU, as compared to ABD but the relation of this gene to MetS is unknown. *PHLDA2* encoding pleckstrin homology-like domain, family A, member 2 was upregulated in GLU as compared to ABD and is known to be involved in fetal growth with elevated placental expression associated with low birth weight [35].

### Differences in single-gene and single-tissue network approaches

**Figure 3:**
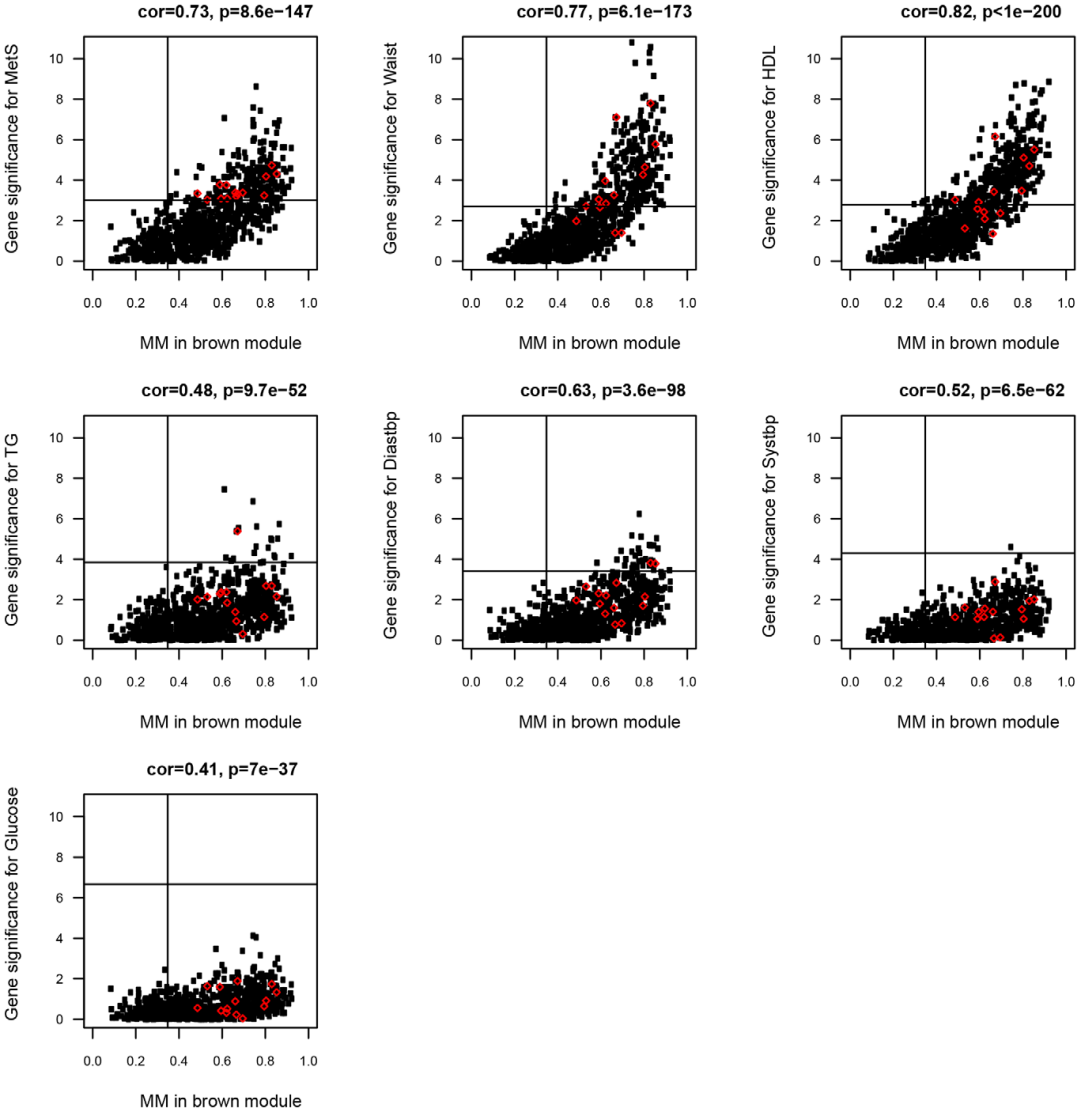
Scatterplot between MM (x-axis) and gene significance (y-axis) for MetS and the six MetS components in the ABD brown module. Gene significance was defined as −log_10_ pvalue of the probeset-clinical trait association for each gene in the brown module. Gene expression probesets marked in red showed evidence for a *cis* eQTL, and their eSNPs were examined for association with BMI, HDL and TG (Table 4).

To investigate to what extent gene expression probesets identified in the single-gene analyses as associated with MetS were included in the MetS-associated modules, and signified hubgenes, the correlation between MM and gene significance (direct association between gene expression probeset and MetS from single-gene analyses) was calculated for each gene expression probeset (see Methods and [33]). For 862/893 (97%) and 238/335 (71%) of the MetS-associated probesets in ABD (p<0.01, MM>0.36) and GLU (p<0.01, MM>0.41), a significant association with a MetS-associated module eigengene was found (147 probesets were overlapping). For the ABD brown (Pearson ρ>0.41, p<10^−36^) and the GLU darkgreen modules (Pearson ρ>0.57, p<10^−9^) most significantly associated with MetS (Table 2), the correlations between gene significance for MetS and the individual MetS components and MM were highly significant (Figure 3, Figure S3). These results imply substantial concordance in results between the two approaches and support the increased power of the network-based approach by reducing the number of tests significantly. A further advantage of the network approach is the identification of distinct functional modules within single-tissue networks that associated with MetS. Genes that fall into these modules were more highly connected than with genes in other modules (Figure S2) and their relevance can be inferred based on the correlation with the eigengene. The MetS-associated modules were enriched for immune response and oxidative phosphorylation pathways consistent with studies showing that adipose tissue secretes factors that regulate energy homeostasis and the immune response and the activation of inflammatory signalling pathways that emerges in the presence of obesity, insulin resistance and T2D [11], [36].

### Differential eigengene network and GO analysis of ABD and GLU expression data

MetS is a complex trait that is manifested in multiple tissues and where regulatory processes may act specific to a tissue as well as across tissues. The regulatory processes that play a role within a tissue may differ from those processes across tissues. It is likely that if modules of coexpressed genes are preserved among tissues, these modules may highlight communication between tissues and elucidate biological pathways that are shared among the tissues. Studying differential expression of individual genes in the module may reveal differences in pathway regulation across tissues.

To examine to what extent biological processes underlying MetS are shared and differentially regulated among the different adipose depots, we first examined the overlap of expressed genes between ABD and GLU (Figure S4). Of 8992 probesets expressed in ABD or GLU, 685 (7.6%) were expressed in ABD; 51 (0.6%) in GLU and 8256 (92%) in both adipose depots. For 679 of the 8256 probesets (8.2%), differential expression between depots was found (FDR P<0.01). GO analysis of the 685 ABD-only probesets showed enrichment for common GO terms (GO:0051171∼regulation of nitrogen compound metabolic process (FDR P = 7.5*10^−3^)) and other gene transcription terms whilst analysis of the 51 GLU-only probesets showed no enrichment. The enrichment of gene transcription categories among genes expressed in ABD only might suggest regulatory processes specific for ABD rather than genes that are able to induce gene activity changes in other tissues. The large overlap of expressed genes among ABD and GLU, however, suggest the existence of shared processes or at least communication between tissues.

To examine whether the eigengene networks are similar across fat depots, we calculated spearman correlations for the median expression (ρ = 0.98, p<1*10^−10^ and whole-network connectivities (ρ = 0.66, p<1*10^−10^) between ABD and GLU (Figure S5). These significant correlations suggest that the ABD and GLU networks are comparable.

Next, we applied differential eigengene network analysis on 8256 genes that were expressed in both the ABD and GLU datasets [29]. In this analysis, we detected 22 consensus modules, i.e., modules that are shared by the ABD and GLU datasets. To identify differences in pathway regulation between ABD and GLU depots, we examined the relationship between all pairs of the consensus module eigengenes represented by consensus networks (see Methods and [29]). For each individual eigengene within an adipose depot, we found that its relationship with the other eigengenes was highly preserved across the adipose depots, with an overall preservation network density D(Preserv^ABD,GLU^) of 0.89 (see Methods; Figure S6).

To assess the relevance of the consensus modules for MetS, we tested the consensus module eigengenes for association with MetS (Table S6). Eigengenes of seven consensus modules (six in ABD and six in GLU) were associated with MetS of which five modules were overlapping (FDR P<0.01) (Table S6), suggesting that, in general, the effect of consensus modules on MetS was not characterized by different patterns of coexpressed genes between different adipose depots. The yellow module eigengene showed the strongest association with MetS in both the ABD (FDR P = 1.4*10^−5^) and the GLU dataset (FDR P = 4.6*10^−6^) and was upregulated in MetS cases as compared to controls in both fat depots. The genes in this yellow module were enriched for immune response related processes (Table S7). Among the hubgenes of the yellow module, that is, the genes with the highest rank of module membership in both networks, were *C3AR1*, *CD163* and *c22orf9* and *NPC2* (Figure 2C and 2D and Table S8). Consistent with the overlap of hubgenes between the cyan ABD module and the consensus module, the module eigengenes of ABD cyan and the yellow consensus module were highly correlated (ρ = 0.97, p<1*10^−10^) and contained many common genes (310 genes).

The yellow module eigengenes in ABD and GLU were highly correlated (ρ = 0.81) and not differentially expressed (p = 0.64). However, 94 genes of the 620 genes (15%) were differentially expressed between depots (FDR P<0.01) which were enriched for the GO-term: GO:0009611 response to wounding (FDR P = 2.3*10^−3^) (see Methods). Among these differentially expressed genes, were the hubgenes *C3AR1*, *C1QC*, *CD163* involved in the complement cascade suggesting that the inflammatory response overlapping in the fat depots are regulated through common genes.. Thus, the results suggested the presence of a specific, highly preserved Mets-associated module enriched for immune response pathways containing a significant number of inter-depot differentially expressed genes which may indicate differential regulation between adipose depots.

### Variability of MetS-associated gene expression from single-gene analyses

Variation of gene expression traits may be driven by biological as well as experimental factors. Characterizing and quantifying sources of variation of gene expression traits or module eigengenes is important for the identification of regulatory genetic variants. In a separate dataset (MolTWIN) of 154 healthy twins, we retained gene expression of 202 ABD and 191 WB samples from 145 and 141 twins after quality control, respectively (202/191 visits, with 29/26 duplicate measurements (see Methods)). To examine whether the two independent ABD datasets were comparable, spearman correlations for the median expression (ρ = 0.96, p<1*10^−10^), whole-network connectivities (ρ = 0.51, p<1*10^−10^) and the intramodular connectivities for the brown module (ρ = 0.71, p<1*10^−10^) were calculated using the module assignments from MolOBB to calculate the connectivities (Figure S7). The significant correlations suggested that the ABD networks were comparable.

The MolTWIN dataset allowed us to decompose the biological and experimental variation underlying an expression trait into five components: familiality (genetic and common environment effects shared by twin pairs); individual environment (unique for twin individual); common visit and individual visit effects, which respectively measure the amount of shared (by twins within a pair) and non-shared variation occurring in the phenotype over the sampling period. The residual component of variation comprised experimental effects (two technical replicates of the same sample). Familiality and individual environment variation assess longitudinally stable, and common and individual visit variation, short-term biological components. Although, our main focus –given the size of the MolTWIN datasets– was on estimating familiality, we also included heritablity estimates for contrast and completeness (Table S9). We assessed the relative proportions of the five sources of variances using a twin mixed-effects modelling approach (see Methods) retaining 6787 probesets expressed in MolTWIN ABD and WB, and for four groups of probesets selected for association with MetS in MolOBB identified in single-gene analysis and expressed in MolTWIN and in MolOBB 1) 626/893 ABD probesets; 2) 205/335 GLU probesets; 3) 121/210 probesets with expression in both tissues and 4) 22/121 differentially expressed probesets (see Methods).

**Figure 4:**
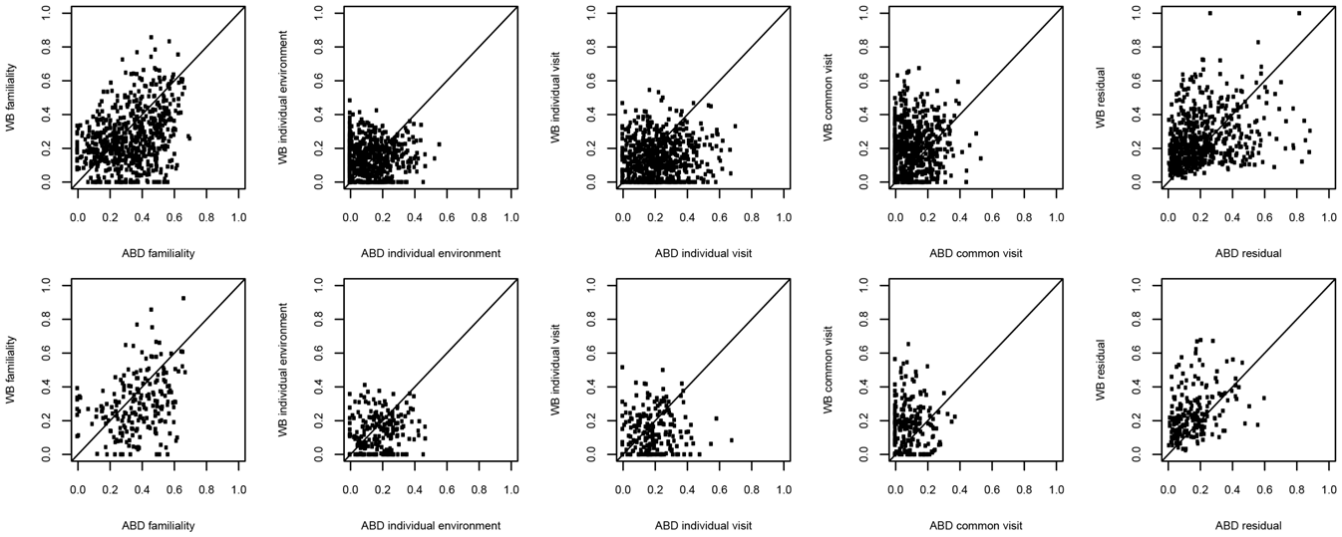
Sources of gene expression variation in different tissues. A) 626 probesets associated with MetS in MolOBB ABD B) 205 probesets associated with MetS in MolOBB GLU. Variances are decomposed in MolTWIN ABD and WB.

Familiality was the largest source of variation that contributed to the variance of the gene expression traits in ABD and WB (Figure 4, Text S2). The four groups of MetS-associated probesets showed similar familiality patterns, and their median familiality estimates were significantly higher compared to the probesets not associated with MetS, in both MolTWIN ABD and in WB (Table S9). Also, the groups of MetS-associated probesets showed a greater median familiality in ABD than in WB (Table S9). The highest median estimates were found for the 121 probesets associated with MetS in ABD+GLU (0.43, IQR: 0.18), and the 22 associated with MetS and differentially expressed between ABD and GLU (0.41, IQR: 0.15; Table S9). In addition to familiality, we estimated heritabilities for the groups of MetS-associated probesets, and the heritability patterns were similar as the familiality patterns in ABD but not in WB which might suggest an enrichment of genetic signals in ABD but not in WB.

### Variance decomposition of module eigengenes

**Figure 5:**
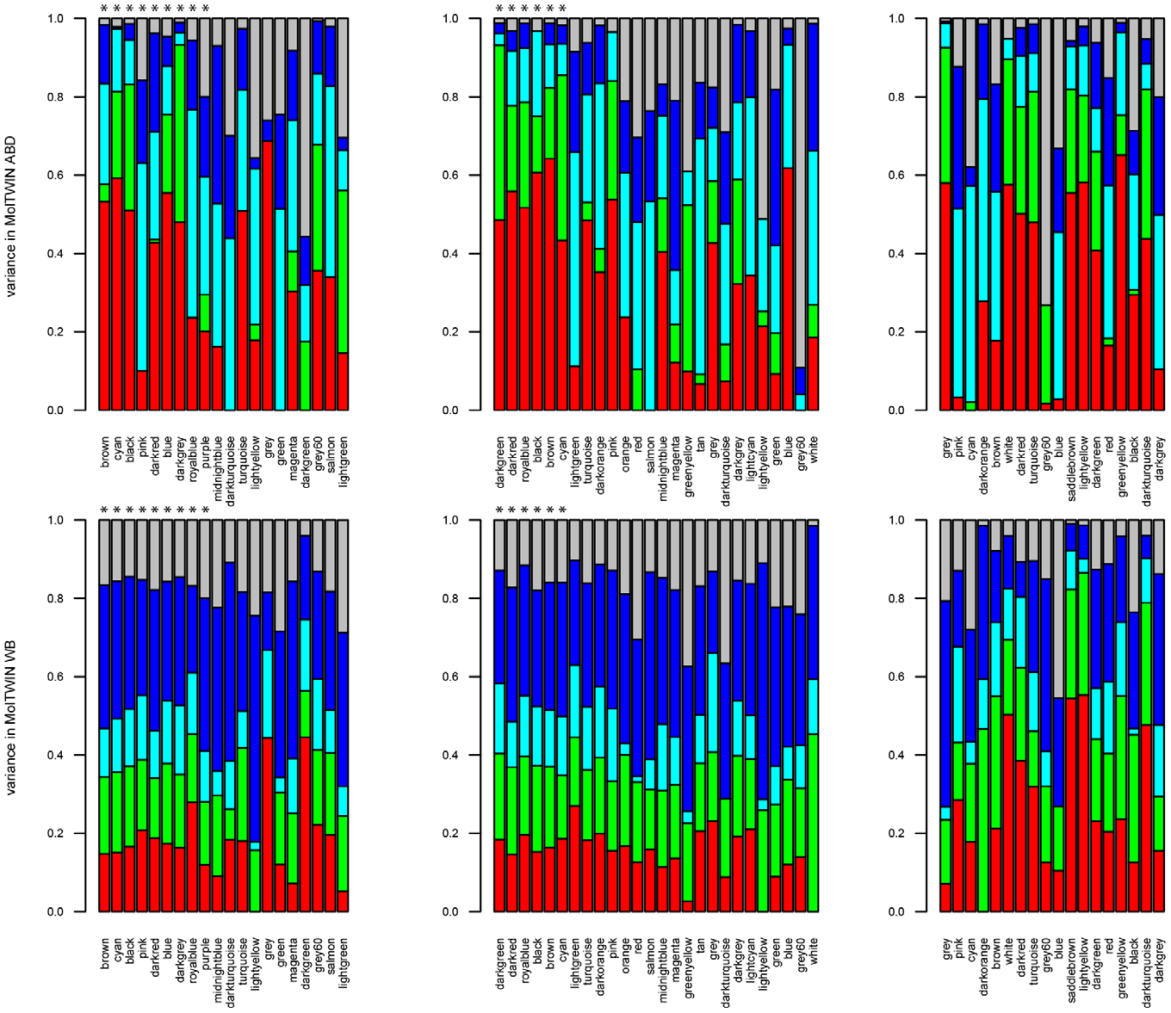
Sources of variation for module eigengenes. Median estimates from: A) MolTWIN ABD and B) MolTWIN WB. From left to right: eigengenes in MolTWIN are calculated from MolOBB ABD, GLU and WB probesets. In each plot, eigengenes are ordered by decreasing association with MetS from left to right (modules significantly associated with MetS are marked with * above the bar). Red is familiality, green is individual environment, cyan is individual visit, blue is common visit and grey displays residual variance.

We also used the MolTWIN data to characterize the sources of variation underlying the eigengenes of the MetS-associated modules from the MolOBB data. Rather than constructing networks in MolOBB and MolTWIN separately, we calculated module eigengenes in the MolTWIN study using the module assignments from MolOBB and decomposed the module eigengenes into the five variance sources as described above (Figure 5, Text S2). The variability patterns of the eigengenes were consistent with the results for probesets identified using the single-gene approach. Median familiality estimates from MolTWIN ABD (Figure 5A) were greater for MetS-associated module eigengenes than those not associated with MetS in MolOBB ABD (median = 0.48, IQR = 0.30 vs median = 0.18, IQR = 0.28, p = 0.08) and GLU (median = 0.54, IQR = 0.10 vs median 0.20, IQR = 0.28, p = 7.8*10^−4^). This pattern was not observed for familiality estimates derived from MolTWIN WB (Figure 5B). For the MetS-associated modules, median heritability estimates were significantly greater than for modules not associated with MetS in ABD (median = 0.41, IQR = 0.27 vs median = 6.9*10^−5^, IQR = 0.25, p = 0.03) and GLU (median = 0.65, IQR = 0.32 vs median = 0.14, IQR = 0.28, p = 0.007).

### eQTL Analysis of differentially expressed genes

To assess whether specific genetic loci were associated with MetS-associated gene expression in ABD and GLU, we performed *cis* eQTL analyses (*cis* defined as SNP location within 500 kb of the gene start or stop position; eQTLs are defined as genomic loci that regulate expression levels of mRNAs or proteins). For the ABD eQTL analysis, we used both ABD datasets (MolOBB and MolTWIN) comprising 189 individuals whilst for the GLU dataset we used 62 individuals from the MolOBB dataset. Out of the 8242 ABD genes tested, we found 1287 *cis* eQTL genes (FDR P<0.01) using a fixed-effects meta-analysis [37]. We found evidence for an eQTL in *cis* for 77 of the 893 probesets associated with MetS in the single-gene meta-analysis. Six of these eQTL genes showed significant inter-depot differences (Table 4). For the GLU eQTL analysis, 628 of the 8307 tested genes had an eQTL in *cis* (empirical p<0.01, see Methods). We found a *cis* eQTL for 6/335 genes associated with MetS. Two of these genes *ATP8B4* and *LTBP2*, exhibited differential expression between ABD and GLU (Table 4). Only one of these MetS eSNPs (an eSNP has been defined as a SNP associated with an expression trait), rs8207, affecting *PHOSPHO2* expression levels, was found in both ABD and GLU analyses but none of the corresponding expression probesets showed significant differences between adipose depots (Table 4).

**Table 4:** Summary of *cis* eQTL results for selected expression probes significantly associated with MetS and showing differential ABD-GLU expression in MolOBB network and single-gene association analyses (FDR P<0.01).

Depot	HGNC	Analysis approach	Module	DE-MetSP value	DE ABD-GLU P value	MMP value	SNP	eQTLP value	SNP-BMIP value*	SNP-WHR-BMIadjP value*	SNP-HDLP value**	SNP-TGP value**
ABD	*AKAP11*	single-gene: MetS & ABD_GLU DE	purple	5.3E-04	1.0E-05	7.3E-14	rs2044732	7.8E-06	0.59	0.22	4.3E-03	0.52
ABD	*HLA-DPB1*	single-gene: MetS & ABD_GLU DE	blue	9.6E-04	1.3E-04	3.6E-09	rs2064478	3.3E-08	0.38	0.80	0.48	0.43
ABD	*EFCAB8*	single-gene: MetS & ABD_GLU DE	black	2.0E-05	2.1E-04	3.3E-09	rs6579038	1.1E-05	0.89	0.27	0.33	0.38
ABD	*GALNTL1*	single-gene: MetS & ABD_GLU DE	cyan	9.1E-08	1.6E-04	5.2E-09	rs1466255	8.8E-06	0.15	0.15	0.33	0.18
ABD	*NUAK1*	single-gene: MetS & ABD_GLU DE	black	8.0E-07	1.3E-04	3.8E-04	rs10778456	2.1E-05	0.99	0.15	0.53	0.40
ABD	*ATP8B4*	single-gene: MetS & ABD_GLU DE	black	7.8E-05	3.4E-07	2.0E-09	rs10519246	3.2E-06	0.16	0.42	0.56	0.11
GLU	*ATP8B4*	single-gene: MetS & ABD_GLU DE	royalblue	3.2E-04	3.4E-07	9.3E-11	rs7495057	3.3E-05	0.33	0.68	0.77	0.66
GLU	*LTBP2*	single-gene: MetS & ABD_GLU DE	turquoise	5.3E-05	3.0E-08	4.5E-09	rs2109750	1.4E-04	0.09	0.75	0.48	0.14
ABD	*PHOSPHO2*	single-gene: MetS & eQTL overlap	blue	2.8E-04	0.11	3.4E-03	rs8207	5.9E-20	0.17	0.30	0.27	0.81
GLU	*PHOSPHO2*	single-gene: MetS & eQTL overlap	turquoise	1.5E-04	0.11	0.02	rs8207	3.1E-08	0.17	0.30	0.27	0.81
ABD	*NBN*	single tissue network	brown	9.3E-04	0.15	3.6E-05	rs2735385	6.9E-12	0.30	NA	0.98	0.79
ABD	*PGM1*	single tissue network	brown	6.6E-05	9.7E-03	2.6E-13	rs2269260	1.6E-06	0.33	NA	0.42	0.99
ABD	*RARRES2*	single tissue network	brown	1.9E-05	0.38	8.9E-15	rs10282458	1.5E-10	6.0E-04	0.28	0.47	0.52
ABD	*RPP14*	single tissue network	brown	8.2E-04	0.43	4.8E-07	rs3773010	2.3E-13	0.83	NA	0.85	0.59
ABD	*MAN2B2*	single tissue network	brown	4.7E-04	8.2E-03	2.7E-08	rs6815746	5.3E-09	0.79	NA	0.35	0.76
ABD	*SENP3*	single tissue network	brown	8.1E-04	0.31	2.1E-06	rs8073498	5.2E-06	0.40	NA	0.37	0.46
ABD	*ANAPC4*	single tissue network	brown	6.4E-04	0.86	3.9E-08	rs11937742	1.8E-05	0.02	NA	0.14	0.17
ABD	*C14orf129*	single tissue network	brown	1.8E-04	8.5E-03	5.8E-07	rs4905480	1.5E-19	0.86	NA	0.09	0.79
ABD	*SYTL2*	single tissue network	brown	1.7E-04	0.12	2.7E-06	rs580459	4.9E-05	0.77	NA	0.80	0.04
ABD	*EXD2*	single tissue network	brown	4.2E-04	0.52	5.4E-09	rs761899	2.9E-05	0.02	NA	0.47	0.83
ABD	*TOMM22*	single tissue network	brown	4.2E-04	0.86	5.4E-08	rs12004	9.5E-06	0.48	NA	0.46	0.24
ABD	*COL21A1*	single tissue network	brown	4.6E-04	0.58	2.0E-04	rs1925166	2.9E-05	0.19	NA	0.59	0.24
ABD	*OSBPL1A*	single tissue network	brown	4.9E-05	0.27	3.0E-16	rs2278664	6.0E-06	0.44	NA	0.90	0.90
ABD	*HIGD2A*	single tissue network	brown	5.7E-04	0.55	7.3E-13	rs1065212	9.7E-08	0.77	NA	0.08	0.89
ABD	*eigengene*	single tissue network	brown	NA	NA	NA	rs2835630	1.4E-07	0.15	NA	0.08	0.02
GLU	*IFNAR1*	single tissue network	darkgreen	2.1E-04	0.47	4.4E-04	rs2834188	2.8E-06	0.90	NA	0.70	0.61
GLU	*EPDR1*	single tissue network	darkgreen	1.5E-04	0.04	1.7E-11	rs1668357	6.4E-05	0.06	NA	0.56	0.38
GLU	*eigengene*	single tissue network	darkgreen	NA	NA	NA	rs909982	8.3E-08	0.58	NA	0.67	0.51
ABD	*KLHL29*	consensus-network	yellow	0.02	5.1E-08	1.2E-03	rs2577756	7.4E-07	0.55	0.79	0.53	0.60
ABD	*HLA-DPA1*	consensus-network	yellow	0.02	4.5E-05	1.1E-07	rs3077	3.1E-05	0.28	0.20	0.36	0.33
ABD	*HLA-DRB1*	consensus-network	yellow	1.4E-03	2.2E-04	1.2E-06	rs2395185	1.6E-06	0.11	2.4E-04	8.7E-04	0.40
ABD	*GALNTL1*	consensus-network	yellow	9.1E-08	1.6E-04	6.3E-09	rs1466255	8.8E-06	0.21	0.15	0.33	0.18
GLU	*HLA-DRB1*	consensus-network	yellow	0.12	2.2E-04	1.5E-05	rs660895	3.4E-05	0.23	3.5E-03	0.03	0.96
GLU	*HLA-DRB1*	consensus-network	yellow	0.12	2.2E-04	1.5E-05	rs2395185	7.3E-05	0.11	2.4E-04	8.7E-04	0.40
GLU	*LTBP2*	consensus-network	yellow	5.3E-05	3.0E-08	7.1E-09	rs2109750	1.4E-04	0.09	0.75	0.48	0.14
GLU	*PEMT*	consensus-network	yellow	0.35	2.8E-07	0.06	rs750546	3.4E-05	0.85	2.9E-03	0.24	0.05

Independent association between the *cis* eQTL SNPs and metabolic traits was assessed in two GWA datasets.

DE = differential expression;

*genomic control adjusted pvalues from GIANT consortium;

**genomic control adjusted pvalues from [4].

We similarly calculated *cis* eQTL associations for probesets in the ABD brown and GLU darkgreen modules most significantly associated with MetS in MolOBB. For 124/877 genes in the ABD brown module (877 tests), we found evidence for an eQTL in *cis*. For 14/124 genes, a high MM (p<0.01) and a significant association with MetS was found (Table 4; Figure 3). For the GLU darkgreen module comprising 107 genes, we found two eQTL genes that were significantly associated with MetS and high MM (p<0.01) (Table 4; Figure S3). For the yellow consensus module (620 genes), shared between ABD and GLU datasets, we found 69 and 26 genes with evidence for an eQTL in *cis* in ABD and GLU respectively; five eQTLs in ABD and four eQTLs in GLU, had corresponding genes exhibiting interdepot expression differences (Table 4).

To validate the eQTL analysis, we evaluated 29 SNPs and their proxies (r^2^>0.5) that were associated with MetS in a GWA of 22,161 participants [32]. In both ABD and GLU, an eQTL for *HERPUD1* (rs3764261) was found (Table S4).

Modules are groups of highly correlated genes and could be the result of transcriptional co-regulation. We examined whether we could find genomic hotspots i.e. genetic loci that regulate multiple genes that are coexpressed within the module. We tested the module eigengenes of the ABD brown, GLU darkgreen and yellow consensus modules for association with 296,017 SNPs. After multiple testing correction (FDR P<0.05), we found the 21q22.13 locus (rs2835630, p = 1.4*10^−7^, FDR P = 0.04) significantly associated with the ABD brown module eigengene and the 6p21 locus (rs909982, p = 8.3*10^−8^, FDR P = 0.02) associated with the GLU darkgreen module. The SNP at the 21q22.13 locus was within the Down Syndrome Critical Region in a high LD region containing the *TTC3* and *DSCR9* genes. Of the two genes, only *TTC3* was expressed in ABD. This gene was not differentially expressed between MetS cases and controls (p = 0.01, FDR P = 0.06) and assigned to the turquoise module (MM = −0.62, P = 3.4*10^−7^). Remarkably, TTC3 is an E3 ligase facilitating ubiquitination and degradation of phosphorylated Akt [38] whereas Akt has a key role in metabolic regulation. The SNP at the 6p21 locus was in the intronic region of *LRFN2*. This gene was however not expressed in GLU and plays a role in neuronal development. These loci may act as a master regulator of the genes in the module mediating a gene expression regulatory mechanism.

### Confirmation of associations between eSNPs and MetS–related phenotypes in two large GWA cohorts

To validate our results, we tested our prioritised eSNPs for association with MetS-related phenotypes using data from two large GWA cohorts. Based on the eQTL analyses, we prioritised a set of 32 eSNPs that were associated with MetS-associated probesets/modules (Table 4): 15 eSNPs associated with probesets in the most significant ABD (brown) module, three eSNPs in the most significant GLU (darkgreen) module, and 14 eSNPs associated with genes exhibiting inter-depot differences in the consensus (yellow) module and/or with the single-gene models (nine of which were also significantly associated with MetS). The 32 eSNPs were tested for association with individual phenotypic components of MetS and the fourteen eSNPs exhibiting ABD-GLU inter-depot differences were tested for association with WHR-adjBMI (Table 4): Association with BMI and WHR-adjBMI was assessed using data from the GIANT consortium comprising ∼120,000 individuals and with HDL and TG in >100,000 individuals from a large-scale publicly available lipid study [4]. For each of the four clinical phenotypes, a Bonferroni-adjusted significance threshold of 1.6*10^−3^ was chosen such that Pr(Number of False Positives >0) <0.05 by correcting for 32 SNP-clinical phenotype associations. This threshold was corresponding to a FDR of 0.03 across 110 tests. Adopting a much simplified scenario given the complex correlation structure of the MetS-related traits, we found three significant associations which was more than expected by chance; assuming independence between the 110 tests, the binomial probability was 7.3*10^−4^. SNP rs10282458, was significantly associated with gene expression levels of the adipokine *RARRES2* encoding chemerin, and was significantly associated with BMI (genomic control corrected p = 6.0*10^−4^). *RARRES2* gene expression levels showed a familiality of 0.53 in the MolTWIN ABD dataset and were highly connected with the brown module eigengene (MM = 0.83). In MolOBB, expression levels of *RARRES2* were strongly associated with MetS (p = 1.9*10^−5^) and with the individual components of MetS: waist (p = 1.6*10^−8^), HDL (p = 2.0*10^−5^) and diastolic blood pressure (p = 1.5*10^−4^). SNP rs2395185, which affected expression levels of *HLA-DRB1*, was significantly associated with HDL (genomic-control corrected p = 8.7*10^−4^). Expression levels of *HLA-DRB1* showed a familiality of 0.59 in MolTWIN ABD and were correlated with waist circumference (p = 2.9*10^−5^) and HDL (p = 5.3*10^−5^) in MolOBB.

Next, we tested the 14 eSNPs that were associated with ABD-GLU inter-depot differences and rs10282458 for association with WHR-adjBMI in the GIANT consortium. By focusing on WHR after adjustment for BMI, we anticipated to detect associations with body fat distribution independent of those influencing overall adiposity. SNP rs10282458 was indeed associated with BMI but not with WHR-adjBMI (Table 4). We found one significant association (genomic-control corrected) between eSNP rs2395185, influencing *HLA-DRB1* expression levels in ABD and GLU, and WHR-adjBMI (p = 2.4*10^−4^). These results may suggest that differential regulation of the *HLA-DRB1* region is associated with WHR-adjBMI.

## Discussion

Given that many molecular processes in multiple tissues could be involved in the onset of MetS, we genotyped and profiled gene expression in WB and two different adipose depots, ABD and GLU, from 73 individuals. After constructing coexpression networks for each tissue independently and between tissues, we identified MetS-associated modules of coexpressed genes enriched for immune response and oxidative phosphorylation pathways in adipose depots but not in WB. By testing eSNPs, that were associated with expression of the genes in the MetS-associated modules, for association with MetS-related phenotypes in large scale GWA datasets, we found associations with *RARRES2* and *HLA-DRB1*. Thus, by constructing networks across and within different adipose depots combined with single-gene analysis, two signals which had not reached genome-wide significance in GWA datasets of more than 100,000 individuals were identified.

Adipose tissue is a dynamic endocrine organ that secretes proteins such as cytokines and hormones, collectively named adipokines. Adipokines may regulate energy and vascular homeostasis, as well as inflammatory processes, and are involved in glucose and lipid metabolism. Chemerin, encoded by *RARRES2*, is an adipokine known to play an important role in adipogenesis and metabolic homeostasis and modulating chemotaxis and activation of dendritic cells and macrophages [39], [40]. In humans, chemerin levels are associated with multiple components of MetS including BMI, plasma TG, hypertension, and HDL [41]–[43]. In a study of Caucasian individuals, a serum chemerin concentration of 240 ug/L was selected to diagnose MetS with a sensitivity of 75% and specificity of 67% [44]. Chemerin expression and secretion from adipose tissue increases with adipocyte differentiation and obesity [39], [41]. Despite the evidence linking circulating chemerin levels with metabolic phenotypes, to our knowledge, this is the first study that identified loci near genes encoding chemerin for MetS-related phenotypes. A GWA study reported that serum chemerin levels were heritable and found a genetic association between the *EIDL3* gene and serum chemerin levels supporting a potential role for chemerin in angiogenesis [45]. Given the convergence of adipocyte and macrophage function, chemerin may provide an interesting link between chronic inflammation, often associated with obesity-related diseases, and obesity and metabolic function in human adipose tissue with MetS.

In humans, variations in adipose tissue distribution is associated to different metabolic consequences, with abdominal increase of fat producing a much greater risk for metabolic traits than gluteofemoral fat, suggesting differential regulation between the two adipose depots [20]. We identified a MetS-associated module highly preserved across the two adipose depots and enriched for immune response pathways, with 15% of the probesets differentially expressed between tissues. A modest association between eSNP rs2395185, influencing *HLA-DRB1* expression levels in ABD and GLU, and WHR-adjBMI was found. The *HLA*-associated SNP found in our study has also been linked to ulcerative colitis [46], [47]. *Cis* eQTLs of the *HLA-DRB1* locus with other SNPs have previously been associated with Type 1 diabetes in liver tissue [9] and with cholesterol levels in omental and subcutaneous fat [4] suggesting differential regulation of this locus across different tissues.

The specific identified genetic associations were found with a network approach rather than with single-gene association between expression and clinical traits, even though there was substantial concordance in results between the two strategies. Investigating coexpression networks may be a more powerful approach than a single-gene association analysis since most cellular components are connected to each other through regulatory, metabolic and protein-protein interactions and summarising coexpressed genes in a single eigengene reduces the number of tests significantly. In our study, we tested a set of SNPs, associated with expression of genes in MetS-associated expression modules in relevant tissues, with the hypothesis that these eSNPs are enriched for SNP-MetS associations. Testing this small SNP set for disease association in large GWAS cohorts revealed two SNP-disease associations, the signals of which would have been relatively weak in a genome-wide multiple-testing context.

An additional motivation for utilising a network-based approach is that it is unlikely that MetS is a consequence of an abnormality in a single gene product, but reflects the perturbations of a particular functional module in the gene network by a complex interaction of genetic and environmental interactions [48], [49]. The existence of distinct disease-specific functional modules is consistent with: findings from GWA studies observing that many genetic loci identified with GWAs for traits such as height, lipids and BMI may be not randomly distributed with respect to biological function [50], genes associated with similar disorders show higher expression profiling similarity for their transcripts, and proteins involved in the same disease have an increased tendency to interact with each other [51].

We found an enrichment of oxidative phosphorylation genes in the most significant ABD gene network module, which is consistent with previous studies showing compelling evidence for mitochondrial dysfunction in association with insulin resistance and obesity [52], [53]. Reduced mitochondrial biogenesis has been demonstrated in humans with MetS, coinciding with reduced ATP level and dysfunctional mitochondrial electron transport [54], [55]. Mitochondrial dysfunction may lead to an increased production of Reactive Oxygen Species and consequently oxidative stress which is coupled to activation of inflammatory pathways and insulin resistance in adipocytes [36], [56].

The network topology-based approach helps to uncover potential mechanisms that contribute to the shared pathophysiology of the multiple components of MetS. Defects in gene products that are part of the same pathway, may also affect other cellular functions, resulting in potential comorbidity effects. Consistent with this view, our results and those of other studies support the idea that the chronic low-grade inflammatory condition that is associated with obesity plays a role in the etiology of MetS [36]. Specifically, cells of the innate immune system, particularly macrophages, are crucially involved in adipose inflammation and systemic metabolic abnormalities [36]. *CD163*, one of the hubgenes found this study, encodes a monocyte/macrophage specific receptor whose soluble form (sCD163) is elevated in T2D and obesity [34].

It is however not clear whether obesity is the origin or whether inflammation is proximal to metabolic dysfunction [36]; a chronic excess of nutrients can trigger metabolic dysfunction and inflammatory responses simultaneously leading to metabolic excess which also leads to inflammatory responses.

In any expression study, many expression traits associated with the disease will not necessarily be causative, but instead be mostly reactive to disease. In addition, expression levels represent measurements from a heterogeneous mixtures of cells. It is important to distinguish the effect of genetic variation on gene expression from other factors that are reactive to the disease or confounding factors that also correlate with expression variability. Both our single-gene and network-based results showed that the expression probesets and modules filtered by association with MetS had increasing familiality and heritability levels, which may suggest an enrichment of genetically relevant signals.

Our results arose principally from the analysis of gene expression in ABD and GLU adipose depots, and not WB. In addition, the heritability of the genes associated with MetS in both ABD and GLU was high in ABD tissue of the twins but not in WB. Moreover, the proportion of variance that was explained by the fact that the twins attended the hospital together (common visit effect) was high in WB, indicating stronger short-term environmental effects on WB than ABD expression levels. These observations suggest that WB is not necessarily the tissue of choice to detect eQTLs that are of direct relevance to MetS.

In conclusion, we performed an eQTL study in WB, ABD and GLU and highlighted two genetic loci associated with MetS mediated by gene expression variation. Considering many genes and their interactions influence complex traits such as MetS, the integrated analysis of genotype data and expression networks across multiple tissues relevant to the clinical traits under study is an efficient strategy to identify novel genetic associations, and may offer better targets for drug development.

## Methods

### Ethics statement

The MolTWIN study was approved by St. Thomas' Hospital Research Ethics Committee (EC04/015 Twins UK). The MolOBB study received ethical approval from Oxfordshire REC C (08/H0606/107). All participants gave informed consent.

### MolOBB data collection

The MolOBB study consists of 44 healthy controls (27 men, 17 women) and 29 cases with MetS (16 men, 13 women) between 39 and 56 years old that were collected from the Oxford Biobank as part of the MolPAGE consortium [57]. Based upon the IDF Criteria (www.idf.org), MetS was assigned as central obesity (waist circumference (or BMI>30 kg/m^2^) plus any two of the following four factors: raised triglycerides, reduced HDL cholesterol, raised blood pressure or raised fasting plasma glucose [16]. Control subjects were selected to be discordant from the MetS cases (Table 1). From these individuals, ABD and GLU adipose and WB samples were taken. A total of 143 samples were obtained, with 71 subjects successfully donating both tissue types, and one individual donating only GLU. Subcutaneous adipose tissue from the abdominal wall is taken at the level of the umbilicus; subcutaneous gluteal tissue is taken from the upper outer quadrant of the buttock and WB samples were taken using EDTA and PAXgene tubes. Gene expression data is available at ArrayExpress (E-TABM-54).

### MolTWIN collection

A total of 154 twins (56 monozygotic (MZ) pairs and 21 dizygotic (DZ) pairs), were ascertained from the UK Adult Twin registry at St. Thomas' Hospital [58] and recruited to participate in this study. Gene expression data is available at ArrayExpress (E-TABM-325). Eligible volunteers were healthy, Caucasian, post-menopausal females of Northern European descent, aged between 45–76 years old. Twins were checked for zygosity using a panel of 47 SNPs [59]. Each participant donated subcutaneous adipose tissue from the abdominal wall and WB; 34 MZ twin pairs donated samples during two visits whereas 21 DZ pairs and 22 MZ pairs donated samples during one visit. Both twins in a pair visited on the same day.

### Gene expression profiling

For the WB samples, PAXgene tubes were used and RNA was extracted according to the manufacturer's protocol (PAXgene, QIAGEN). Total RNA was extracted with TRIreagent (SIGMA-ALDRICH, Gillingham, UK) from the fat biopsies and quantified using a NanoDrop. For six of the MolOBB subjects and 30 of the MolTWIN subjects from the first visit (15 MZ pairs), RNA was split into two aliquots before labelling (technical replicates). RNA was labelled using the MessageAmp II 96-well amplification kit (Applied Biosystems, CA, USA). Labelled RNA was hybridized onto Affymetrix hgu133plus2 arrays washed, stained, and scanned for fluorescence intensity according to manufacturers protocols (Affymetrix, Inc., USA). For each tissue, RNA samples were randomised and extracted in batches of 12 samples, rerandomised before labelling in 96-well plates and hybridised in batches of 12 samples on a plate-row by plate-row basis. Quality control checks involved signal intensities, background intensity, expression of control genes and spike-ins, as well as a spatial representation of the intensities. To identify outliers further, principal components analysis was performed on the normalised gene expression dataset using the NIPALS algorithm. The majority of the probes on the hgu133plus2 arrays were collected into 17,726 non-overlapping probesets according to Entrez Gene annotations provided by Dai *et al.* [60].

### Gene expression preprocessing

After outlying arrays had been removed, there remained data in the MolOBB study from 54 ABD samples (four in technical duplicate), 65 GLU samples (five in technical duplicate) and 68 WB samples. During 202 visits, 231 MolTWIN samples from ABD (29 technical replicates) were successfully profiled from 145 individuals. From the WB samples, 217 MolTWIN gene expression profiles for 141 individuals were generated during 191 visits (26 technical replicates). All arrays were normalised concurrently across datasets for comparisons between tissues and separately for comparisons within a single tissue using GC robust multi-array analysis [61]. Gene-specific expression summaries were averaged across technical replicates of a sample. We then filtered the data, retaining only those probesets that were annotated to an autosomal location, and also showed a mean intensity above 4 arbitrary units of log_2_ (intensity) in at least 10% of individuals. After this filtering stage in the MolOBB, there remained 8619 probesets in the ABD-GLU dataset, 8941 in the ABD dataset, 8307 in the GLU and 6909 probesets in the WB dataset. In the MolTWIN study, 8928 probesets in the ABD dataset and 9071 probesets in the WB dataset remained.

### Genotyping and quality control

DNA was successfully extracted from WB samples using GeneCatcher (Invitrogen Life Technologies, Carlsbad, USA) according to manufacturer's protocol. 166 samples (70 MolOBB, 96 twins (one MZ individual per MZ pair and two DZ individuals per DZ pair)) are genotyped with Illumina 317K BeadChip SNP arrays (Illumina, San Diego, USA). Genotype data (EGAS00000000102) is available at EGA.

Quality control on the genotyped subjects was performed applying slightly changed quality control filters as described previously by the Wellcome Trust Case Control Consortium [62]. Two twin samples are removed due to sample success rate <95% and three samples (two twins, one MolOBB) were removed due to non European ancestry. SNPs are removed when minor allele frequency (MAF) <1% or showed a success rate <95% and MAF>5%, and when <99% and MAF<5%. Hardy-Weinberg equilibrium was calculated by combining all unrelateds of the MolOBB and MolTWIN dataset (e.g.one twin per twinpair) and SNPs were removed if P value<10^−4^. After QC, genotypes of the ungenotyped MZ twin were copied from genotyped MZ twin. Finally, we included 69 MolOBB individuals and 144 twins genotyped for 296,017 autosomal SNPs.

### Statistical analysis

#### Identifying differentially expressed genes

For the differential expression analysis between MetS cases and controls, we investigated the effect of gender, RNA Integrity Number, age and plate on gene expression measurements. Several probesets were associated with gender and plate but none with age or RNA Integrity Number. We then tested all detected probesets for association with MetS adjusting for gender and plate-effects using linear models. To investigate whether genes are differentially expressed between ABD and GLU, we used a linear mixed model on in which tissue (ABD versus GLU), MetS case-control status, gender, and plate were fitted as fixed effects, and subject (sample donor) as a random effect. Models were fitted using the Rpackage Maanova [63]. The pvalues from the 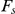 test were corrected for multiple testing using FDR [30] and probesets were considered significant if the adjusted p-value of the 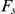 test was <0.01.

#### Construction of the weighted gene coexpression networks

We used the Rpackage WGCNA for network construction. To construct single tissue and consensus eigengene networks, we adjusted the tutorials from the WGCNA website (http://www.genetics.ucla.edu/labs/horvath/CoexpressionNetwork/Rpackages/WGCNA/Tutorials/index.html). A weighted gene co-expression network reconstruction algorithm was used to reconstruct co-expression networks for each of the three MolOBB datasets (ABD, GLU and WB) [64]. Briefly, a Pearson correlation matrix (gene-by-gene matrix) between all gene expression pairs was constructed. To define the weighted coexpression network, an adjacency matrix was constructed using a soft power adjacency function 
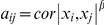
. This parameter 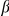 of the power function was defined in such a way that the resulting co-expression network (adjacency matrix) satifies approximate scale-free topology. To measure how well the network satisfied a scale-free topology, we used fitting index 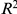 of the linear model that regressed 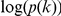 on 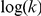 where k is connectivity and 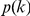 is the frequency distribution of connectivity [64]. The fitting index of a perfect scale-free network is 1. For the MolOBB ABD, GLU and WB datasets, we chose a power of 5, 6 and 12, respectively, which resulted to an approximate scale-free topology network with the scale-free fitting index 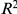 greater than 0.8. The distribution 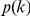 of the resulting network approximated a power law: 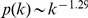 [64].

Modules were defined as groups of genes with similar patterns of connection strengths with all other genes of the network. The adjacency matrix was transformed into a topological overlap matrix. To identify modules of highly co-regulated genes, we used average linkage hierarchical clustering to group genes based on the topological overlap of their connectivity, followed by a dynamic cut-tree algorithm to cluster dendrogram branches into gene modules [65].

To summarise the gene expression profiles of an entire module by a single gene expression profile, module eigengenes were computed. Gene expression profiles for each module were decomposed using singular value decomposition 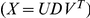, where 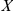 is a n-by-g matrix where g is the number of genes and n the number of samples. The first column of 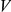 represented the module eigengene. These module eigengenes were tested for association with the MetS-related phenotypes adjusted for plate and gender using a linear regression model. These associations were adjusted for multiple testing by applying a 1% FDR [30].

To determine whether the MetS-associated modules are biologically meaningful, GO enrichment analyses were conducted using DAVID [66]. For each of the MetS-associated modules, we examined enrichment of Biological Process GO terms as compared to the expressed probesets of that tissue using a Fisher's exact test. FDR Pvalues<0.01 were considered significant [30]. Whole network connectivities were defined as the sum of connection strength with the other network genes. The intramodular connectivity 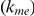 of the i^th^ gene is defined as the absolute value of the correlation between the i^th^ expression profile and the module eigengene (ME): 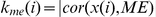. Modules were visualised using the program VisANT [67].

To assess the preservation of network properties across the ABD and GLU datasets, we applied differential eigengene network analysis to identify consensus modules [29]. These consensus modules were defined using consensus dissimilarity and used for hierarchical clustering. To compare the consensus eigengene networks of ABD and GLU whose adjacency matrices are 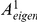 and 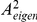 we used a preservation network 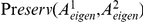 in which adjacencies are defined as 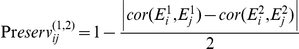. Here, 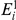 and 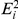 denote the eigengenes of the I^th^ consensus module in ABD and GLU, respectively. High levels of the preservation network indicate a strong preservation between I and J across the two networks. The density D is an aggregate measure of adjacency preservation between the 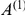 and 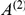 [29].

#### Variability analysis

Maximum likelihood estimation of variance components from the MolTWIN dataset was performed by fitting a linear mixed-effects model using the lme4 package [68]. For each normalised probeset or module eigengene, 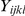, we fitted a model for the l^th^ aliquot (1,2) from a biological sample taken at the k^th^ visit (1,2) of the j^th^ twin (1,2) from the i^th^ pair (1,2,..,76):
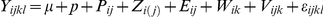
with 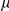 the overall mean and 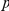 the batch-effect i.e. modelling variation across 96-well plates (fixed effects). The random effects 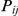, 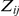, 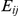, 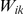, 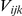, 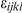 represent pair (common to a pair of twins, irrespective of zygosity), zygosity indicator (shared by a pair of MZ twins (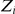) but not by a pair of DZ twins (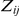)), individual environmental, common visit, individual visit and residual experimental error effects, respectively. The total phenotypic variance 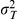 can be decomposed into the variances of 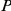, 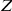, 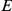, 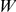, 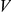 and, 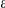 respectively: 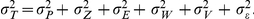


To estimate the familiality (genetic and common environmental components) among twin pairs, we calculated: 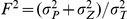. where 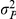 represents the genetic and common-environmental phenotypic covariance between a pair of DZ twins, irrespective of zygosity. DZ pairs share half their additive genetic variance plus all of their common environmental variance. MZ pairs share this variance, but in addition also share the remaining half of additive genetic variance. The latter extra covariance is parameterized by 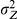. The heritability was estimated by 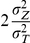. Differences between the familiality distributions in MolTWIN ABD and WB were determined using the Wilcoxon Signed Rank Test. Familiality differences between the MetS-associated probesets or module eigengene and the probesets or module eigengenes that were not associated with MetS were calculated using the Wilcoxon Rank Sum Test.

#### eQTL analysis

We selected subjects who had complete data on plate, gender, casecontrol status, gene expression and genotypes. We set out to identify SNPs that affect gene expression levels in *cis*. We defined an association between a SNP and gene expression level as *cis*-acting if the SNP was located within 500 kb from the start or stop position of the annotated gene (NCBI build 36). Genotypes were coded as 0, 1, and 2 corresponding to the counts of the minor allele and an additive model was fitted in all models. For each of the genes, each SNP in the *cis* region was tested independently.

In the MolOBB study, SNP associations were calculated by regressing expression level against using a linear regression model adjusting for gender and plate-effects. In the MolTWIN study, we fitted linear mixed models, for which twin pairing, zygosity, individual visit, common visit, individual environment and residual effect were fitted as random effects and genotype and plate as fixed effects. eQTL associations in MolOBB and MolTWIN were calculated with the Rpackages lm and lme4, respectively. In the ABD meta-analysis, we combined study-specific allelic effect-estimates on 8242 probesets expressed in both ABD MolOBB (N = 52) and MolTWIN (N = 137) using a fixed effect model, using the inverse of the variance of the study-specific allelic effect-estimates to weight the contribution of the two studies [37]. Results were checked for heterogeneity and adjusted for multiple testing by FDR [30].

#### Follow-up of SNP–MetS associations in independent cohorts

In order to followup eSNPs in independent cohort we assembled a set of 32 eSNPs based on two criteria: i. Expression levels had to be differentially expressed between MetS cases and controls and showed a high MM (p<0.01) with the brown module in ABD or the darkgreen module in GLU. ii. Expression levels in yellow consensus module or gene-by-gene model had to be differentially expressed between depots. In order to confirm the associations between the 32 eSNPs and the MetS related phenotypes in independent cohorts, we obtained results of relevant meta-analyses conducted by two consortia. For the BMI associations, 32 eSNPs were tested for association in the GIANT consortium consisting of >120,000 individuals. To confirm associations with TG and HDL, we used previously published GWA results comprising >100,000 individuals [4]. For the 14 eQTL associations affecting gene expression levels associated with interdepot differences, 14 eSNPs were examined for association with WHR-BMIadj. Meta-analysis statistics were obtained using weighted z-statistics and corrected for genomic control. For each of four clinical phenotypes, the significance threshold 1.6*10^−3^ is chosen such that Pr(Number of False Positives >0) <0.05. Exact binomial probabilities were calculated through repeated applications of the standard binomial formula.

We selected 29 variants and their proxies (r^2^>0.5) previously associated to MetS or a pair of MetS traits [32] and tested these for association with expression levels in ABD and GLU. Expression levels of genes within 500 kb around SNP were examined for differential expression between MetS cases and controls.

### URL

All code is available at http://www.well.ox.ac.uk/ggeu/PLoSGenet_Minetal_MolPAGE/.

## Supporting Information

Figure S1 Study design for the three strategies. Pink, green and purple colors represent single-gene approach, single tissue eigengene network analysis and differential eigengene network analysis, respectively.
(TIF)

Figure S2 Heatmap plot of the topological overlap in the ABD gene network of 1000 random selected genes. In the heatmap, each row and column corresponds to a gene, light color displays low topological overlap, and darker color displays higher topological overlap. The gene dendogram and module assignment are shown along the left and top. The cyan and brown modules are clearly seen by the darker squares along the diagonal.
(TIF)

Figure S3 Scatterplot between MM (x-axis) and gene significance (y-axis) for MetS and the six MetS components in the GLU darkgreen module. Gene significance (y-axis) is defined as −log_10_ pvalue of the probeset-clinical trait association for each gene in the darkgreen module. The red indicated genes show genes with evidence for a *cis* eQTL and their eSNPs are examined for association with BMI.
(TIF)

Figure S4 Venn diagram of number of expressed genes in MolOBB ABD and GLU.
(TIF)

Figure S5 Preservation of genes across fat depots. A. Scatterplot of 8256 median expression levels for ABD versus GLU. B. Scatterplot of whole-network connectivities for ABD versus GLU. Each point corresponds to a gene expression level. Spearman correlations and the corresponding p-values are displayed in the title of each plot. The whole-network connectivity is strongly preserved between ABD and GLU.
(TIF)

Figure S6 Differential eigengene network analysis across different depots. A, B Dendrograms of consensus module eigengenes in ABD and GLU. C, F.The heatmap plots of eigengene adjacencies in each eigengene network. Each row and column corresponds to one eigengene (labeled by consensus module color). Within each heatmap, red indicates high adjacency and green displays a low adjacency as shown by the color legend. D. Barplot shows the preservation of relationships of consensus eigengenes of the consensus modules. D represents the overall preservation measure. E. Adjacency heatmap for the pairwise preservation network of ABD (rows) and GLU (columns). Red indicates the adjacency.
(TIF)

Figure S7 Preservation of genes across MolOBB and MolTWIN study. A. Scatterplot of 8242 median expression levels for MolOBB ABD versus MolTWIN ABD. B. Scatterplot of whole-network connectivities for MolOBB ABD versus MolTWIN ABD. C. Scatterplot of intramodular connectivities of genes in the brown module for MolOBB ABD versus MolTWIN ABD. Each point corresponds to a gene expression level. Spearman correlations and the corresponding p-values are displayed in the title of each plot. The whole-network connectivity and intramodular connectivities of genes in the brown module are preserved between MolTWIN and MolOBB.
(TIF)

Table S1 Genes differentially expressed between MetS cases and controls in ABD (‘single gene analysis’).
(DOC)

Table S2 Genes differentially expressed between MetS cases and controls in GLU (‘single gene analysis’).
(DOC)

Table S3 Module membership and association with MetS and BMI in the MolOBB ABD dataset of genes identified by Emilsson *et al.* [11] as part of a macrophage-enriched metabolic network in subcutaneous adipose tissue and associated with obesity-related traits.
(DOC)

Table S4 Evaluation of GWA SNPs previously associated with MetS.
(DOC)

Table S5 Hubgenes (genes with highest rank of module membership) in the modules strongest associated with MetS in the ABD and GLU single-tissue networks.
(DOC)

Table S6 Modules for which eigengenes were significantly correlated (FDR p<0.01) with MetS in ABD (N = 6) and GLU (N = 6). FDR corrected pvalues for the associations with MetS and six quantitative metabolic traits are shown.
(DOC)

Table S7 Enrichment of Biological Processes GO terms in yellow consensus modules.
(DOC)

Table S8 Hubgenes (genes with highest rank of module membership) in the yellow consensus module in both the ABD and GLU networks.
(DOC)

Table S9 Median familiality estimates, assessed in MolTWIN ABD and WB, of four groups of MolOBB MetS-associated probesets identified using the single-gene approach, compared with probesets not associated with MetS.
(DOC)

Text S1 Members of GIANT consortium and members of MolPAGE consortium.
(DOC)

Text S2 Variability of MetS-associated gene expression.
(DOC)
