# Supplementary figures and images for: Coexpression Network Analysis in Abdominal and Gluteal Adipose Tissue Reveals Regulatory Genetic Loci for Metabolic Syndrome and Related Phenotypes
Source: PLoS Genet. 2012 Feb 23;8(2):e1002505. doi: 10.1371/journal.pgen.1002505 (PMC3285582; doi:10.1371/journal.pgen.1002505)

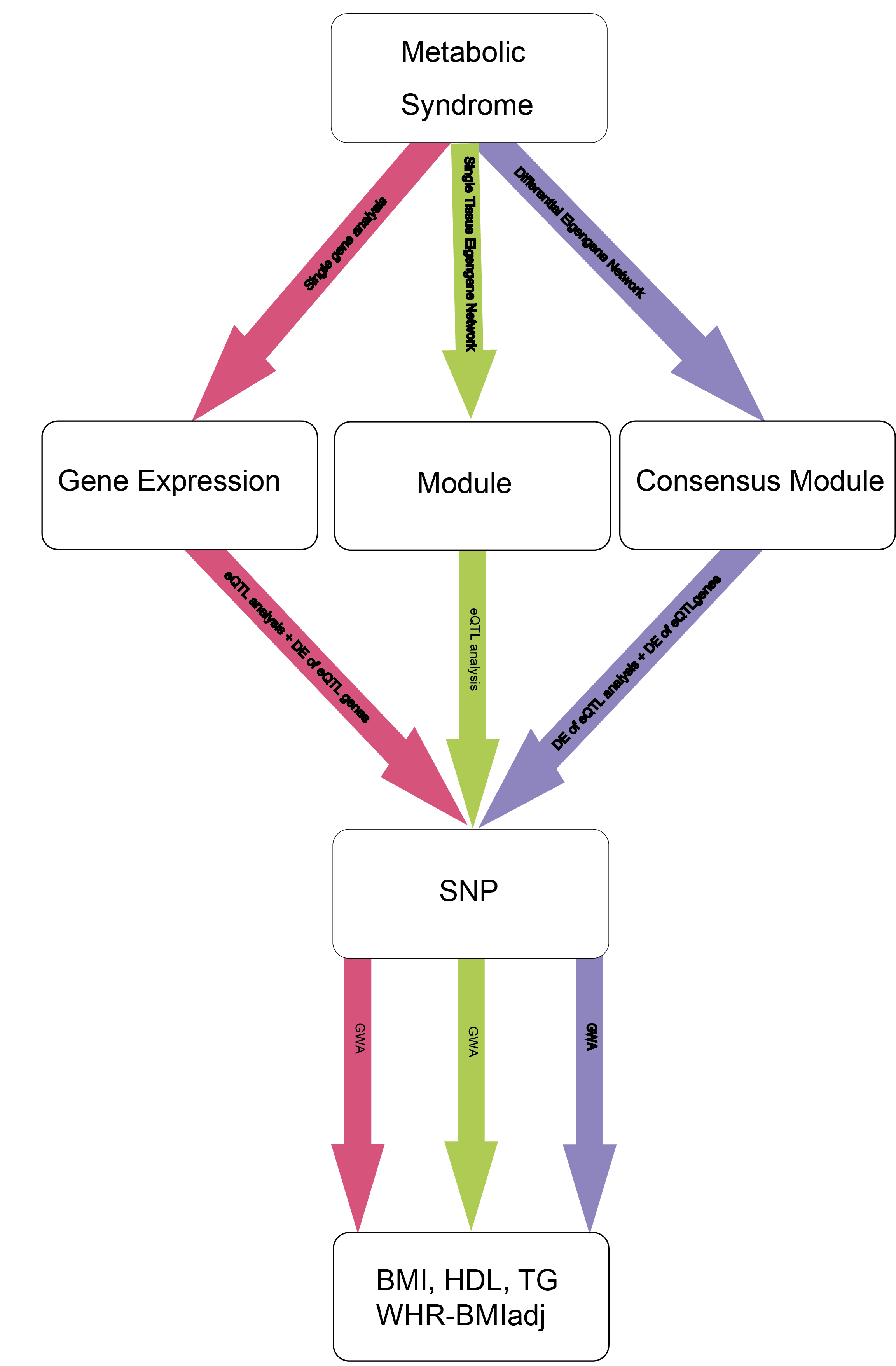

Supplement: Figure S1 — Study design for the three strategies. Pink, green and purple colors represent single-gene approach, single tissue eigengene network analysis and differential eigengene network analysis, respectively. (TIF) [file pgen.1002505.s001.tif]

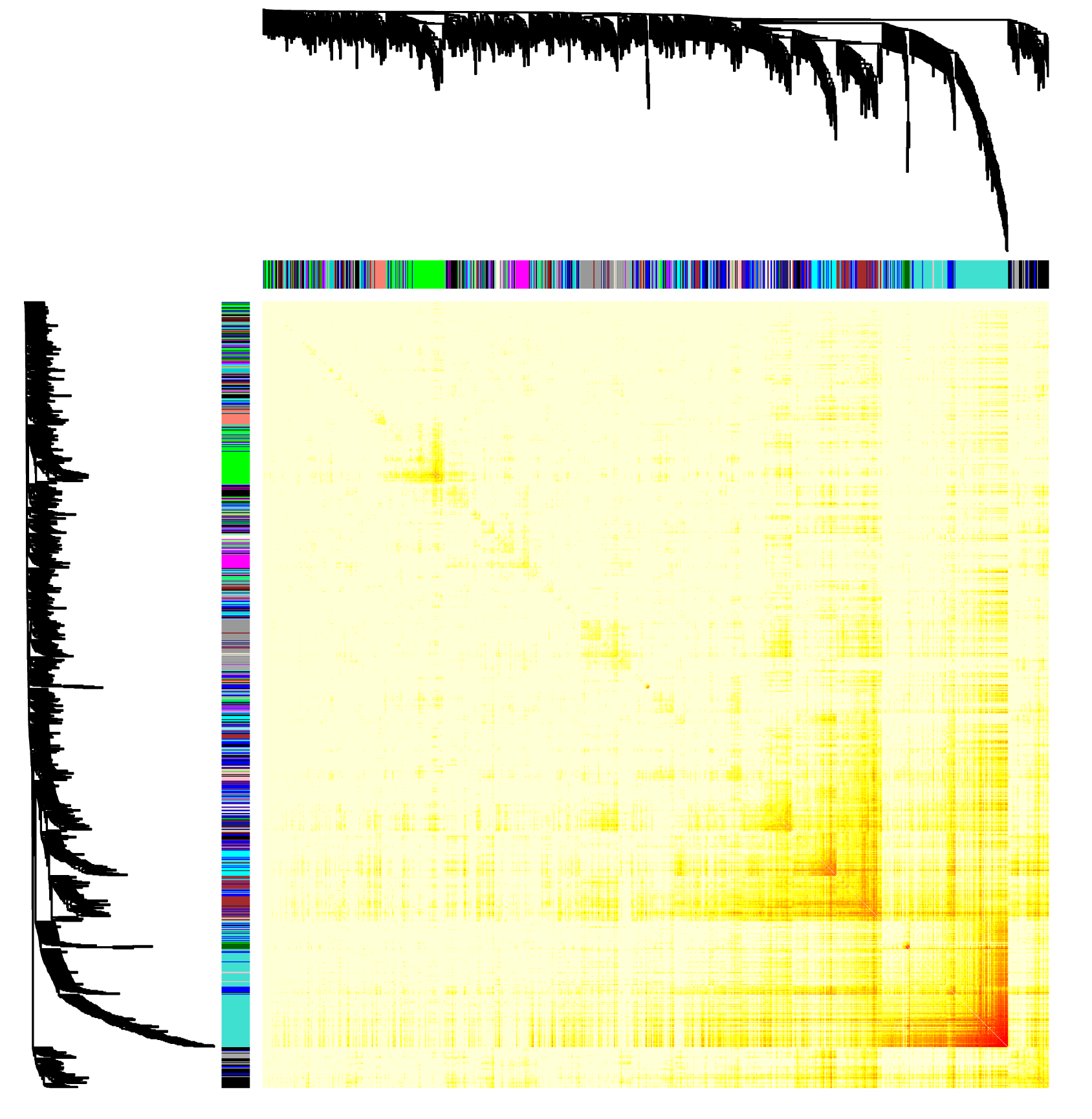

Supplement: Figure S2 — Heatmap plot of the topological overlap in the ABD gene network of 1000 random selected genes. In the heatmap, each row and column corresponds to a gene, light color displays low topological overlap, and darker color displays higher topological overlap. The gene dendogram and module assignment are shown along the left and top. The cyan and brown modules are clearly seen by the darker squares along the diagonal. (TIF) [file pgen.1002505.s002.tif]

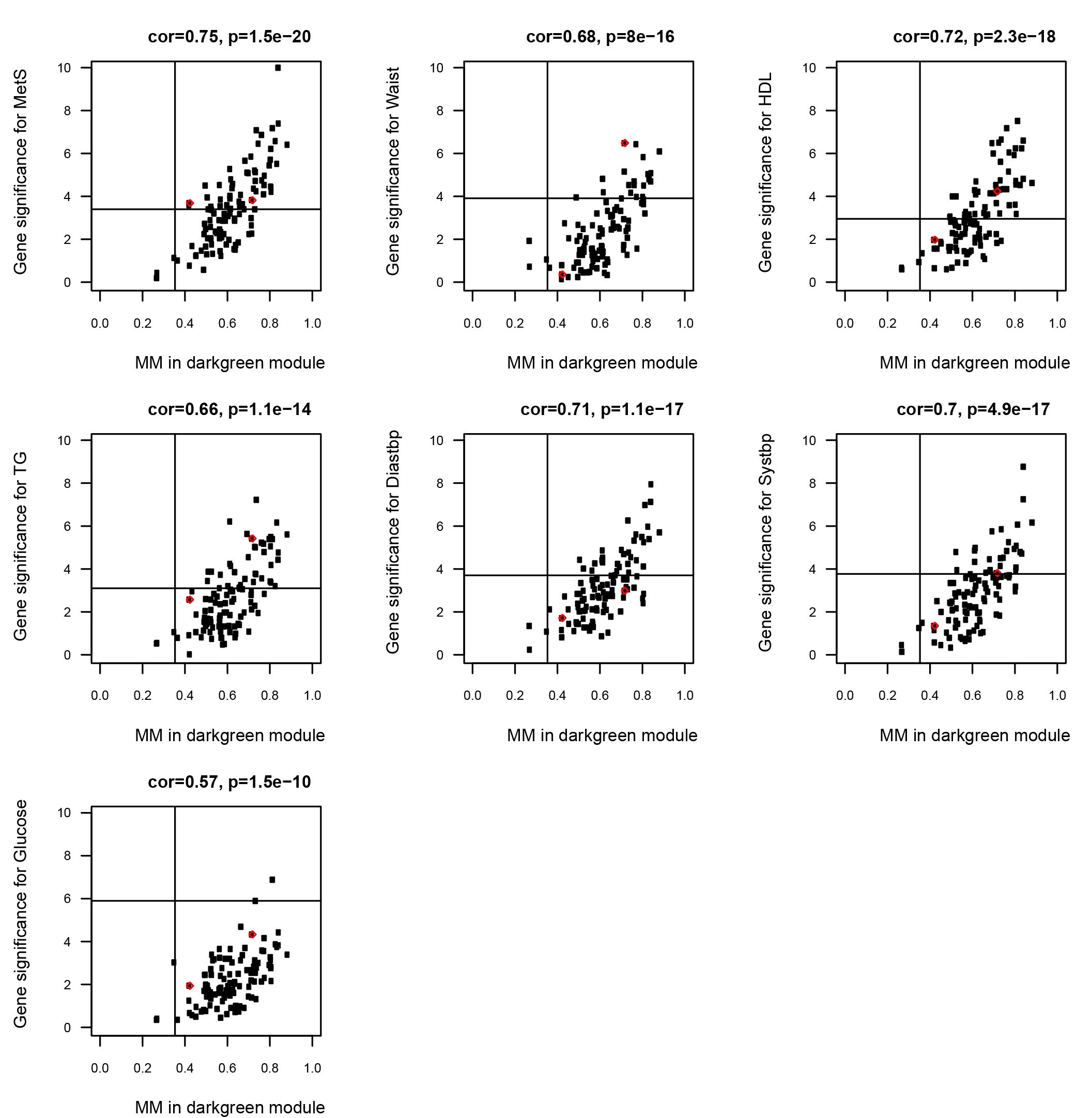

Supplement: Figure S3 — Scatterplot between MM (x-axis) and gene significance (y-axis) for MetS and the six MetS components in the GLU darkgreen module. Gene significance (y-axis) is defined as −log10 pvalue of the probeset-clinical trait association for each gene in the darkgreen module. The red indicated genes show genes with evidence for a cis eQTL and their eSNPs are examined for association with BMI. (TIF) [file pgen.1002505.s003.tif]

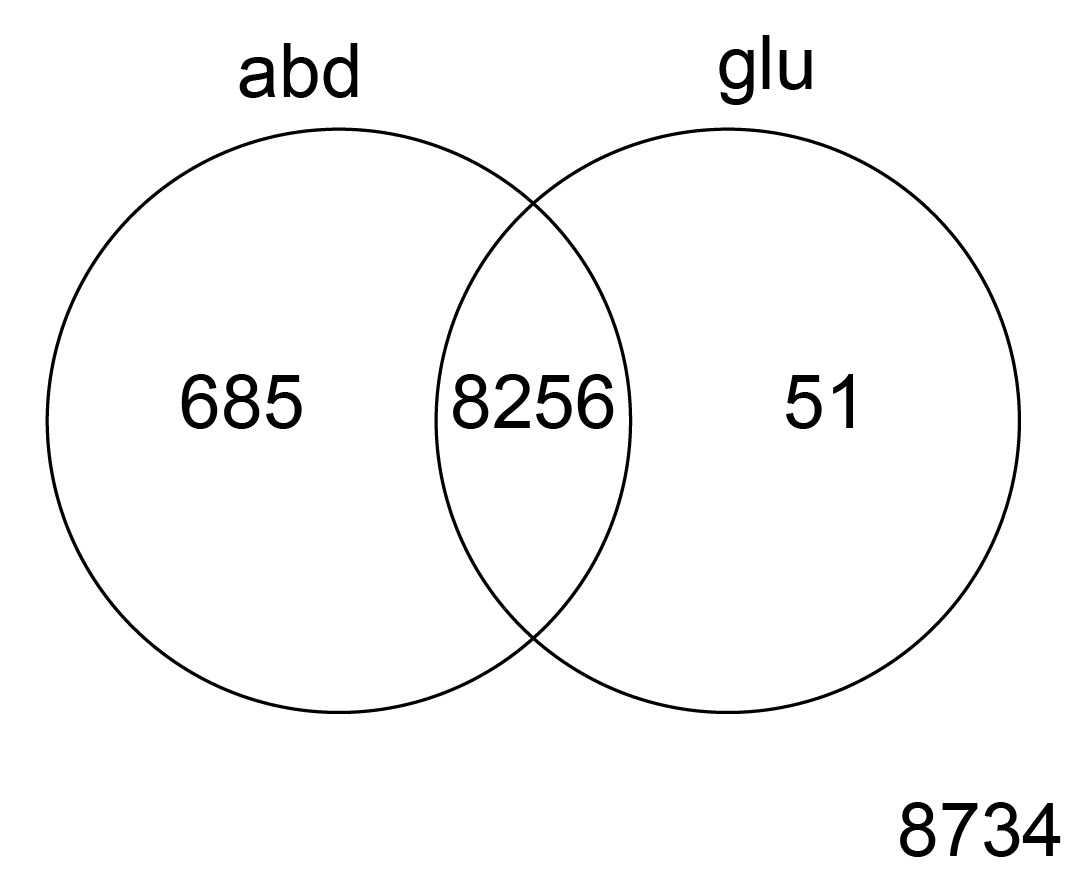

Supplement: Figure S4 — Venn diagram of number of expressed genes in MolOBB ABD and GLU. (TIF) [file pgen.1002505.s004.tif]

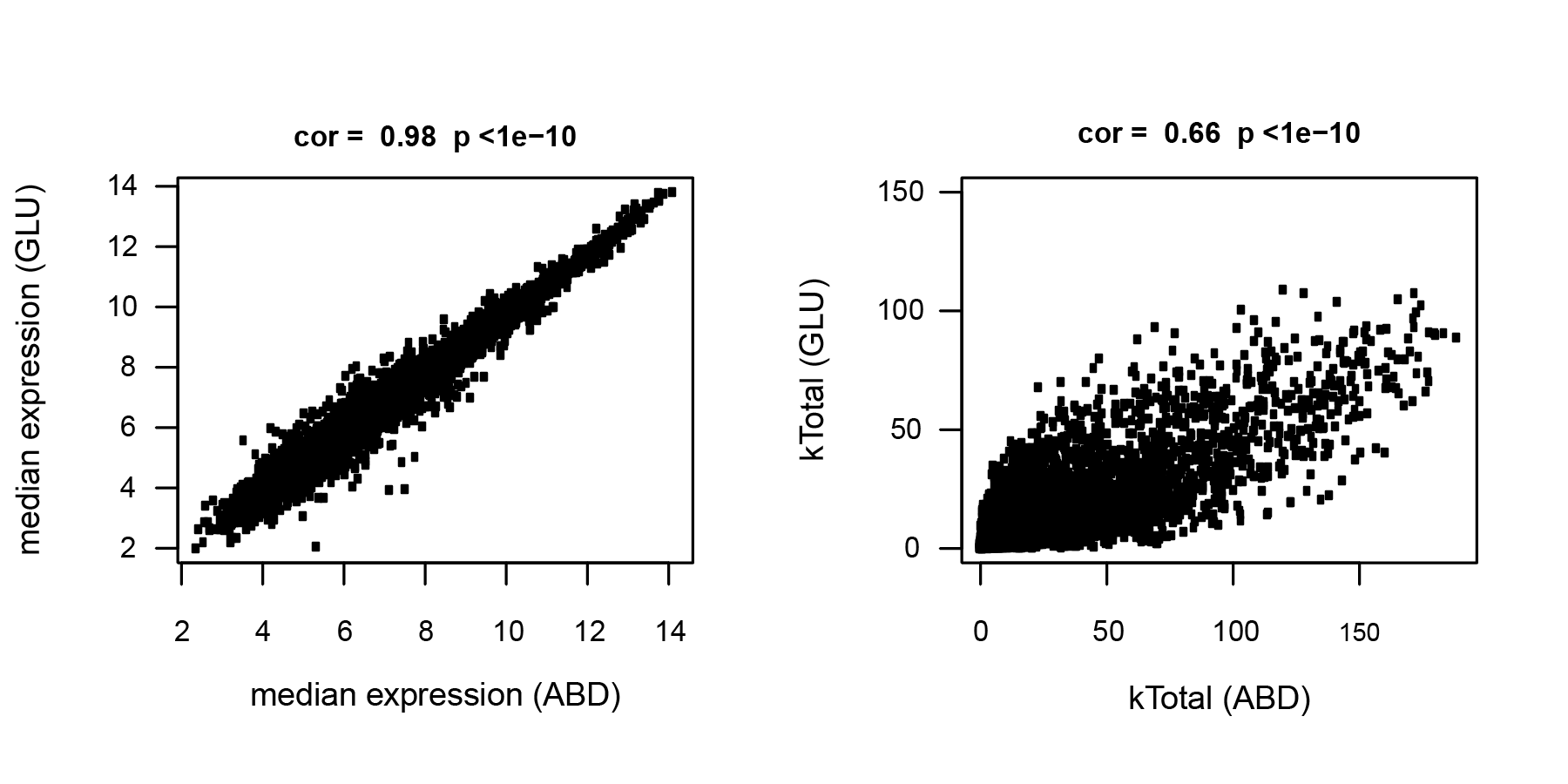

Supplement: Figure S5 — Preservation of genes across fat depots. A. Scatterplot of 8256 median expression levels for ABD versus GLU. B. Scatterplot of whole-network connectivities for ABD versus GLU. Each point corresponds to a gene expression level. Spearman correlations and the corresponding p-values are displayed in the title of each plot. The whole-network connectivity is strongly preserved between ABD and GLU. (TIF) [file pgen.1002505.s005.tif]

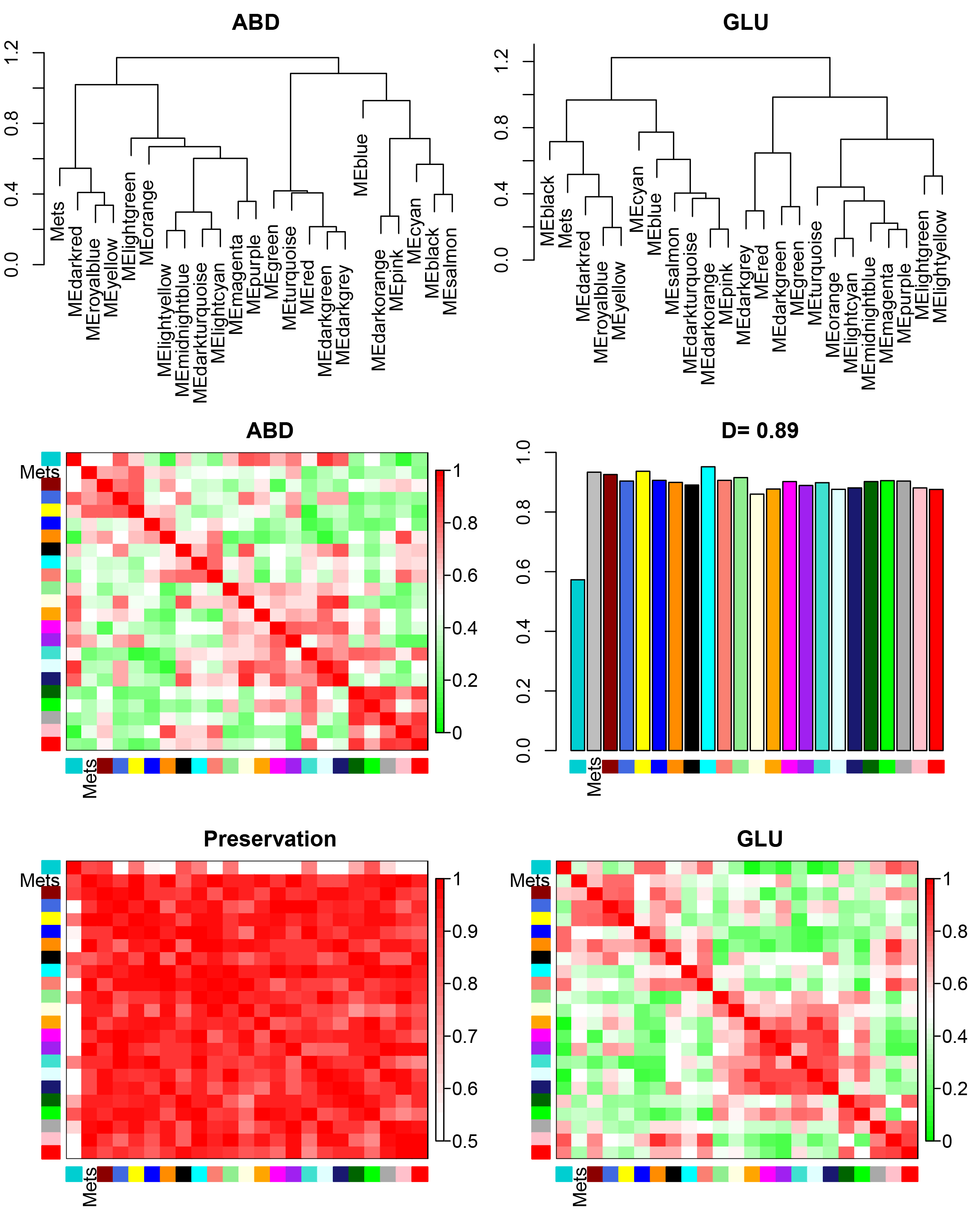

Supplement: Figure S6 — Differential eigengene network analysis across different depots. A, B Dendrograms of consensus module eigengenes in ABD and GLU. C, F.The heatmap plots of eigengene adjacencies in each eigengene network. Each row and column corresponds to one eigengene (labeled by consensus module color). Within each heatmap, red indicates high adjacency and green displays a low adjacency as shown by the color legend. D. Barplot shows the preservation of relationships of consensus eigengenes of the consensus modules. D represents the overall preservation measure. E. Adjacency heatmap for the pairwise preservation network of ABD (rows) and GLU (columns). Red indicates the adjacency. (TIF) [file pgen.1002505.s006.tif]

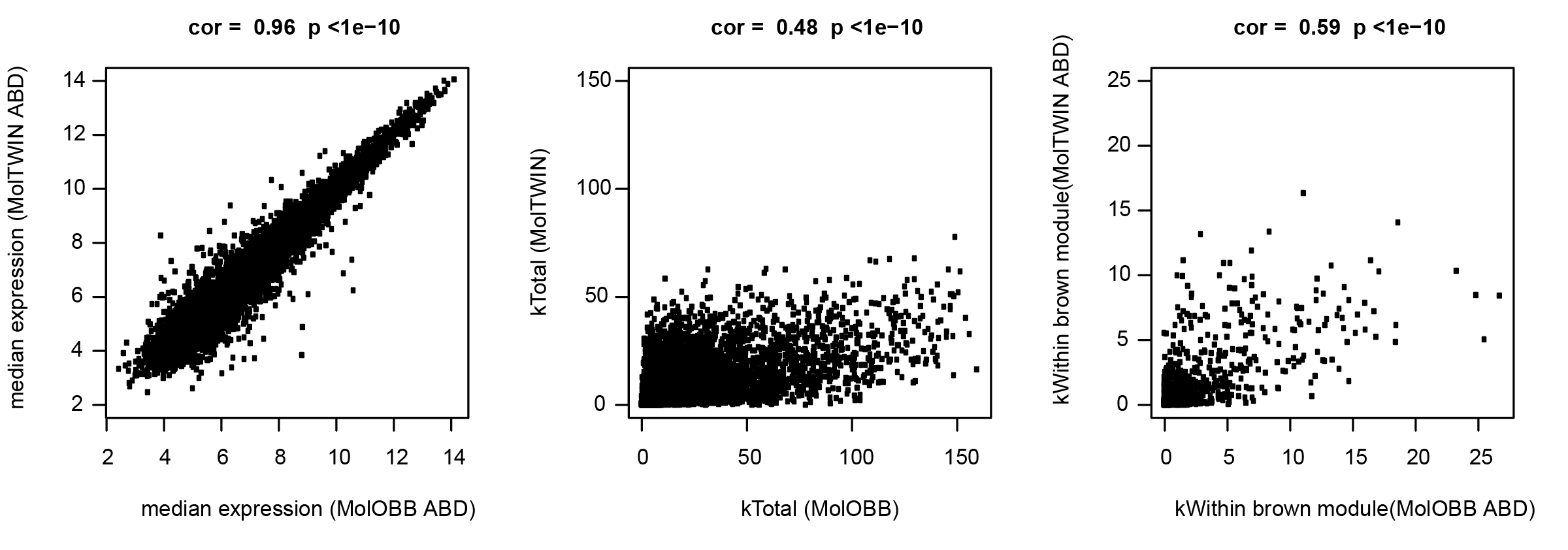

Supplement: Figure S7 — Preservation of genes across MolOBB and MolTWIN study. A. Scatterplot of 8242 median expression levels for MolOBB ABD versus MolTWIN ABD. B. Scatterplot of whole-network connectivities for MolOBB ABD versus MolTWIN ABD. C. Scatterplot of intramodular connectivities of genes in the brown module for MolOBB ABD versus MolTWIN ABD. Each point corresponds to a gene expression level. Spearman correlations and the corresponding p-values are displayed in the title of each plot. The whole-network connectivity and intramodular connectivities of genes in the brown module are preserved between MolTWIN and MolOBB. (TIF) [file pgen.1002505.s007.tif]
