# Supplementary material for: Coexpression Network Analysis in Abdominal and Gluteal Adipose Tissue Reveals Regulatory Genetic Loci for Metabolic Syndrome and Related Phenotypes
Source: PLoS Genet. 2012 Feb 23;8(2):e1002505. doi: 10.1371/journal.pgen.1002505 (PMC3285582; doi:10.1371/journal.pgen.1002505)
Supplement: Table S1 — Genes differentially expressed between MetS cases and controls in ABD (‘single gene analysis’). (DOC) [file pgen.1002505.s008.doc]

**Table S1** Genes differentially expressed between MetS cases and controls in ABD (‘single gene analysis’).

| *HGNC* | GENE_ID | MetS DE pvalue | MM | MM pvalue | Module | Consensus module |
| --- | --- | --- | --- | --- | --- | --- |
| *DEFB132* | 400830 | 2.4E-09 | 0.76 | 2.1E-11 | brown | yellow |
| *DARC* | 2532 | 1.7E-08 | 0.68 | 1.6E-08 | cyan | turquoise |
| *COX5A* | 9377 | 2.6E-08 | 0.75 | 7.4E-11 | brown | turquoise |
| *GINS1* | 9837 | 2.8E-08 | 0.77 | 1.1E-11 | cyan | yellow |
| *TMEM22* | 80723 | 3.7E-08 | 0.78 | 2.7E-12 | brown | turquoise |
| *SNCAIP* | 9627 | 5.2E-08 | 0.77 | 8.3E-12 | cyan | yellow |
| *C12orf39* | 80763 | 7.1E-08 | -0.78 | 3.7E-12 | cyan | yellow |
| *CCDC50* | 152137 | 7.6E-08 | 0.82 | 1.9E-14 | black | red |
| *OLFM1* | 10439 | 8.2E-08 | -0.61 | 7.5E-07 | brown | yellow |
| *ORMDL3* | 94103 | 8.3E-08 | 0.67 | 2.8E-08 | black | turquoise |
| *MKNK2* | 2872 | 8.8E-08 | -0.71 | 2.2E-09 | darkgrey | turquoise |
| *GALNTL1* | 57452 | 9.1E-08 | 0.70 | 5.2E-09 | cyan | yellow |
| *LTBP2* | 4053 | 1.1E-07 | -0.75 | 7.7E-11 | brown | yellow |
| *SLC7A10* | 56301 | 1.1E-07 | 0.87 | 1.5E-17 | brown | turquoise |
| *BACE2* | 25825 | 1.3E-07 | 0.71 | 1.3E-09 | darkred | yellow |
| *DECR1* | 1666 | 1.5E-07 | 0.85 | 4.8E-16 | brown | turquoise |
| *MDH2* | 4191 | 1.8E-07 | 0.75 | 4.9E-11 | brown | turquoise |
| *LPCAT4* | 254531 | 2.1E-07 | -0.75 | 7.5E-11 | brown | turquoise |
| *AZGP1* | 563 | 2.1E-07 | 0.86 | 4.9E-17 | brown | turquoise |
| *FAM126B* | 285172 | 2.2E-07 | 0.71 | 2.0E-09 | black | red |
| *RAB18* | 22931 | 2.7E-07 | 0.78 | 4.0E-12 | black | darkgreen |
| *TNFAIP8* | 25816 | 2.8E-07 | 0.69 | 7.4E-09 | pink | turquoise |
| *FAM102B* | 284611 | 2.9E-07 | 0.83 | 1.2E-14 | cyan | yellow |
| *KCTD10* | 83892 | 3.1E-07 | 0.76 | 3.4E-11 | darkgrey | yellow |
| *TPBG* | 7162 | 3.4E-07 | 0.84 | 3.5E-15 | cyan | yellow |
| *ACADM* | 34 | 3.7E-07 | 0.81 | 1.0E-13 | brown | turquoise |
| *ITGB5* | 3693 | 3.9E-07 | 0.75 | 5.7E-11 | darkgrey | yellow |
| *NDUFB5* | 4711 | 4.5E-07 | 0.86 | 1.1E-16 | brown | turquoise |
| *PDE3B* | 5140 | 4.7E-07 | -0.54 | 2.2E-05 | cyan | turquoise |
| *PPFIBP2* | 8495 | 5.7E-07 | -0.60 | 1.7E-06 | black | yellow |
| *PDLIM1* | 9124 | 5.8E-07 | 0.73 | 3.8E-10 | cyan | yellow |
| *CPXM2* | 119587 | 6.0E-07 | 0.53 | 3.8E-05 | darkgrey | yellow |
| *ACSS3* | 79611 | 7.3E-07 | 0.85 | 2.6E-16 | black | yellow |
| *NIPSNAP3B* | 55335 | 7.4E-07 | 0.89 | 1.1E-19 | black | red |
| *NUAK1* | 9891 | 8.0E-07 | -0.47 | 3.8E-04 | black | turquoise |
| *CHCHD3* | 54927 | 8.5E-07 | 0.76 | 4.2E-11 | pink | turquoise |
| *FAM89A* | 375061 | 8.7E-07 | 0.75 | 4.3E-11 | brown | turquoise |
| *PKP2* | 5318 | 8.8E-07 | -0.77 | 6.6E-12 | darkgrey | red |
| *PTP4A1* | 7803 | 9.7E-07 | 0.78 | 4.1E-12 | pink | darkgreen |
| *C15orf52* | 388115 | 9.8E-07 | 0.63 | 3.7E-07 | darkgrey | turquoise |
| *USP38* | 84640 | 9.8E-07 | 0.66 | 7.2E-08 | pink | darkgrey |
| *ETFA* | 2108 | 1.1E-06 | 0.86 | 4.3E-17 | black | turquoise |
| *ACAT1* | 38 | 1.1E-06 | 0.75 | 7.4E-11 | brown | turquoise |
| *PFKFB3* | 5209 | 1.2E-06 | 0.78 | 5.6E-12 | black | red |
| *ECHDC3* | 79746 | 1.2E-06 | 0.85 | 6.9E-16 | black | yellow |
| *GBAS* | 2631 | 1.3E-06 | 0.80 | 5.0E-13 | brown | turquoise |
| *NCRNA00188* | 125144 | 1.4E-06 | 0.68 | 1.3E-08 | black | turquoise |
| *LACTB2* | 51110 | 1.4E-06 | 0.78 | 2.4E-12 | brown | turquoise |
| *CD248* | 57124 | 1.4E-06 | 0.82 | 2.9E-14 | cyan | yellow |
| *ALDH1L1* | 10840 | 1.6E-06 | 0.83 | 4.3E-15 | brown | turquoise |
| *MLX* | 6945 | 1.6E-06 | 0.85 | 4.2E-16 | brown | turquoise |
| *HADH* | 3033 | 1.7E-06 | 0.88 | 1.3E-18 | black | red |
| *C7orf64* | 84060 | 1.8E-06 | 0.82 | 2.7E-14 | pink | turquoise |
| *ABCC10* | 89845 | 1.8E-06 | -0.59 | 3.0E-06 | blue | turquoise |
| *SFXN3* | 81855 | 1.9E-06 | 0.68 | 1.5E-08 | cyan | yellow |
| *CCDC80* | 151887 | 2.0E-06 | 0.82 | 6.0E-14 | cyan | yellow |
| *STOX1* | 219736 | 2.1E-06 | 0.76 | 3.9E-11 | black | yellow |
| *PDIA5* | 10954 | 2.2E-06 | 0.72 | 5.8E-10 | cyan | yellow |
| *CABC1* | 56997 | 2.2E-06 | 0.79 | 1.1E-12 | black | yellow |
| *HIBADH* | 11112 | 2.4E-06 | 0.88 | 8.7E-19 | brown | turquoise |
| *UQCRC2* | 7385 | 2.4E-06 | 0.89 | 1.0E-19 | brown | turquoise |
| *STBD1* | 8987 | 2.4E-06 | 0.85 | 8.6E-16 | brown | turquoise |
| *CDKN2C* | 1031 | 2.5E-06 | 0.93 | 1.3E-23 | brown | turquoise |
| *EXT2* | 2132 | 2.5E-06 | 0.66 | 7.5E-08 | cyan | yellow |
| *TSHZ3* | 57616 | 2.6E-06 | 0.63 | 2.7E-07 | cyan | turquoise |
| *ALDH1A3* | 220 | 2.7E-06 | 0.92 | 5.6E-23 | cyan | yellow |
| *SUCLG2* | 8801 | 2.8E-06 | 0.78 | 3.6E-12 | black | red |
| *LIAS* | 11019 | 2.9E-06 | 0.67 | 3.5E-08 | black | turquoise |
| *IDH3A* | 3419 | 3.0E-06 | 0.80 | 5.9E-13 | brown | turquoise |
| *TOB1* | 10140 | 3.1E-06 | 0.67 | 2.6E-08 | black | purple |
| *CNPY4* | 245812 | 3.1E-06 | 0.62 | 5.3E-07 | cyan | yellow |
| *SEPT8* | 23176 | 3.2E-06 | 0.70 | 2.7E-09 | darkgrey | yellow |
| *C1QTNF5* | 114902 | 3.4E-06 | -0.68 | 1.7E-08 | black | yellow |
| *WDR34* | 89891 | 3.4E-06 | -0.68 | 1.5E-08 | brown | royalblue |
| *RANBP9* | 10048 | 3.5E-06 | 0.80 | 6.2E-13 | black | darkgreen |
| *CD109* | 135228 | 3.6E-06 | -0.78 | 3.6E-12 | brown | yellow |
| *CYB561* | 1534 | 3.7E-06 | -0.70 | 4.4E-09 | black | yellow |
| *RCN3* | 57333 | 3.7E-06 | 0.69 | 6.9E-09 | cyan | royalblue |
| *ZNF654* | 55279 | 3.8E-06 | 0.79 | 1.5E-12 | black | red |
| *MRPL44* | 65080 | 3.8E-06 | 0.81 | 1.0E-13 | pink | turquoise |
| *IRAK2* | 3656 | 4.1E-06 | 0.66 | 7.1E-08 | black | red |
| *TF* | 7018 | 4.2E-06 | 0.60 | 1.3E-06 | brown | turquoise |
| *GRPEL1* | 80273 | 4.2E-06 | 0.79 | 1.3E-12 | brown | turquoise |
| *PRKAR2B* | 5577 | 4.3E-06 | 0.78 | 3.3E-12 | black | red |
| *CLIP2* | 7461 | 4.4E-06 | -0.71 | 2.0E-09 | brown | royalblue |
| *VNN1* | 8876 | 4.7E-06 | -0.72 | 1.2E-09 | blue | NA |
| *COL16A1* | 1307 | 5.1E-06 | 0.63 | 2.9E-07 | cyan | yellow |
| *PLXDC1* | 57125 | 5.4E-06 | 0.70 | 5.0E-09 | cyan | yellow |
| *NTRK3* | 4916 | 5.5E-06 | -0.73 | 5.5E-10 | cyan | turquoise |
| *CITED2* | 10370 | 5.7E-06 | 0.73 | 4.3E-10 | black | red |
| *CKB* | 1152 | 5.7E-06 | 0.83 | 7.9E-15 | brown | turquoise |
| *GPN3* | 51184 | 5.9E-06 | 0.87 | 2.4E-17 | pink | turquoise |
| *WDR7* | 23335 | 6.0E-06 | -0.46 | 5.3E-04 | black | yellow |
| *NDRG4* | 65009 | 6.0E-06 | -0.76 | 2.4E-11 | darkgrey | red |
| *STX11* | 8676 | 6.0E-06 | 0.66 | 6.7E-08 | brown | turquoise |
| *CHP* | 11261 | 6.2E-06 | 0.64 | 1.5E-07 | blue | turquoise |
| *TM7SF2* | 7108 | 6.2E-06 | -0.76 | 4.0E-11 | cyan | turquoise |
| *HSBP1* | 3281 | 6.8E-06 | -0.67 | 2.7E-08 | blue | turquoise |
| *NAGA* | 4668 | 6.8E-06 | 0.80 | 3.8E-13 | cyan | yellow |
| *PRKAR1A* | 5573 | 6.8E-06 | 0.71 | 1.4E-09 | black | darkgreen |
| *PGAP3* | 93210 | 6.8E-06 | -0.68 | 1.3E-08 | brown | turquoise |
| *ARRDC4* | 91947 | 7.0E-06 | 0.75 | 8.3E-11 | cyan | yellow |
| *MARCH5* | 54708 | 7.2E-06 | 0.74 | 1.4E-10 | pink | turquoise |
| *CTGF* | 1490 | 7.4E-06 | -0.80 | 4.4E-13 | brown | yellow |
| *OLFML2B* | 25903 | 7.5E-06 | 0.65 | 1.4E-07 | cyan | yellow |
| *ITGAV* | 3685 | 7.6E-06 | 0.68 | 1.4E-08 | cyan | red |
| *ITIH5* | 80760 | 7.9E-06 | 0.84 | 2.7E-15 | darkgrey | purple |
| *SPTAN1* | 6709 | 8.0E-06 | 0.73 | 3.0E-10 | green | royalblue |
| *LDHD* | 197257 | 8.1E-06 | 0.89 | 4.7E-19 | brown | turquoise |
| *CADM1* | 23705 | 8.1E-06 | 0.71 | 1.4E-09 | brown | turquoise |
| *RCOR1* | 23186 | 8.4E-06 | -0.67 | 2.4E-08 | darkgrey | red |
| *PDK2* | 5164 | 8.4E-06 | 0.79 | 1.4E-12 | black | red |
| *MRPL32* | 64983 | 8.4E-06 | 0.70 | 4.4E-09 | black | turquoise |
| *KRCC1* | 51315 | 8.6E-06 | 0.90 | 2.5E-20 | black | red |
| *ALPK3* | 57538 | 8.6E-06 | 0.83 | 9.1E-15 | brown | yellow |
| *SLC19A3* | 80704 | 8.8E-06 | 0.79 | 1.1E-12 | brown | turquoise |
| *SELP* | 6403 | 9.3E-06 | -0.67 | 2.9E-08 | blue | turquoise |
| *HTRA1* | 5654 | 9.4E-06 | 0.81 | 1.9E-13 | darkgrey | yellow |
| *INMT* | 11185 | 9.5E-06 | 0.69 | 6.3E-09 | darkgrey | yellow |
| *AKR1B1* | 231 | 9.7E-06 | 0.83 | 9.3E-15 | cyan | yellow |
| *MRS2* | 57380 | 9.7E-06 | 0.80 | 5.0E-13 | blue | turquoise |
| *THYN1* | 29087 | 9.8E-06 | 0.82 | 5.6E-14 | black | turquoise |
| *CARD6* | 84674 | 9.8E-06 | 0.83 | 1.2E-14 | cyan | yellow |
| *THADA* | 63892 | 9.9E-06 | 0.84 | 1.9E-15 | cyan | yellow |
| *DFNA5* | 1687 | 1.0E-05 | 0.67 | 3.1E-08 | cyan | yellow |
| *AGPAT9* | 84803 | 1.0E-05 | -0.69 | 8.0E-09 | darkgrey | yellow |
| *NFU1* | 27247 | 1.1E-05 | 0.78 | 2.8E-12 | brown | turquoise |
| *NDUFA7* | 4701 | 1.1E-05 | 0.78 | 2.8E-12 | brown | turquoise |
| *NDUFS4* | 4724 | 1.1E-05 | 0.79 | 8.9E-13 | blue | turquoise |
| *BPHL* | 670 | 1.1E-05 | 0.81 | 7.5E-14 | brown | turquoise |
| *COX5B* | 1329 | 1.2E-05 | 0.79 | 1.0E-12 | blue | turquoise |
| *GPD1L* | 23171 | 1.2E-05 | 0.90 | 1.3E-20 | black | yellow |
| *FUCA1* | 2517 | 1.2E-05 | 0.86 | 4.1E-17 | cyan | yellow |
| *C7orf46* | 340277 | 1.2E-05 | 0.83 | 6.7E-15 | black | yellow |
| *EPB41L4B* | 54566 | 1.2E-05 | 0.83 | 1.4E-14 | black | red |
| *C18orf21* | 83608 | 1.2E-05 | 0.61 | 1.2E-06 | blue | turquoise |
| *TJP2* | 9414 | 1.2E-05 | 0.70 | 4.0E-09 | black | yellow |
| *ACVR1C* | 130399 | 1.3E-05 | 0.74 | 1.2E-10 | black | yellow |
| *RGL1* | 23179 | 1.3E-05 | 0.68 | 1.4E-08 | darkgrey | yellow |
| *KLF15* | 28999 | 1.3E-05 | 0.66 | 6.2E-08 | blue | turquoise |
| *AOC2* | 314 | 1.3E-05 | -0.67 | 2.2E-08 | black | purple |
| *WISP2* | 8839 | 1.3E-05 | 0.81 | 2.1E-13 | cyan | yellow |
| *RTN3* | 10313 | 1.4E-05 | 0.75 | 8.7E-11 | brown | turquoise |
| *COL5A2* | 1290 | 1.4E-05 | -0.69 | 6.0E-09 | black | yellow |
| *FGF2* | 2247 | 1.4E-05 | 0.84 | 3.9E-15 | black | red |
| *GLUL* | 2752 | 1.4E-05 | 0.79 | 1.4E-12 | black | yellow |
| *TNC* | 3371 | 1.4E-05 | 0.81 | 6.6E-14 | cyan | yellow |
| *VWA5A* | 4013 | 1.4E-05 | 0.77 | 9.5E-12 | cyan | yellow |
| *UBE2B* | 7320 | 1.4E-05 | 0.65 | 1.1E-07 | black | red |
| *ST3GAL5* | 8869 | 1.4E-05 | -0.67 | 4.1E-08 | blue | yellow |
| *CIDEA* | 1149 | 1.5E-05 | 0.81 | 1.0E-13 | black | red |
| *EIF4E3* | 317649 | 1.5E-05 | 0.60 | 1.4E-06 | black | darkgreen |
| *IL17D* | 53342 | 1.5E-05 | 0.82 | 4.7E-14 | cyan | yellow |
| *MFAP5* | 8076 | 1.5E-05 | 0.73 | 5.0E-10 | cyan | yellow |
| *GMPPA* | 29926 | 1.6E-05 | 0.49 | 1.9E-04 | cyan | royalblue |
| *FAM174A* | 345757 | 1.6E-05 | 0.64 | 1.5E-07 | blue | turquoise |
| *ADAP2* | 55803 | 1.6E-05 | 0.85 | 4.5E-16 | cyan | yellow |
| *LRRK1* | 79705 | 1.6E-05 | 0.74 | 1.5E-10 | cyan | yellow |
| *DHRS12* | 79758 | 1.6E-05 | 0.65 | 8.0E-08 | blue | turquoise |
| *ST3GAL6* | 10402 | 1.7E-05 | -0.58 | 5.4E-06 | darkgrey | yellow |
| *BCL2L13* | 23786 | 1.7E-05 | 0.67 | 3.0E-08 | blue | darkgreen |
| *PYGM* | 5837 | 1.7E-05 | -0.78 | 3.4E-12 | cyan | yellow |
| *MRPS30* | 10884 | 1.8E-05 | 0.58 | 4.2E-06 | black | turquoise |
| *LSM6* | 11157 | 1.8E-05 | 0.70 | 3.4E-09 | blue | turquoise |
| *ELN* | 2006 | 1.8E-05 | 0.58 | 5.1E-06 | cyan | royalblue |
| *ISOC1* | 51015 | 1.8E-05 | 0.85 | 3.9E-16 | brown | turquoise |
| *KIAA1239* | 57495 | 1.8E-05 | 0.61 | 1.1E-06 | black | NA |
| *ST13* | 6767 | 1.8E-05 | 0.80 | 4.9E-13 | black | red |
| *CCNH* | 902 | 1.8E-05 | 0.87 | 3.1E-17 | brown | turquoise |
| *FARP1* | 10160 | 1.9E-05 | 0.68 | 1.7E-08 | cyan | royalblue |
| *EIF1* | 10209 | 1.9E-05 | 0.75 | 4.6E-11 | black | red |
| *STK39* | 27347 | 1.9E-05 | 0.56 | 8.9E-06 | pink | turquoise |
| *NDUFA6* | 4700 | 1.9E-05 | 0.68 | 1.8E-08 | brown | turquoise |
| *RARRES2* | 5919 | 1.9E-05 | 0.83 | 8.9E-15 | brown | turquoise |
| *WDR54* | 84058 | 1.9E-05 | -0.64 | 2.2E-07 | brown | turquoise |
| *EFCAB8* | 388795 | 2.0E-05 | -0.70 | 3.3E-09 | black | turquoise |
| *DNAJA2* | 10294 | 2.1E-05 | 0.85 | 2.5E-16 | pink | green |
| *ANO6* | 196527 | 2.1E-05 | 0.83 | 5.6E-15 | black | turquoise |
| *CENPV* | 201161 | 2.1E-05 | 0.75 | 6.4E-11 | black | turquoise |
| *POLR2E* | 5434 | 2.1E-05 | 0.64 | 2.2E-07 | brown | turquoise |
| *S100A6* | 6277 | 2.1E-05 | -0.85 | 5.3E-16 | brown | yellow |
| *CCDC109A* | 90550 | 2.1E-05 | -0.50 | 1.2E-04 | brown | yellow |
| *GLCCI1* | 113263 | 2.3E-05 | 0.67 | 3.8E-08 | blue | yellow |
| *DCI* | 1632 | 2.3E-05 | 0.81 | 9.5E-14 | blue | turquoise |
| *GATA3* | 2625 | 2.3E-05 | 0.70 | 4.1E-09 | brown | yellow |
| *RND3* | 390 | 2.3E-05 | -0.76 | 1.7E-11 | brown | yellow |
| *MAP1B* | 4131 | 2.3E-05 | -0.78 | 4.1E-12 | brown | turquoise |
| *GPT2* | 84706 | 2.3E-05 | -0.74 | 1.5E-10 | cyan | yellow |
| *ASAP2* | 8853 | 2.3E-05 | -0.51 | 7.9E-05 | brown | yellow |
| *CALML4* | 91860 | 2.3E-05 | -0.73 | 2.5E-10 | black | yellow |
| *PLA2G16* | 11145 | 2.4E-05 | 0.75 | 1.0E-10 | black | turquoise |
| *SDHD* | 6392 | 2.4E-05 | 0.80 | 3.5E-13 | brown | turquoise |
| *GPR137B* | 7107 | 2.4E-05 | 0.87 | 2.3E-17 | cyan | yellow |
| *C7orf30* | 115416 | 2.5E-05 | 0.81 | 1.5E-13 | brown | turquoise |
| *PFDN2* | 5202 | 2.5E-05 | 0.74 | 2.3E-10 | blue | turquoise |
| *MCCC2* | 64087 | 2.5E-05 | 0.64 | 1.5E-07 | blue | NA |
| *PRTFDC1* | 56952 | 2.6E-05 | 0.73 | 3.7E-10 | cyan | yellow |
| *MARCH7* | 64844 | 2.6E-05 | 0.72 | 9.5E-10 | black | red |
| *OSBP* | 5007 | 2.7E-05 | 0.74 | 2.5E-10 | black | red |
| *BMP2K* | 55589 | 2.7E-05 | -0.61 | 9.2E-07 | black | yellow |
| *MOSC1* | 64757 | 2.7E-05 | 0.92 | 3.9E-23 | brown | turquoise |
| *ZWILCH* | 55055 | 2.8E-05 | -0.73 | 3.2E-10 | brown | turquoise |
| *BNIP3* | 664 | 2.8E-05 | 0.77 | 6.4E-12 | black | turquoise |
| *SUCLG1* | 8802 | 2.8E-05 | 0.89 | 2.1E-19 | brown | turquoise |
| *UBTD2* | 92181 | 2.8E-05 | 0.71 | 1.3E-09 | cyan | yellow |
| *CYP2R1* | 120227 | 2.9E-05 | -0.77 | 7.4E-12 | brown | turquoise |
| *MRPL39* | 54148 | 2.9E-05 | 0.78 | 3.3E-12 | pink | turquoise |
| *ISCA1* | 81689 | 2.9E-05 | 0.66 | 7.6E-08 | brown | turquoise |
| *MINA* | 84864 | 2.9E-05 | 0.64 | 2.5E-07 | purple | darkgrey |
| *NAALAD2* | 10003 | 3.0E-05 | 0.85 | 6.4E-16 | black | yellow |
| *LHFPL2* | 10184 | 3.0E-05 | 0.86 | 8.1E-17 | cyan | yellow |
| *CFL2* | 1073 | 3.0E-05 | 0.78 | 5.2E-12 | brown | turquoise |
| *C22orf9* | 23313 | 3.0E-05 | 0.83 | 7.7E-15 | cyan | yellow |
| *FN1* | 2335 | 3.0E-05 | -0.77 | 7.6E-12 | brown | yellow |
| *CHCHD10* | 400916 | 3.0E-05 | 0.81 | 2.0E-13 | brown | turquoise |
| *NDUFB8* | 4714 | 3.0E-05 | 0.74 | 2.1E-10 | brown | turquoise |
| *MRPS7* | 51081 | 3.0E-05 | 0.85 | 7.8E-16 | brown | turquoise |
| *KIAA1370* | 56204 | 3.0E-05 | 0.77 | 1.5E-11 | black | red |
| *FUCA2* | 2519 | 3.1E-05 | 0.78 | 3.6E-12 | cyan | yellow |
| *TAX1BP3* | 30851 | 3.1E-05 | -0.71 | 1.8E-09 | black | yellow |
| *SLMO2* | 51012 | 3.1E-05 | 0.76 | 1.7E-11 | pink | green |
| *RBP4* | 5950 | 3.1E-05 | 0.72 | 7.9E-10 | midnightblue | turquoise |
| *MLPH* | 79083 | 3.1E-05 | -0.64 | 2.2E-07 | brown | turquoise |
| *OMD* | 4958 | 3.2E-05 | 0.66 | 7.5E-08 | cyan | yellow |
| *BOK* | 666 | 3.2E-05 | 0.71 | 1.9E-09 | brown | turquoise |
| *CYB5A* | 1528 | 3.3E-05 | 0.76 | 2.9E-11 | brown | turquoise |
| *ACAD8* | 27034 | 3.3E-05 | 0.83 | 8.3E-15 | brown | turquoise |
| *SUMF1* | 285362 | 3.3E-05 | 0.70 | 4.4E-09 | cyan | yellow |
| *MTHFD2L* | 441024 | 3.3E-05 | 0.71 | 2.3E-09 | blue | yellow |
| *VASH1* | 22846 | 3.4E-05 | -0.64 | 1.7E-07 | black | darkgreen |
| *DDB2* | 1643 | 3.5E-05 | -0.73 | 2.7E-10 | brown | turquoise |
| *RHOB* | 388 | 3.5E-05 | -0.57 | 6.7E-06 | darkgrey | NA |
| *WRB* | 7485 | 3.5E-05 | 0.75 | 1.0E-10 | pink | darkgreen |
| *C7orf55* | 154791 | 3.6E-05 | 0.79 | 9.8E-13 | brown | turquoise |
| *MANBA* | 4126 | 3.6E-05 | -0.76 | 4.1E-11 | blue | yellow |
| *CD276* | 80381 | 3.6E-05 | 0.66 | 6.7E-08 | cyan | yellow |
| *CAT* | 847 | 3.6E-05 | 0.74 | 1.2E-10 | black | red |
| *PLXDC2* | 84898 | 3.6E-05 | 0.76 | 2.0E-11 | cyan | yellow |
| *GPC1* | 2817 | 3.7E-05 | -0.71 | 2.4E-09 | black | yellow |
| *SDHB* | 6390 | 3.7E-05 | 0.82 | 6.0E-14 | brown | turquoise |
| *CHKA* | 1119 | 3.8E-05 | 0.71 | 2.1E-09 | black | turquoise |
| *TATDN3* | 128387 | 3.8E-05 | 0.71 | 1.6E-09 | pink | green |
| *EIF3E* | 3646 | 3.8E-05 | 0.61 | 1.2E-06 | black | turquoise |
| *MOCS1* | 4337 | 3.8E-05 | 0.85 | 8.9E-16 | black | red |
| *ATP5B* | 506 | 3.8E-05 | 0.90 | 1.3E-20 | brown | turquoise |
| *ARPC1A* | 10552 | 3.9E-05 | 0.69 | 1.0E-08 | pink | darkgreen |
| *CCDC104* | 112942 | 3.9E-05 | 0.80 | 6.0E-13 | darkred | darkred |
| *S100A16* | 140576 | 3.9E-05 | -0.69 | 1.1E-08 | black | red |
| *NDUFAF4* | 29078 | 3.9E-05 | 0.72 | 6.6E-10 | brown | turquoise |
| *GNA14* | 9630 | 3.9E-05 | 0.62 | 5.6E-07 | blue | turquoise |
| *ABCC5* | 10057 | 4.0E-05 | -0.75 | 5.9E-11 | brown | turquoise |
| *ADAM33* | 80332 | 4.0E-05 | -0.40 | 3.1E-03 | brown | royalblue |
| *C6orf129* | 154467 | 4.1E-05 | -0.64 | 2.3E-07 | black | royalblue |
| *GNAI1* | 2770 | 4.1E-05 | 0.82 | 3.1E-14 | black | yellow |
| *SMARCA5* | 8467 | 4.2E-05 | 0.80 | 4.2E-13 | purple | red |
| *CALCRL* | 10203 | 4.3E-05 | 0.61 | 8.5E-07 | blue | yellow |
| *DPYSL3* | 1809 | 4.3E-05 | 0.73 | 2.7E-10 | darkred | darkred |
| *LIN7C* | 55327 | 4.3E-05 | 0.78 | 4.3E-12 | pink | turquoise |
| *REEP5* | 7905 | 4.3E-05 | -0.52 | 4.7E-05 | black | yellow |
| *FYTTD1* | 84248 | 4.3E-05 | 0.69 | 9.5E-09 | darkturquoise | green |
| *NDUFB4* | 4710 | 4.4E-05 | 0.75 | 6.1E-11 | blue | turquoise |
| *CAPN7* | 23473 | 4.5E-05 | 0.80 | 4.8E-13 | pink | turquoise |
| *EIF3K* | 27335 | 4.5E-05 | 0.67 | 4.1E-08 | brown | turquoise |
| *EIF2A* | 83939 | 4.5E-05 | 0.70 | 2.8E-09 | black | turquoise |
| *CMTM3* | 123920 | 4.6E-05 | -0.80 | 5.6E-13 | brown | yellow |
| *WDR48* | 57599 | 4.6E-05 | 0.62 | 4.5E-07 | black | darkgreen |
| *CHD3* | 1107 | 4.7E-05 | -0.75 | 7.6E-11 | pink | NA |
| *THAP6* | 152815 | 4.7E-05 | 0.51 | 8.0E-05 | black | blue |
| *PPID* | 5481 | 4.7E-05 | 0.79 | 1.7E-12 | pink | green |
| *RAB34* | 83871 | 4.7E-05 | 0.74 | 1.5E-10 | cyan | yellow |
| *OSBPL1A* | 114876 | 4.9E-05 | 0.85 | 3.0E-16 | brown | turquoise |
| *MRPL45* | 84311 | 4.9E-05 | 0.64 | 2.3E-07 | blue | turquoise |
| *DBT* | 1629 | 5.0E-05 | 0.81 | 1.4E-13 | brown | turquoise |
| *TMED3* | 23423 | 5.0E-05 | 0.77 | 8.2E-12 | cyan | yellow |
| *TGDS* | 23483 | 5.0E-05 | 0.83 | 1.2E-14 | black | red |
| *S100A1* | 6271 | 5.0E-05 | 0.78 | 3.7E-12 | blue | turquoise |
| *CHUK* | 1147 | 5.1E-05 | 0.55 | 1.8E-05 | pink | turquoise |
| *VPS37A* | 137492 | 5.1E-05 | 0.77 | 7.6E-12 | pink | green |
| *EDN1* | 1906 | 5.2E-05 | -0.62 | 4.7E-07 | royalblue | turquoise |
| *ELOVL5* | 60481 | 5.2E-05 | 0.78 | 3.9E-12 | brown | turquoise |
| *TUBA1A* | 7846 | 5.2E-05 | -0.84 | 3.4E-15 | brown | turquoise |
| *ACSL1* | 2180 | 5.3E-05 | 0.86 | 3.5E-17 | brown | turquoise |
| *C3AR1* | 719 | 5.3E-05 | 0.88 | 8.2E-19 | cyan | yellow |
| *GHITM* | 27069 | 5.4E-05 | 0.84 | 9.6E-16 | royalblue | turquoise |
| *IQGAP2* | 10788 | 5.5E-05 | -0.90 | 1.8E-20 | blue | yellow |
| *ADCY7* | 113 | 5.5E-05 | -0.69 | 9.6E-09 | blue | yellow |
| *PLXND1* | 23129 | 5.5E-05 | 0.42 | 1.7E-03 | darkgrey | red |
| *RPAIN* | 84268 | 5.5E-05 | 0.78 | 2.3E-12 | black | turquoise |
| *AKAP7* | 9465 | 5.5E-05 | 0.74 | 2.3E-10 | brown | turquoise |
| *FSTL1* | 11167 | 5.6E-05 | 0.75 | 8.4E-11 | cyan | yellow |
| *C6orf120* | 387263 | 5.6E-05 | 0.81 | 1.7E-13 | pink | green |
| *ALCAM* | 214 | 5.7E-05 | 0.83 | 9.7E-15 | cyan | yellow |
| *DAPK2* | 23604 | 5.7E-05 | 0.85 | 5.2E-16 | black | red |
| *MRPL15* | 29088 | 5.7E-05 | 0.72 | 6.3E-10 | brown | lightcyan |
| *RAI14* | 26064 | 5.9E-05 | 0.54 | 2.5E-05 | cyan | turquoise |
| *UCHL1* | 7345 | 6.0E-05 | 0.71 | 1.3E-09 | darkgrey | yellow |
| *RABGGTB* | 5876 | 6.1E-05 | 0.80 | 7.0E-13 | black | darkgreen |
| *SUOX* | 6821 | 6.1E-05 | 0.78 | 2.2E-12 | brown | turquoise |
| *HMOX1* | 3162 | 6.2E-05 | 0.77 | 7.4E-12 | cyan | yellow |
| *TFPI* | 7035 | 6.2E-05 | 0.74 | 2.1E-10 | cyan | yellow |
| *TWIST1* | 7291 | 6.2E-05 | 0.87 | 2.8E-17 | black | red |
| *PPP1R15B* | 84919 | 6.2E-05 | 0.65 | 9.1E-08 | black | red |
| *RTCD1* | 8634 | 6.2E-05 | 0.75 | 7.5E-11 | brown | turquoise |
| *G0S2* | 50486 | 6.3E-05 | 0.72 | 8.3E-10 | brown | turquoise |
| *SLC27A2* | 11001 | 6.4E-05 | 0.83 | 7.7E-15 | black | NA |
| *FCGR2B* | 2213 | 6.4E-05 | 0.75 | 7.0E-11 | cyan | yellow |
| *BDH2* | 56898 | 6.4E-05 | 0.62 | 4.8E-07 | cyan | yellow |
| *EMILIN2* | 84034 | 6.4E-05 | 0.84 | 3.0E-15 | cyan | yellow |
| *COPS2* | 9318 | 6.4E-05 | 0.81 | 2.0E-13 | black | red |
| *BAG4* | 9530 | 6.4E-05 | 0.68 | 1.3E-08 | brown | turquoise |
| *ADNP* | 23394 | 6.5E-05 | 0.69 | 9.2E-09 | black | red |
| *GYS2* | 2998 | 6.5E-05 | 0.65 | 1.1E-07 | brown | turquoise |
| *PGM1* | 5236 | 6.6E-05 | 0.80 | 2.6E-13 | brown | turquoise |
| *OXR1* | 55074 | 6.6E-05 | 0.75 | 4.9E-11 | pink | green |
| *UBE2G1* | 7326 | 6.6E-05 | 0.70 | 4.1E-09 | pink | turquoise |
| *SH3BP4* | 23677 | 6.7E-05 | -0.49 | 2.0E-04 | brown | green |
| *GAA* | 2548 | 6.7E-05 | -0.69 | 9.8E-09 | blue | turquoise |
| *NUDT21* | 11051 | 6.8E-05 | 0.61 | 9.4E-07 | black | darkgreen |
| *FH* | 2271 | 6.8E-05 | 0.78 | 5.8E-12 | royalblue | turquoise |
| *OGN* | 4969 | 6.8E-05 | 0.58 | 3.7E-06 | cyan | yellow |
| *C6orf203* | 51250 | 6.8E-05 | 0.70 | 5.2E-09 | blue | turquoise |
| *PRCP* | 5547 | 6.9E-05 | -0.61 | 8.0E-07 | brown | yellow |
| *GKAP1* | 80318 | 7.0E-05 | 0.86 | 1.0E-16 | black | red |
| *STAT5B* | 6777 | 7.1E-05 | 0.61 | 7.7E-07 | brown | turquoise |
| *PMM1* | 5372 | 7.2E-05 | 0.84 | 1.4E-15 | black | red |
| *ATP10D* | 57205 | 7.2E-05 | -0.63 | 3.2E-07 | brown | turquoise |
| *PLAC9* | 219348 | 7.4E-05 | 0.67 | 2.5E-08 | darkgrey | yellow |
| *SERPINE2* | 5270 | 7.4E-05 | -0.68 | 2.1E-08 | black | red |
| *PECR* | 55825 | 7.6E-05 | 0.80 | 4.1E-13 | brown | turquoise |
| *POLK* | 51426 | 7.8E-05 | 0.73 | 4.5E-10 | pink | darkgreen |
| *ATP8B4* | 79895 | 7.8E-05 | 0.71 | 2.0E-09 | black | red |
| *TMEM93* | 83460 | 7.8E-05 | 0.74 | 1.5E-10 | brown | turquoise |
| *FEZ1* | 9638 | 7.8E-05 | 0.73 | 2.8E-10 | cyan | yellow |
| *CSF1R* | 1436 | 7.9E-05 | 0.77 | 1.6E-11 | cyan | yellow |
| *ANXA3* | 306 | 7.9E-05 | -0.44 | 7.9E-04 | darkred | turquoise |
| *NDUFA10* | 4705 | 7.9E-05 | 0.84 | 2.2E-15 | brown | turquoise |
| *PRRX1* | 5396 | 7.9E-05 | 0.77 | 6.4E-12 | cyan | yellow |
| *BNIP3L* | 665 | 8.1E-05 | -0.57 | 6.8E-06 | cyan | turquoise |
| *VEGFA* | 7422 | 8.1E-05 | 0.72 | 1.2E-09 | black | yellow |
| *HSDL2* | 84263 | 8.2E-05 | 0.81 | 1.0E-13 | brown | turquoise |
| *ANXA4* | 307 | 8.3E-05 | 0.81 | 1.9E-13 | cyan | darkred |
| *RCL1* | 10171 | 8.4E-05 | 0.80 | 3.1E-13 | black | turquoise |
| *PHLPP1* | 23239 | 8.4E-05 | 0.82 | 2.7E-14 | black | red |
| *ZNF462* | 58499 | 8.4E-05 | -0.52 | 6.4E-05 | black | darkgreen |
| *CTSO* | 1519 | 8.5E-05 | 0.53 | 3.5E-05 | cyan | yellow |
| *CRLS1* | 54675 | 8.5E-05 | 0.72 | 8.9E-10 | black | turquoise |
| *SLAIN2* | 57606 | 8.5E-05 | 0.70 | 4.5E-09 | pink | green |
| *EXOSC3* | 51010 | 8.6E-05 | 0.76 | 2.9E-11 | pink | green |
| *CNBP* | 7555 | 8.6E-05 | 0.75 | 9.3E-11 | black | red |
| *QSOX1* | 5768 | 8.7E-05 | -0.75 | 4.9E-11 | brown | turquoise |
| *COX7A2* | 1347 | 8.8E-05 | 0.71 | 1.8E-09 | blue | turquoise |
| *LPPR4* | 9890 | 8.8E-05 | -0.45 | 6.0E-04 | black | NA |
| *CTHRC1* | 115908 | 8.9E-05 | 0.78 | 5.4E-12 | cyan | yellow |
| *SLC43A3* | 29015 | 8.9E-05 | 0.78 | 3.8E-12 | cyan | yellow |
| *HTR2B* | 3357 | 8.9E-05 | 0.63 | 2.8E-07 | cyan | yellow |
| *ABHD5* | 51099 | 8.9E-05 | 0.74 | 2.1E-10 | brown | turquoise |
| *TRMT12* | 55039 | 8.9E-05 | 0.47 | 3.6E-04 | darkgrey | turquoise |
| *UBE2W* | 55284 | 8.9E-05 | 0.68 | 1.2E-08 | pink | turquoise |
| *FAM129B* | 64855 | 8.9E-05 | -0.74 | 1.4E-10 | purple | royalblue |
| *ARFGEF2* | 10564 | 9.0E-05 | 0.75 | 7.3E-11 | pink | turquoise |
| *STAB1* | 23166 | 9.0E-05 | 0.77 | 9.3E-12 | cyan | yellow |
| *SMAD4* | 4089 | 9.0E-05 | 0.79 | 1.6E-12 | black | red |
| *TKT* | 7086 | 9.0E-05 | 0.70 | 2.9E-09 | grey60 | magenta |
| *ARHGEF10* | 9639 | 9.0E-05 | -0.57 | 8.2E-06 | brown | yellow |
| *PDCL* | 5082 | 9.1E-05 | 0.52 | 5.4E-05 | cyan | NA |
| *AUH* | 549 | 9.1E-05 | 0.82 | 2.7E-14 | brown | turquoise |
| *BCKDHB* | 594 | 9.1E-05 | 0.80 | 3.6E-13 | brown | turquoise |
| *HEBP2* | 23593 | 9.2E-05 | 0.62 | 5.2E-07 | blue | turquoise |
| *LSS* | 4047 | 9.3E-05 | 0.73 | 3.5E-10 | blue | red |
| *CSGALNACT1* | 55790 | 9.3E-05 | -0.61 | 1.0E-06 | blue | turquoise |
| *PAIP2* | 51247 | 9.4E-05 | 0.67 | 3.7E-08 | darkturquoise | green |
| *S100A4* | 6275 | 9.4E-05 | -0.83 | 4.7E-15 | brown | yellow |
| *TRIP4* | 9325 | 9.4E-05 | 0.70 | 3.0E-09 | black | red |
| *PDE8A* | 5151 | 9.5E-05 | 0.60 | 1.3E-06 | pink | turquoise |
| *CTSB* | 1508 | 9.6E-05 | 0.87 | 1.5E-17 | cyan | yellow |
| *DCTN4* | 51164 | 9.6E-05 | 0.83 | 7.1E-15 | black | darkgreen |
| *GOLM1* | 51280 | 9.6E-05 | 0.76 | 3.5E-11 | cyan | yellow |
| *SIGLEC1* | 6614 | 9.6E-05 | 0.76 | 1.9E-11 | cyan | yellow |
| *RMND1* | 55005 | 9.8E-05 | 0.76 | 3.3E-11 | brown | turquoise |
| *SCOC* | 60592 | 9.9E-05 | 0.90 | 1.4E-20 | pink | green |
| *TMSB10* | 9168 | 9.9E-05 | -0.77 | 1.4E-11 | brown | turquoise |
| *NQO1* | 1728 | 1.0E-04 | 0.76 | 2.8E-11 | darkgrey | yellow |
| *TMEM119* | 338773 | 1.0E-04 | -0.61 | 1.1E-06 | black | yellow |
| *MOSC2* | 54996 | 1.0E-04 | 0.93 | 8.9E-24 | brown | turquoise |
| *CCDC109B* | 55013 | 1.0E-04 | 0.79 | 1.0E-12 | cyan | yellow |
| *RHOT1* | 55288 | 1.0E-04 | 0.72 | 6.3E-10 | black | yellow |
| *SLC29A3* | 55315 | 1.0E-04 | 0.78 | 2.2E-12 | cyan | yellow |
| *TXNDC15* | 79770 | 1.0E-04 | 0.71 | 1.8E-09 | cyan | yellow |
| *BTF3L4* | 91408 | 1.0E-04 | 0.71 | 1.4E-09 | pink | turquoise |
| *DCTN6* | 10671 | 1.1E-04 | 0.76 | 1.9E-11 | blue | darkgreen |
| *RBPMS* | 11030 | 1.1E-04 | 0.64 | 1.9E-07 | black | red |
| *GPR146* | 115330 | 1.1E-04 | 0.74 | 2.1E-10 | black | red |
| *FAM122A* | 116224 | 1.1E-04 | 0.78 | 4.2E-12 | blue | turquoise |
| *EXT1* | 2131 | 1.1E-04 | -0.52 | 5.1E-05 | black | yellow |
| *FLNC* | 2318 | 1.1E-04 | -0.72 | 1.1E-09 | brown | turquoise |
| *GCLC* | 2729 | 1.1E-04 | 0.66 | 6.7E-08 | black | green |
| *GUSB* | 2990 | 1.1E-04 | -0.79 | 8.1E-13 | brown | yellow |
| *MEF2D* | 4209 | 1.1E-04 | -0.65 | 8.2E-08 | darkgrey | NA |
| *GPR126* | 57211 | 1.1E-04 | 0.63 | 3.2E-07 | cyan | turquoise |
| *SNAPC3* | 6619 | 1.1E-04 | 0.72 | 1.2E-09 | brown | turquoise |
| *ATAD1* | 84896 | 1.1E-04 | 0.72 | 1.0E-09 | pink | turquoise |
| *CD163* | 9332 | 1.1E-04 | 0.90 | 3.0E-20 | cyan | yellow |
| *ADK* | 132 | 1.2E-04 | 0.56 | 9.5E-06 | grey60 | green |
| *CSNK2A1* | 1457 | 1.2E-04 | 0.81 | 1.6E-13 | black | red |
| *CYBASC3* | 220002 | 1.2E-04 | 0.85 | 3.7E-16 | darkgrey | purple |
| *ALG6* | 29929 | 1.2E-04 | -0.59 | 3.1E-06 | brown | turquoise |
| *LTC4S* | 4056 | 1.2E-04 | 0.62 | 5.7E-07 | cyan | yellow |
| *NDUFA5* | 4698 | 1.2E-04 | 0.71 | 2.6E-09 | pink | turquoise |
| *KLHL24* | 54800 | 1.2E-04 | 0.74 | 2.0E-10 | black | yellow |
| *MFSD1* | 64747 | 1.2E-04 | 0.74 | 1.4E-10 | cyan | yellow |
| *TGFB3* | 7043 | 1.2E-04 | -0.54 | 3.0E-05 | black | royalblue |
| *PTPLA* | 9200 | 1.2E-04 | -0.55 | 1.4E-05 | darkgrey | turquoise |
| *FAM13A* | 10144 | 1.3E-04 | 0.85 | 3.2E-16 | black | red |
| *SMNDC1* | 10285 | 1.3E-04 | 0.73 | 2.8E-10 | black | red |
| *TTL* | 150465 | 1.3E-04 | 0.67 | 2.5E-08 | cyan | yellow |
| *CTSH* | 1512 | 1.3E-04 | -0.85 | 2.1E-16 | blue | yellow |
| *DARS* | 1615 | 1.3E-04 | 0.61 | 1.0E-06 | blue | red |
| *DLD* | 1738 | 1.3E-04 | 0.88 | 1.4E-18 | brown | turquoise |
| *LRRN4CL* | 221091 | 1.3E-04 | 0.58 | 3.5E-06 | cyan | yellow |
| *HPGDS* | 27306 | 1.3E-04 | 0.86 | 1.7E-16 | cyan | yellow |
| *FAM63B* | 54629 | 1.3E-04 | 0.51 | 7.8E-05 | black | darkgreen |
| *AZI2* | 64343 | 1.3E-04 | 0.79 | 9.0E-13 | pink | green |
| *SSBP1* | 6742 | 1.3E-04 | 0.63 | 3.9E-07 | blue | blue |
| *ZFAND1* | 79752 | 1.3E-04 | 0.72 | 7.7E-10 | black | red |
| *YARS* | 8565 | 1.3E-04 | -0.65 | 8.9E-08 | black | yellow |
| *SLIT2* | 9353 | 1.3E-04 | -0.66 | 6.1E-08 | black | red |
| *CDC25B* | 994 | 1.3E-04 | 0.72 | 9.9E-10 | turquoise | turquoise |
| *CDH11* | 1009 | 1.4E-04 | 0.76 | 2.4E-11 | cyan | yellow |
| *SPON2* | 10417 | 1.4E-04 | -0.60 | 1.6E-06 | black | yellow |
| *COX8A* | 1351 | 1.4E-04 | 0.77 | 6.7E-12 | brown | turquoise |
| *CTSZ* | 1522 | 1.4E-04 | 0.51 | 8.8E-05 | cyan | black |
| *CYC1* | 1537 | 1.4E-04 | 0.85 | 5.3E-16 | brown | turquoise |
| *MEGF6* | 1953 | 1.4E-04 | -0.60 | 2.1E-06 | black | turquoise |
| *SH3PXD2B* | 285590 | 1.4E-04 | 0.49 | 1.4E-04 | darkred | royalblue |
| *CNTN3* | 5067 | 1.4E-04 | 0.64 | 1.5E-07 | darkgrey | yellow |
| *PCCA* | 5095 | 1.4E-04 | -0.70 | 3.7E-09 | cyan | turquoise |
| *PFKM* | 5213 | 1.4E-04 | 0.44 | 9.8E-04 | darkred | green |
| *DRAM1* | 55332 | 1.4E-04 | 0.80 | 2.7E-13 | cyan | yellow |
| *GSDMB* | 55876 | 1.4E-04 | 0.81 | 7.4E-14 | black | red |
| *RPLP2* | 6181 | 1.4E-04 | 0.58 | 5.3E-06 | blue | blue |
| *S100A2* | 6273 | 1.4E-04 | -0.56 | 1.3E-05 | black | NA |
| *TSPYL1* | 7259 | 1.4E-04 | 0.82 | 2.7E-14 | pink | green |
| *TYRO3* | 7301 | 1.4E-04 | 0.71 | 1.6E-09 | black | turquoise |
| *TTLL7* | 79739 | 1.4E-04 | 0.81 | 1.7E-13 | black | NA |
| *ENTPD3* | 956 | 1.4E-04 | 0.61 | 1.1E-06 | black | NA |
| *GPNMB* | 10457 | 1.5E-04 | 0.89 | 1.5E-19 | cyan | yellow |
| *APPBP2* | 10513 | 1.5E-04 | 0.78 | 5.0E-12 | black | red |
| *NTAN1* | 123803 | 1.5E-04 | 0.70 | 3.2E-09 | cyan | yellow |
| *CYP27A1* | 1593 | 1.5E-04 | 0.68 | 1.3E-08 | cyan | yellow |
| *LPIN1* | 23175 | 1.5E-04 | 0.67 | 2.3E-08 | black | yellow |
| *CADM2* | 253559 | 1.5E-04 | 0.69 | 9.0E-09 | black | NA |
| *NDUFAB1* | 4706 | 1.5E-04 | 0.83 | 4.5E-15 | brown | turquoise |
| *PLOD2* | 5352 | 1.5E-04 | 0.75 | 7.9E-11 | pink | green |
| *AGPAT5* | 55326 | 1.5E-04 | -0.74 | 1.8E-10 | brown | NA |
| *SLC39A10* | 57181 | 1.5E-04 | 0.53 | 4.2E-05 | cyan | turquoise |
| *RNPEP* | 6051 | 1.5E-04 | -0.51 | 7.2E-05 | blue | turquoise |
| *VPS72* | 6944 | 1.5E-04 | 0.68 | 2.1E-08 | black | red |
| *PLEKHF2* | 79666 | 1.5E-04 | 0.66 | 4.3E-08 | purple | royalblue |
| *SLC2A10* | 81031 | 1.5E-04 | 0.70 | 5.0E-09 | darkred | darkred |
| *TTC36* | 143941 | 1.6E-04 | 0.68 | 1.3E-08 | black | NA |
| *CTSK* | 1513 | 1.6E-04 | 0.82 | 4.2E-14 | cyan | yellow |
| *AEBP1* | 165 | 1.6E-04 | -0.51 | 7.0E-05 | black | yellow |
| *GDF10* | 2662 | 1.6E-04 | 0.60 | 1.8E-06 | darkgrey | yellow |
| *MN1* | 4330 | 1.6E-04 | 0.71 | 1.3E-09 | darkgrey | purple |
| *OXA1L* | 5018 | 1.6E-04 | 0.72 | 7.0E-10 | blue | turquoise |
| *PCCB* | 5096 | 1.6E-04 | 0.78 | 3.0E-12 | brown | turquoise |
| *CLDND1* | 56650 | 1.6E-04 | 0.79 | 1.0E-12 | black | red |
| *GINS3* | 64785 | 1.6E-04 | -0.79 | 8.1E-13 | darkgrey | red |
| *P4HA2* | 8974 | 1.6E-04 | 0.73 | 3.8E-10 | cyan | yellow |
| *MAFB* | 9935 | 1.6E-04 | 0.79 | 8.0E-13 | cyan | yellow |
| *USP16* | 10600 | 1.7E-04 | 0.71 | 2.0E-09 | blue | turquoise |
| *SNRNP27* | 11017 | 1.7E-04 | 0.81 | 1.8E-13 | royalblue | turquoise |
| *FAP* | 2191 | 1.7E-04 | 0.75 | 4.5E-11 | cyan | NA |
| *C11orf45* | 219833 | 1.7E-04 | 0.73 | 4.2E-10 | cyan | NA |
| *FRMD4B* | 23150 | 1.7E-04 | 0.79 | 7.2E-13 | cyan | yellow |
| *ZNF521* | 25925 | 1.7E-04 | 0.73 | 4.6E-10 | darkred | darkred |
| *CFH* | 3075 | 1.7E-04 | -0.64 | 1.9E-07 | black | yellow |
| *HSD11B1* | 3290 | 1.7E-04 | 0.82 | 3.3E-14 | cyan | yellow |
| *ATP1A1* | 476 | 1.7E-04 | -0.73 | 5.6E-10 | midnightblue | turquoise |
| *ACO1* | 48 | 1.7E-04 | 0.65 | 1.0E-07 | black | turquoise |
| *PCBD1* | 5092 | 1.7E-04 | 0.75 | 4.5E-11 | brown | turquoise |
| *SYTL2* | 54843 | 1.7E-04 | -0.59 | 2.7E-06 | brown | turquoise |
| *RIF1* | 55183 | 1.7E-04 | 0.58 | 3.5E-06 | black | red |
| *BRF2* | 55290 | 1.7E-04 | 0.77 | 1.1E-11 | brown | turquoise |
| *TNFAIP3* | 7128 | 1.7E-04 | -0.81 | 1.0E-13 | black | turquoise |
| *SLC25A16* | 8034 | 1.7E-04 | 0.88 | 7.7E-19 | brown | turquoise |
| *HDHD3* | 81932 | 1.7E-04 | 0.74 | 1.1E-10 | midnightblue | magenta |
| *NRIP1* | 8204 | 1.7E-04 | 0.75 | 4.5E-11 | black | red |
| *CRIPT* | 9419 | 1.7E-04 | 0.79 | 1.6E-12 | pink | green |
| *MIER3* | 166968 | 1.8E-04 | 0.80 | 4.7E-13 | purple | NA |
| *ACADSB* | 36 | 1.8E-04 | -0.69 | 5.6E-09 | cyan | turquoise |
| *NFX1* | 4799 | 1.8E-04 | 0.82 | 2.7E-14 | black | red |
| *C14orf129* | 51527 | 1.8E-04 | -0.62 | 5.8E-07 | brown | turquoise |
| *PEX7* | 5191 | 1.8E-04 | 0.75 | 8.1E-11 | royalblue | turquoise |
| *HCFC1R1* | 54985 | 1.8E-04 | -0.45 | 7.4E-04 | black | royalblue |
| *PTGFR* | 5737 | 1.8E-04 | 0.73 | 4.9E-10 | cyan | yellow |
| *SLC1A5* | 6510 | 1.8E-04 | 0.69 | 6.0E-09 | brown | turquoise |
| *UBE2D3* | 7323 | 1.8E-04 | 0.47 | 3.2E-04 | black | darkgreen |
| *DHRS3* | 9249 | 1.8E-04 | 0.85 | 4.1E-16 | brown | turquoise |
| *AIMP1* | 9255 | 1.8E-04 | 0.84 | 3.2E-15 | pink | green |
| *RB1CC1* | 9821 | 1.8E-04 | 0.74 | 2.5E-10 | black | darkgrey |
| *HEXIM1* | 10614 | 1.9E-04 | -0.75 | 4.7E-11 | black | purple |
| *MAP3K5* | 4217 | 1.9E-04 | 0.78 | 2.3E-12 | black | purple |
| *PNPLA8* | 50640 | 1.9E-04 | 0.65 | 8.4E-08 | black | darkgrey |
| *LGMN* | 5641 | 1.9E-04 | 0.84 | 3.0E-15 | cyan | yellow |
| *RPN2* | 6185 | 1.9E-04 | -0.80 | 5.8E-13 | black | purple |
| *PTP4A2* | 8073 | 1.9E-04 | 0.37 | 6.6E-03 | black | turquoise |
| *CD47* | 961 | 1.9E-04 | -0.76 | 1.8E-11 | blue | turquoise |
| *B3GALTL* | 145173 | 2.0E-04 | 0.57 | 8.1E-06 | darkred | darkred |
| *CHSY1* | 22856 | 2.0E-04 | -0.82 | 3.4E-14 | brown | turquoise |
| *LETMD1* | 25875 | 2.0E-04 | 0.84 | 1.5E-15 | black | red |
| *SLC40A1* | 30061 | 2.0E-04 | 0.67 | 2.9E-08 | cyan | yellow |
| *HEXB* | 3074 | 2.0E-04 | 0.84 | 1.4E-15 | cyan | yellow |
| *NAT8L* | 339983 | 2.0E-04 | 0.87 | 2.7E-17 | brown | turquoise |
| *C4orf48* | 401115 | 2.0E-04 | -0.60 | 2.0E-06 | black | yellow |
| *MMP2* | 4313 | 2.0E-04 | 0.73 | 2.9E-10 | darkred | royalblue |
| *RNFT1* | 51136 | 2.0E-04 | 0.83 | 4.5E-15 | pink | green |
| *LRRC1* | 55227 | 2.0E-04 | -0.72 | 5.9E-10 | brown | turquoise |
| *TMEM127* | 55654 | 2.0E-04 | -0.80 | 5.1E-13 | black | yellow |
| *MUDENG* | 55745 | 2.0E-04 | 0.84 | 1.4E-15 | royalblue | turquoise |
| *AVPI1* | 60370 | 2.0E-04 | 0.78 | 2.8E-12 | brown | turquoise |
| *CDKN2AIPNL* | 91368 | 2.0E-04 | 0.65 | 1.2E-07 | pink | turquoise |
| *C1D* | 10438 | 2.1E-04 | 0.46 | 4.0E-04 | blue | turquoise |
| *C6orf192* | 116843 | 2.1E-04 | -0.71 | 1.4E-09 | blue | yellow |
| *ADH1B* | 125 | 2.1E-04 | 0.89 | 9.8E-20 | black | red |
| *MMAA* | 166785 | 2.1E-04 | 0.69 | 1.1E-08 | blue | blue |
| *GPR37* | 2861 | 2.1E-04 | 0.56 | 1.2E-05 | blue | NA |
| *CAPRIN1* | 4076 | 2.1E-04 | 0.82 | 1.8E-14 | pink | darkgreen |
| *ACO2* | 50 | 2.1E-04 | 0.91 | 7.9E-22 | brown | turquoise |
| *MS4A4A* | 51338 | 2.1E-04 | 0.89 | 8.7E-20 | cyan | yellow |
| *RNASE4* | 6038 | 2.1E-04 | 0.86 | 1.6E-16 | black | red |
| *XBP1* | 7494 | 2.1E-04 | -0.48 | 2.2E-04 | darkgrey | green |
| *GTF3C3* | 9330 | 2.1E-04 | 0.79 | 1.2E-12 | black | red |
| *TSPAN3* | 10099 | 2.2E-04 | 0.86 | 1.0E-16 | brown | turquoise |
| *PECI* | 10455 | 2.2E-04 | 0.81 | 1.2E-13 | blue | turquoise |
| *ASB8* | 140461 | 2.2E-04 | -0.42 | 1.5E-03 | cyan | turquoise |
| *NT5DC1* | 221294 | 2.2E-04 | 0.66 | 7.4E-08 | blue | turquoise |
| *HEY2* | 23493 | 2.2E-04 | -0.66 | 4.8E-08 | darkgrey | NA |
| *NDUFV3* | 4731 | 2.2E-04 | 0.69 | 8.0E-09 | blue | lightcyan |
| *PIGP* | 51227 | 2.2E-04 | -0.65 | 1.3E-07 | turquoise | turquoise |
| *SLAMF8* | 56833 | 2.2E-04 | 0.85 | 2.7E-16 | cyan | yellow |
| *KIAA1598* | 57698 | 2.2E-04 | 0.83 | 4.9E-15 | cyan | yellow |
| *TANK* | 10010 | 2.3E-04 | 0.73 | 5.0E-10 | pink | darkgreen |
| *ABHD15* | 116236 | 2.3E-04 | 0.81 | 2.2E-13 | brown | turquoise |
| *ARHGEF26* | 26084 | 2.3E-04 | 0.79 | 1.5E-12 | black | red |
| *FBXO3* | 26273 | 2.3E-04 | 0.75 | 4.6E-11 | black | darkgrey |
| *MGP* | 4256 | 2.3E-04 | 0.68 | 1.2E-08 | cyan | yellow |
| *PENK* | 5179 | 2.3E-04 | 0.71 | 1.6E-09 | cyan | yellow |
| *C1QB* | 713 | 2.3E-04 | 0.87 | 8.8E-18 | cyan | yellow |
| *IRS2* | 8660 | 2.3E-04 | 0.69 | 6.2E-09 | black | yellow |
| *ADIPOQ* | 9370 | 2.3E-04 | -0.69 | 1.0E-08 | cyan | yellow |
| *CDKN1A* | 1026 | 2.4E-04 | 0.60 | 1.6E-06 | cyan | turquoise |
| *ECHS1* | 1892 | 2.4E-04 | 0.89 | 1.8E-19 | brown | turquoise |
| *PATL1* | 219988 | 2.4E-04 | 0.60 | 1.6E-06 | darkturquoise | NA |
| *ERLEC1* | 27248 | 2.4E-04 | 0.56 | 8.9E-06 | cyan | yellow |
| *BLNK* | 29760 | 2.4E-04 | -0.85 | 2.3E-16 | blue | yellow |
| *FAM73A* | 374986 | 2.4E-04 | 0.70 | 3.4E-09 | pink | darkgreen |
| *MMP19* | 4327 | 2.4E-04 | 0.71 | 2.5E-09 | cyan | yellow |
| *ACP5* | 54 | 2.4E-04 | 0.87 | 3.0E-17 | cyan | yellow |
| *BCAP29* | 55973 | 2.4E-04 | 0.67 | 3.9E-08 | blue | turquoise |
| *CFB* | 629 | 2.4E-04 | 0.80 | 3.1E-13 | cyan | yellow |
| *MICALL2* | 79778 | 2.4E-04 | -0.72 | 1.2E-09 | brown | turquoise |
| *AASS* | 10157 | 2.5E-04 | 0.84 | 3.4E-15 | black | red |
| *VASN* | 114990 | 2.5E-04 | 0.56 | 1.2E-05 | cyan | yellow |
| *GGT5* | 2687 | 2.5E-04 | -0.58 | 5.0E-06 | brown | turquoise |
| *GRTP1* | 79774 | 2.5E-04 | 0.64 | 2.0E-07 | blue | red |
| *AKAP1* | 8165 | 2.5E-04 | 0.75 | 8.7E-11 | black | turquoise |
| *POLR3GL* | 84265 | 2.5E-04 | 0.64 | 2.0E-07 | blue | red |
| *CHN1* | 1123 | 2.6E-04 | -0.62 | 7.1E-07 | brown | turquoise |
| *ZNF787* | 126208 | 2.6E-04 | -0.56 | 9.4E-06 | darkgrey | turquoise |
| *C19orf28* | 126321 | 2.6E-04 | 0.76 | 3.7E-11 | cyan | NA |
| *TMEM192* | 201931 | 2.6E-04 | 0.77 | 6.6E-12 | pink | turquoise |
| *GALC* | 2581 | 2.6E-04 | 0.72 | 6.4E-10 | cyan | turquoise |
| *TPK1* | 27010 | 2.6E-04 | -0.84 | 2.1E-15 | blue | yellow |
| *PPA2* | 27068 | 2.6E-04 | 0.86 | 1.9E-16 | royalblue | turquoise |
| *MFAP4* | 4239 | 2.6E-04 | 0.70 | 3.2E-09 | cyan | yellow |
| *UQCC* | 55245 | 2.6E-04 | 0.83 | 1.3E-14 | midnightblue | turquoise |
| *BCAT1* | 586 | 2.6E-04 | 0.89 | 3.0E-19 | cyan | yellow |
| *FAT3* | 120114 | 2.7E-04 | 0.76 | 4.0E-11 | brown | turquoise |
| *DDX41* | 51428 | 2.7E-04 | -0.54 | 2.4E-05 | black | yellow |
| *DUSP23* | 54935 | 2.7E-04 | -0.41 | 2.2E-03 | black | yellow |
| *C9orf6* | 54942 | 2.7E-04 | 0.62 | 7.3E-07 | blue | turquoise |
| *SOBP* | 55084 | 2.7E-04 | 0.62 | 6.1E-07 | darkgrey | NA |
| *PXMP2* | 5827 | 2.7E-04 | 0.86 | 1.0E-16 | brown | turquoise |
| *SSB* | 6741 | 2.7E-04 | 0.72 | 6.6E-10 | pink | darkgreen |
| *PHLDA2* | 7262 | 2.7E-04 | -0.81 | 1.1E-13 | black | yellow |
| *GPHN* | 10243 | 2.8E-04 | 0.78 | 2.2E-12 | black | yellow |
| *DLG1* | 1739 | 2.8E-04 | 0.64 | 1.6E-07 | blue | green |
| *IAH1* | 285148 | 2.8E-04 | 0.62 | 6.6E-07 | blue | blue |
| *GPT* | 2875 | 2.8E-04 | 0.66 | 7.1E-08 | brown | turquoise |
| *PHOSPHO2* | 493911 | 2.8E-04 | 0.39 | 3.4E-03 | blue | turquoise |
| *POLR2I* | 5438 | 2.8E-04 | 0.69 | 6.0E-09 | blue | turquoise |
| *DHTKD1* | 55526 | 2.8E-04 | 0.82 | 3.0E-14 | brown | turquoise |
| *ANKRA2* | 57763 | 2.8E-04 | -0.71 | 1.6E-09 | darkgrey | purple |
| *SLC25A1* | 6576 | 2.8E-04 | 0.81 | 2.1E-13 | midnightblue | turquoise |
| *WDR77* | 79084 | 2.8E-04 | 0.65 | 8.0E-08 | brown | turquoise |
| *KIAA1737* | 85457 | 2.8E-04 | 0.84 | 2.7E-15 | black | red |
| *FBN1* | 2200 | 2.9E-04 | 0.65 | 1.3E-07 | cyan | yellow |
| *SIRT1* | 23411 | 2.9E-04 | 0.82 | 2.0E-14 | black | red |
| *GCHFR* | 2644 | 2.9E-04 | 0.58 | 5.0E-06 | brown | turquoise |
| *NENF* | 29937 | 2.9E-04 | -0.62 | 6.0E-07 | brown | royalblue |
| *PFKP* | 5214 | 2.9E-04 | -0.64 | 2.5E-07 | pink | turquoise |
| *TPM2* | 7169 | 2.9E-04 | -0.66 | 6.5E-08 | black | turquoise |
| *C1S* | 716 | 2.9E-04 | 0.85 | 4.5E-16 | cyan | yellow |
| *SCRN1* | 9805 | 2.9E-04 | 0.70 | 3.3E-09 | cyan | yellow |
| *MED6* | 10001 | 3.0E-04 | 0.58 | 5.4E-06 | pink | green |
| *NAALADL1* | 10004 | 3.0E-04 | -0.41 | 2.4E-03 | darkturquoise | NA |
| *MRPS31* | 10240 | 3.0E-04 | 0.75 | 6.0E-11 | black | turquoise |
| *DAB2* | 1601 | 3.0E-04 | 0.81 | 1.1E-13 | cyan | yellow |
| *FAM171A1* | 221061 | 3.0E-04 | 0.67 | 2.2E-08 | darkgrey | red |
| *ANKRD28* | 23243 | 3.0E-04 | -0.82 | 4.1E-14 | brown | turquoise |
| *ANXA11* | 311 | 3.0E-04 | -0.71 | 1.7E-09 | black | royalblue |
| *PHYH* | 5264 | 3.0E-04 | 0.80 | 3.7E-13 | black | turquoise |
| *YIPF4* | 84272 | 3.0E-04 | 0.81 | 7.7E-14 | pink | green |
| *TP53I3* | 9540 | 3.0E-04 | -0.49 | 1.7E-04 | black | yellow |
| *GNE* | 10020 | 3.1E-04 | 0.81 | 1.4E-13 | black | turquoise |
| *PNRC1* | 10957 | 3.1E-04 | 0.52 | 4.6E-05 | black | red |
| *VPS8* | 23355 | 3.1E-04 | -0.44 | 7.7E-04 | pink | green |
| *C10orf116* | 10974 | 3.2E-04 | 0.73 | 5.0E-10 | blue | turquoise |
| *SPATS2L* | 26010 | 3.2E-04 | -0.69 | 8.7E-09 | turquoise | blue |
| *PC* | 5091 | 3.2E-04 | 0.83 | 5.1E-15 | brown | turquoise |
| *GGCT* | 79017 | 3.2E-04 | 0.65 | 1.1E-07 | black | turquoise |
| *SETD6* | 79918 | 3.2E-04 | -0.49 | 1.9E-04 | turquoise | turquoise |
| *DNAJA3* | 9093 | 3.2E-04 | 0.80 | 6.9E-13 | brown | turquoise |
| *PTDSS1* | 9791 | 3.2E-04 | 0.72 | 1.2E-09 | cyan | yellow |
| *TOMM20* | 9804 | 3.2E-04 | 0.68 | 1.6E-08 | black | turquoise |
| *FDFT1* | 2222 | 3.3E-04 | 0.79 | 1.9E-12 | black | red |
| *SLCO4A1* | 28231 | 3.3E-04 | -0.43 | 1.2E-03 | black | green |
| *MYO1E* | 4643 | 3.3E-04 | 0.63 | 4.0E-07 | cyan | NA |
| *CHPF2* | 54480 | 3.3E-04 | -0.73 | 4.7E-10 | pink | turquoise |
| *MS4A6A* | 64231 | 3.3E-04 | 0.78 | 3.3E-12 | cyan | yellow |
| *MPDZ* | 8777 | 3.3E-04 | 0.77 | 9.6E-12 | black | yellow |
| *PTTG1* | 9232 | 3.3E-04 | -0.69 | 6.8E-09 | brown | NA |
| *CD302* | 9936 | 3.3E-04 | 0.80 | 4.2E-13 | black | red |
| *F13A1* | 2162 | 3.4E-04 | 0.76 | 3.7E-11 | cyan | yellow |
| *GPI* | 2821 | 3.4E-04 | 0.89 | 6.1E-19 | brown | turquoise |
| *ING1* | 3621 | 3.4E-04 | 0.73 | 3.0E-10 | black | red |
| *RPL30* | 6156 | 3.4E-04 | 0.69 | 8.7E-09 | black | red |
| *RNF41* | 10193 | 3.5E-04 | 0.72 | 9.4E-10 | blue | turquoise |
| *TMEM200A* | 114801 | 3.5E-04 | 0.64 | 2.2E-07 | cyan | yellow |
| *KIAA0776* | 23376 | 3.5E-04 | -0.55 | 1.6E-05 | turquoise | darkgrey |
| *RCHY1* | 25898 | 3.5E-04 | -0.61 | 7.6E-07 | green | green |
| *RGMB* | 285704 | 3.5E-04 | -0.62 | 4.7E-07 | darkgrey | red |
| *SPARC* | 6678 | 3.5E-04 | -0.65 | 9.3E-08 | black | turquoise |
| *TRAF5* | 7188 | 3.5E-04 | -0.67 | 2.4E-08 | blue | turquoise |
| *TATDN2* | 9797 | 3.5E-04 | -0.56 | 9.4E-06 | black | turquoise |
| *LRRC32* | 2615 | 3.6E-04 | -0.41 | 2.1E-03 | black | green |
| *NMD3* | 51068 | 3.6E-04 | 0.65 | 8.0E-08 | black | darkgreen |
| *SH3GLB1* | 51100 | 3.6E-04 | 0.66 | 6.7E-08 | midnightblue | turquoise |
| *TIAM1* | 7074 | 3.6E-04 | -0.87 | 6.3E-18 | blue | turquoise |
| *C1QC* | 714 | 3.6E-04 | 0.88 | 3.0E-18 | cyan | yellow |
| *YWHAZ* | 7534 | 3.6E-04 | 0.28 | 3.8E-02 | black | red |
| *SPON1* | 10418 | 3.7E-04 | -0.63 | 4.4E-07 | darkgrey | red |
| *DCXR* | 51181 | 3.7E-04 | 0.76 | 2.4E-11 | blue | turquoise |
| *PDIA4* | 9601 | 3.7E-04 | 0.72 | 1.1E-09 | cyan | yellow |
| *NPC2* | 10577 | 3.8E-04 | 0.91 | 1.5E-21 | cyan | yellow |
| *ACLY* | 47 | 3.8E-04 | 0.81 | 1.7E-13 | royalblue | turquoise |
| *CISD2* | 493856 | 3.8E-04 | 0.56 | 9.2E-06 | cyan | yellow |
| *OLFML3* | 56944 | 3.8E-04 | 0.64 | 1.8E-07 | cyan | yellow |
| *SFT2D1* | 113402 | 3.9E-04 | 0.73 | 5.0E-10 | royalblue | turquoise |
| *SERPINA3* | 12 | 3.9E-04 | -0.65 | 9.0E-08 | brown | NA |
| *APEX1* | 328 | 3.9E-04 | 0.59 | 2.4E-06 | brown | lightcyan |
| *LPL* | 4023 | 3.9E-04 | 0.82 | 3.0E-14 | brown | turquoise |
| *ATP5O* | 539 | 3.9E-04 | 0.73 | 2.5E-10 | brown | turquoise |
| *VEZT* | 55591 | 3.9E-04 | 0.74 | 1.7E-10 | pink | darkgrey |
| *SFRP4* | 6424 | 3.9E-04 | 0.78 | 4.0E-12 | darkgrey | yellow |
| *TMTC1* | 83857 | 3.9E-04 | 0.64 | 1.6E-07 | brown | turquoise |
| *KIAA1012* | 22878 | 4.0E-04 | 0.52 | 5.2E-05 | black | turquoise |
| *PALLD* | 23022 | 4.0E-04 | 0.81 | 6.6E-14 | darkgrey | red |
| *PHC3* | 80012 | 4.0E-04 | 0.79 | 7.9E-13 | purple | red |
| *TXNDC5* | 81567 | 4.0E-04 | 0.63 | 2.6E-07 | cyan | yellow |
| *EGR2* | 1959 | 4.1E-04 | 0.84 | 1.6E-15 | cyan | yellow |
| *CCDC59* | 29080 | 4.1E-04 | 0.78 | 3.8E-12 | black | red |
| *ACACB* | 32 | 4.1E-04 | 0.79 | 8.5E-13 | brown | yellow |
| *ARCN1* | 372 | 4.1E-04 | 0.53 | 3.4E-05 | blue | turquoise |
| *LAMP1* | 3916 | 4.1E-04 | 0.70 | 3.2E-09 | darkgrey | yellow |
| *ARMC1* | 55156 | 4.1E-04 | 0.49 | 1.6E-04 | blue | turquoise |
| *RGS3* | 5998 | 4.1E-04 | 0.73 | 5.5E-10 | brown | yellow |
| *CILP* | 8483 | 4.1E-04 | 0.62 | 7.1E-07 | darkred | yellow |
| *LGALS12* | 85329 | 4.1E-04 | 0.77 | 8.2E-12 | brown | turquoise |
| *PPP1R14A* | 94274 | 4.1E-04 | 0.58 | 4.0E-06 | blue | red |
| *GNPDA1* | 10007 | 4.2E-04 | 0.83 | 5.3E-15 | cyan | yellow |
| *EXD2* | 55218 | 4.2E-04 | 0.70 | 5.4E-09 | brown | turquoise |
| *TOMM22* | 56993 | 4.2E-04 | 0.66 | 5.4E-08 | brown | turquoise |
| *MRPL9* | 65005 | 4.2E-04 | -0.65 | 1.2E-07 | turquoise | blue |
| *SYPL1* | 6856 | 4.2E-04 | 0.76 | 3.0E-11 | pink | green |
| *C6orf211* | 79624 | 4.2E-04 | 0.89 | 5.0E-19 | pink | turquoise |
| *TOX4* | 9878 | 4.2E-04 | 0.61 | 8.6E-07 | black | red |
| *ARL6IP1* | 23204 | 4.3E-04 | 0.71 | 2.5E-09 | black | yellow |
| *DHRS9* | 10170 | 4.4E-04 | 0.84 | 9.8E-16 | cyan | yellow |
| *HRSP12* | 10247 | 4.4E-04 | 0.82 | 2.6E-14 | brown | turquoise |
| *TOMM34* | 10953 | 4.4E-04 | 0.54 | 2.1E-05 | cyan | NA |
| *C1orf85* | 112770 | 4.4E-04 | 0.71 | 2.7E-09 | cyan | yellow |
| *ALDH2* | 217 | 4.4E-04 | 0.69 | 8.0E-09 | black | red |
| *NEDD4L* | 23327 | 4.4E-04 | 0.80 | 5.7E-13 | black | red |
| *MRPS18B* | 28973 | 4.4E-04 | 0.67 | 2.3E-08 | lightyellow | turquoise |
| *TGOLN2* | 10618 | 4.5E-04 | -0.64 | 2.0E-07 | black | yellow |
| *CMTM8* | 152189 | 4.5E-04 | 0.64 | 1.6E-07 | black | turquoise |
| *AGL* | 178 | 4.5E-04 | 0.65 | 8.5E-08 | pink | turquoise |
| *KIAA0564* | 23078 | 4.5E-04 | 0.81 | 1.2E-13 | brown | turquoise |
| *FTH1* | 2495 | 4.5E-04 | 0.70 | 4.7E-09 | cyan | yellow |
| *JAK2* | 3717 | 4.5E-04 | 0.81 | 9.1E-14 | purple | red |
| *PEX19* | 5824 | 4.5E-04 | 0.84 | 3.9E-15 | brown | turquoise |
| *UBE3A* | 7337 | 4.5E-04 | 0.81 | 6.8E-14 | black | yellow |
| *CEP63* | 80254 | 4.5E-04 | 0.64 | 2.0E-07 | pink | turquoise |
| *LONP2* | 83752 | 4.5E-04 | 0.89 | 7.7E-20 | brown | turquoise |
| *FBLN2* | 2199 | 4.6E-04 | 0.55 | 1.5E-05 | cyan | darkred |
| *SPOPL* | 339745 | 4.6E-04 | -0.50 | 9.9E-05 | green | royalblue |
| *ASAP3* | 55616 | 4.6E-04 | -0.60 | 1.9E-06 | brown | royalblue |
| *PTGFRN* | 5738 | 4.6E-04 | 0.68 | 1.8E-08 | cyan | yellow |
| *GALNT11* | 63917 | 4.6E-04 | -0.33 | 1.5E-02 | black | yellow |
| *COL21A1* | 81578 | 4.6E-04 | -0.49 | 2.0E-04 | brown | turquoise |
| *LZTS2* | 84445 | 4.6E-04 | 0.68 | 1.7E-08 | blue | turquoise |
| *MAN2B2* | 23324 | 4.7E-04 | -0.67 | 2.7E-08 | brown | yellow |
| *ANKDD1A* | 348094 | 4.7E-04 | 0.74 | 1.9E-10 | darkgrey | turquoise |
| *SEMA3G* | 56920 | 4.7E-04 | 0.63 | 3.9E-07 | cyan | turquoise |
| *NEK4* | 6787 | 4.7E-04 | 0.71 | 1.5E-09 | purple | darkgrey |
| *LRRC41* | 10489 | 4.8E-04 | 0.75 | 9.2E-11 | brown | turquoise |
| *C5orf35* | 133383 | 4.8E-04 | 0.62 | 5.3E-07 | pink | turquoise |
| *HNRNPF* | 3185 | 4.8E-04 | 0.52 | 5.0E-05 | purple | red |
| *NSF* | 4905 | 4.8E-04 | -0.68 | 1.3E-08 | black | yellow |
| *PTPN3* | 5774 | 4.8E-04 | 0.80 | 5.0E-13 | blue | yellow |
| *TTC35* | 9694 | 4.8E-04 | 0.78 | 4.1E-12 | pink | green |
| *AHNAK2* | 113146 | 4.9E-04 | 0.63 | 3.9E-07 | darkred | NA |
| *LHX6* | 26468 | 4.9E-04 | -0.39 | 3.3E-03 | brown | NA |
| *CLMN* | 79789 | 4.9E-04 | 0.81 | 1.3E-13 | brown | turquoise |
| *ARIH1* | 25820 | 5.0E-04 | 0.78 | 2.9E-12 | black | red |
| *MAN2A1* | 4124 | 5.0E-04 | -0.68 | 2.0E-08 | blue | yellow |
| *SLC15A3* | 51296 | 5.0E-04 | 0.72 | 6.4E-10 | cyan | yellow |
| *C1orf103* | 55791 | 5.0E-04 | 0.48 | 2.4E-04 | pink | green |
| *RBP1* | 5947 | 5.0E-04 | -0.56 | 9.6E-06 | turquoise | turquoise |
| *C18orf45* | 85019 | 5.0E-04 | -0.54 | 2.0E-05 | brown | green |
| *TBC1D16* | 125058 | 5.1E-04 | -0.52 | 4.7E-05 | black | NA |
| *FOLR2* | 2350 | 5.1E-04 | 0.88 | 1.4E-18 | cyan | yellow |
| *APC* | 324 | 5.1E-04 | 0.47 | 3.5E-04 | black | NA |
| *PSMD12* | 5718 | 5.1E-04 | 0.75 | 7.1E-11 | pink | green |
| *TPD52L1* | 7164 | 5.1E-04 | 0.79 | 1.2E-12 | brown | yellow |
| *METTL5* | 29081 | 5.2E-04 | -0.59 | 3.2E-06 | turquoise | turquoise |
| *AKAP11* | 11215 | 5.3E-04 | 0.81 | 7.3E-14 | purple | red |
| *EIF4EBP1* | 1978 | 5.3E-04 | -0.63 | 4.3E-07 | darkgrey | red |
| *ABCC1* | 4363 | 5.3E-04 | 0.71 | 1.3E-09 | cyan | yellow |
| *SAR1B* | 51128 | 5.3E-04 | 0.86 | 1.2E-16 | pink | green |
| *FKBP11* | 51303 | 5.3E-04 | -0.78 | 4.7E-12 | blue | yellow |
| *CHMP5* | 51510 | 5.3E-04 | 0.77 | 8.7E-12 | pink | green |
| *DOLPP1* | 57171 | 5.3E-04 | 0.41 | 1.9E-03 | black | turquoise |
| *HS3ST2* | 9956 | 5.3E-04 | -0.73 | 3.8E-10 | brown | NA |
| *CTH* | 1491 | 5.4E-04 | 0.79 | 8.7E-13 | black | red |
| *MTIF3* | 219402 | 5.4E-04 | 0.73 | 4.3E-10 | blue | red |
| *CHMP2B* | 25978 | 5.4E-04 | 0.79 | 1.3E-12 | pink | green |
| *INHBB* | 3625 | 5.4E-04 | 0.65 | 8.2E-08 | darkgrey | yellow |
| *ZYG11B* | 79699 | 5.4E-04 | 0.69 | 1.1E-08 | brown | turquoise |
| *LOXL3* | 84695 | 5.4E-04 | -0.58 | 4.1E-06 | brown | turquoise |
| *ARID5A* | 10865 | 5.5E-04 | -0.67 | 2.6E-08 | pink | turquoise |
| *C11orf1* | 64776 | 5.5E-04 | 0.80 | 5.5E-13 | brown | turquoise |
| *MAP3K7* | 6885 | 5.5E-04 | 0.64 | 1.8E-07 | purple | darkgreen |
| *PPP1R16A* | 84988 | 5.5E-04 | 0.75 | 5.9E-11 | brown | yellow |
| *DMRT2* | 10655 | 5.6E-04 | 0.80 | 2.8E-13 | black | red |
| *CRHBP* | 1393 | 5.6E-04 | -0.63 | 3.4E-07 | cyan | turquoise |
| *ALDOC* | 230 | 5.6E-04 | 0.72 | 1.0E-09 | brown | turquoise |
| *UCHL5* | 51377 | 5.6E-04 | 0.84 | 1.8E-15 | pink | green |
| *TPT1* | 7178 | 5.6E-04 | 0.51 | 7.6E-05 | black | darkgreen |
| *HIGD2A* | 192286 | 5.7E-04 | 0.79 | 7.3E-13 | brown | turquoise |
| *HMGB2* | 3148 | 5.7E-04 | -0.78 | 3.1E-12 | darkgrey | purple |
| *ADCK1* | 57143 | 5.7E-04 | -0.54 | 3.1E-05 | purple | purple |
| *DRG1* | 4733 | 5.8E-04 | 0.78 | 4.9E-12 | blue | turquoise |
| *NAA20* | 51126 | 5.8E-04 | -0.70 | 3.9E-09 | turquoise | blue |
| *TRMT5* | 57570 | 5.8E-04 | 0.66 | 4.5E-08 | black | turquoise |
| *COL3A1* | 1281 | 5.9E-04 | -0.61 | 1.0E-06 | black | yellow |
| *DDX59* | 83479 | 5.9E-04 | 0.67 | 3.5E-08 | black | red |
| *VAMP3* | 9341 | 6.0E-04 | 0.73 | 4.9E-10 | darkturquoise | green |
| *KCTD6* | 200845 | 6.1E-04 | 0.77 | 9.3E-12 | black | red |
| *KIAA0090* | 23065 | 6.1E-04 | 0.56 | 8.9E-06 | cyan | yellow |
| *GTF2E2* | 2961 | 6.1E-04 | 0.78 | 3.6E-12 | brown | turquoise |
| *IFI16* | 3428 | 6.1E-04 | 0.69 | 6.7E-09 | cyan | turquoise |
| *TMEM85* | 51234 | 6.1E-04 | 0.72 | 6.2E-10 | pink | green |
| *TMEM206* | 55248 | 6.1E-04 | -0.79 | 7.2E-13 | blue | yellow |
| *TRAM2* | 9697 | 6.1E-04 | 0.81 | 9.6E-14 | cyan | yellow |
| *PLIN3* | 10226 | 6.2E-04 | -0.72 | 7.1E-10 | black | yellow |
| *UBE4B* | 10277 | 6.2E-04 | 0.60 | 2.0E-06 | black | darkgreen |
| *PLEKHO2* | 80301 | 6.2E-04 | -0.82 | 1.8E-14 | black | yellow |
| *MADD* | 8567 | 6.2E-04 | -0.71 | 1.6E-09 | pink | turquoise |
| *FIG4* | 9896 | 6.2E-04 | -0.37 | 6.1E-03 | brown | turquoise |
| *C2orf49* | 79074 | 6.3E-04 | 0.60 | 1.8E-06 | black | turquoise |
| *METAP2* | 10988 | 6.4E-04 | 0.78 | 3.5E-12 | pink | blue |
| *FOXP1* | 27086 | 6.4E-04 | -0.60 | 1.6E-06 | blue | turquoise |
| *ANAPC4* | 29945 | 6.4E-04 | -0.67 | 3.9E-08 | brown | magenta |
| *MRPS36* | 92259 | 6.4E-04 | 0.81 | 6.7E-14 | pink | turquoise |
| *NOL7* | 51406 | 6.5E-04 | 0.48 | 2.0E-04 | black | darkgreen |
| *SEC31A* | 22872 | 6.6E-04 | -0.63 | 2.6E-07 | brown | royalblue |
| *DISP1* | 84976 | 6.6E-04 | 0.70 | 3.9E-09 | cyan | darkred |
| *NUDT6* | 11162 | 6.7E-04 | 0.79 | 1.4E-12 | blue | turquoise |
| *KCNIP2* | 30819 | 6.7E-04 | 0.66 | 7.7E-08 | brown | turquoise |
| *MRPS22* | 56945 | 6.7E-04 | 0.78 | 3.7E-12 | blue | turquoise |
| *GCDH* | 2639 | 6.8E-04 | 0.79 | 1.3E-12 | brown | turquoise |
| *ADHFE1* | 137872 | 6.9E-04 | -0.70 | 4.5E-09 | cyan | yellow |
| *PDHB* | 5162 | 6.9E-04 | 0.80 | 3.9E-13 | royalblue | turquoise |
| *BDKRB2* | 624 | 6.9E-04 | 0.53 | 4.1E-05 | darkred | yellow |
| *HYI* | 81888 | 6.9E-04 | 0.74 | 1.6E-10 | blue | yellow |
| *CD14* | 929 | 6.9E-04 | -0.80 | 6.6E-13 | blue | yellow |
| *LRBA* | 987 | 6.9E-04 | 0.46 | 5.2E-04 | darkgrey | turquoise |
| *FCGRT* | 2217 | 7.0E-04 | 0.69 | 7.8E-09 | cyan | yellow |
| *C14orf106* | 55320 | 7.0E-04 | 0.39 | 3.2E-03 | black | red |
| *FBXO11* | 80204 | 7.0E-04 | 0.81 | 1.4E-13 | black | red |
| *COL6A3* | 1293 | 7.1E-04 | 0.78 | 4.6E-12 | cyan | yellow |
| *CLDN15* | 24146 | 7.1E-04 | -0.57 | 8.4E-06 | brown | turquoise |
| *MYLK* | 4638 | 7.1E-04 | 0.69 | 1.0E-08 | darkgrey | yellow |
| *C18orf55* | 29090 | 7.2E-04 | 0.81 | 1.6E-13 | pink | turquoise |
| *ATL1* | 51062 | 7.2E-04 | 0.74 | 1.4E-10 | darkred | darkred |
| *MRPL4* | 51073 | 7.2E-04 | 0.85 | 3.3E-16 | midnightblue | turquoise |
| *HPS6* | 79803 | 7.2E-04 | 0.65 | 1.0E-07 | blue | green |
| *MTMR11* | 10903 | 7.3E-04 | -0.66 | 6.0E-08 | blue | NA |
| *PDE1A* | 5136 | 7.3E-04 | 0.62 | 5.2E-07 | cyan | yellow |
| *EIF3M* | 10480 | 7.4E-04 | 0.58 | 4.5E-06 | black | darkgrey |
| *NIPSNAP3A* | 25934 | 7.4E-04 | 0.63 | 2.9E-07 | blue | turquoise |
| *KIT* | 3815 | 7.4E-04 | 0.58 | 4.2E-06 | cyan | yellow |
| *TBX15* | 6913 | 7.4E-04 | 0.78 | 5.3E-12 | black | red |
| *PSMD6* | 9861 | 7.4E-04 | 0.45 | 6.8E-04 | brown | green |
| *ABCA1* | 19 | 7.5E-04 | 0.54 | 2.4E-05 | pink | turquoise |
| *CENPK* | 64105 | 7.5E-04 | -0.72 | 6.4E-10 | blue | turquoise |
| *LPCAT1* | 79888 | 7.5E-04 | 0.71 | 2.5E-09 | turquoise | turquoise |
| *CEBPA* | 1050 | 7.6E-04 | 0.84 | 1.3E-15 | brown | turquoise |
| *MRPL13* | 28998 | 7.6E-04 | 0.82 | 5.9E-14 | pink | green |
| *PAM* | 5066 | 7.6E-04 | 0.66 | 7.1E-08 | darkgrey | yellow |
| *STMN3* | 50861 | 7.6E-04 | -0.65 | 1.0E-07 | brown | turquoise |
| *ECHDC1* | 55862 | 7.6E-04 | 0.72 | 8.9E-10 | royalblue | turquoise |
| *TMEM135* | 65084 | 7.6E-04 | 0.86 | 1.3E-16 | brown | turquoise |
| *NFIA* | 4774 | 7.7E-04 | 0.62 | 4.6E-07 | blue | yellow |
| *POLR3E* | 55718 | 7.7E-04 | 0.55 | 1.9E-05 | black | turquoise |
| *SENP2* | 59343 | 7.7E-04 | 0.80 | 3.1E-13 | black | red |
| *COL6A2* | 1292 | 7.8E-04 | 0.60 | 1.6E-06 | cyan | royalblue |
| *MMADHC* | 27249 | 7.8E-04 | 0.80 | 2.3E-13 | pink | green |
| *DNAJB9* | 4189 | 7.8E-04 | 0.78 | 2.7E-12 | pink | green |
| *ARHGEF10L* | 55160 | 7.8E-04 | -0.54 | 2.9E-05 | brown | yellow |
| *C2* | 717 | 7.8E-04 | 0.87 | 2.8E-17 | cyan | yellow |
| *ANAPC16* | 119504 | 7.9E-04 | 0.80 | 2.9E-13 | black | turquoise |
| *SLC41A1* | 254428 | 7.9E-04 | 0.77 | 6.8E-12 | brown | turquoise |
| *UQCR10* | 29796 | 7.9E-04 | 0.87 | 5.3E-18 | brown | turquoise |
| *PBRM1* | 55193 | 7.9E-04 | 0.72 | 6.0E-10 | black | red |
| *UBA5* | 79876 | 7.9E-04 | 0.82 | 2.8E-14 | pink | green |
| *TMEM98* | 26022 | 8.0E-04 | 0.73 | 3.3E-10 | cyan | yellow |
| *CCPG1* | 9236 | 8.0E-04 | 0.68 | 1.3E-08 | darkgrey | yellow |
| *KRTCAP2* | 200185 | 8.1E-04 | 0.59 | 2.2E-06 | blue | turquoise |
| *SENP3* | 26168 | 8.1E-04 | 0.59 | 2.1E-06 | brown | turquoise |
| *ARL5A* | 26225 | 8.1E-04 | 0.53 | 3.2E-05 | pink | green |
| *GRN* | 2896 | 8.1E-04 | 0.76 | 3.9E-11 | cyan | yellow |
| *ZDHHC13* | 54503 | 8.1E-04 | -0.61 | 7.9E-07 | brown | turquoise |
| *RPP14* | 11102 | 8.2E-04 | 0.62 | 4.8E-07 | brown | turquoise |
| *UBR3* | 130507 | 8.2E-04 | 0.82 | 2.4E-14 | black | darkgrey |
| *FNTA* | 2339 | 8.2E-04 | 0.64 | 2.2E-07 | blue | darkgreen |
| *CD209* | 30835 | 8.2E-04 | 0.72 | 1.2E-09 | cyan | NA |
| *AQP7* | 364 | 8.2E-04 | 0.83 | 8.2E-15 | brown | turquoise |
| *ITGA5* | 3678 | 8.2E-04 | -0.83 | 5.4E-15 | pink | green |
| *PROS1* | 5627 | 8.2E-04 | -0.79 | 1.7E-12 | brown | turquoise |
| *SECISBP2L* | 9728 | 8.2E-04 | 0.60 | 1.8E-06 | blue | turquoise |
| *PAPOLA* | 10914 | 8.4E-04 | 0.75 | 5.4E-11 | pink | darkgreen |
| *FAM13B* | 51306 | 8.4E-04 | -0.69 | 7.8E-09 | midnightblue | turquoise |
| *PNPLA2* | 57104 | 8.4E-04 | 0.76 | 1.9E-11 | brown | turquoise |
| *USP30* | 84749 | 8.4E-04 | 0.67 | 2.8E-08 | brown | turquoise |
| *TMEM220* | 388335 | 8.5E-04 | 0.75 | 9.1E-11 | blue | turquoise |
| *SNX30* | 401548 | 8.6E-04 | -0.60 | 1.4E-06 | blue | yellow |
| *ARV1* | 64801 | 8.6E-04 | 0.84 | 2.9E-15 | pink | green |
| *ZFAND5* | 7763 | 8.6E-04 | 0.83 | 4.9E-15 | black | red |
| *ZC3H15* | 55854 | 8.7E-04 | 0.53 | 4.2E-05 | blue | turquoise |
| *XYLT1* | 64131 | 8.7E-04 | -0.67 | 3.0E-08 | brown | yellow |
| *PAPD4* | 167153 | 8.8E-04 | 0.77 | 1.3E-11 | purple | red |
| *ARRDC2* | 27106 | 8.8E-04 | -0.56 | 9.3E-06 | blue | green |
| *FRZB* | 2487 | 8.9E-04 | -0.37 | 6.1E-03 | black | turquoise |
| *RNF170* | 81790 | 8.9E-04 | 0.82 | 2.4E-14 | pink | turquoise |
| *FGFBP2* | 83888 | 8.9E-04 | -0.63 | 2.6E-07 | darkgrey | purple |
| *SLC19A2* | 10560 | 9.0E-04 | 0.81 | 1.2E-13 | black | red |
| *ATP2B1* | 490 | 9.0E-04 | -0.87 | 9.1E-18 | blue | yellow |
| *GALNT7* | 51809 | 9.0E-04 | -0.84 | 2.7E-15 | blue | turquoise |
| *ANKRD5* | 63926 | 9.0E-04 | 0.58 | 4.6E-06 | blue | NA |
| *PARK7* | 11315 | 9.1E-04 | 0.75 | 8.3E-11 | pink | turquoise |
| *HTT* | 3064 | 9.1E-04 | -0.44 | 9.5E-04 | black | green |
| *CDK14* | 5218 | 9.1E-04 | -0.51 | 7.9E-05 | black | red |
| *GSTCD* | 79807 | 9.1E-04 | 0.66 | 5.7E-08 | pink | NA |
| *WDR31* | 114987 | 9.2E-04 | 0.56 | 1.2E-05 | pink | turquoise |
| *C14orf180* | 400258 | 9.2E-04 | 0.81 | 6.4E-14 | brown | turquoise |
| *IFT20* | 90410 | 9.2E-04 | 0.60 | 1.4E-06 | cyan | yellow |
| *NBN* | 4683 | 9.3E-04 | 0.53 | 3.6E-05 | brown | turquoise |
| *ST5* | 6764 | 9.3E-04 | -0.48 | 2.6E-04 | brown | royalblue |
| *ARIH2* | 10425 | 9.4E-04 | 0.58 | 3.5E-06 | brown | turquoise |
| *C11orf58* | 10944 | 9.4E-04 | 0.61 | 8.7E-07 | pink | darkgreen |
| *EFCAB2* | 84288 | 9.4E-04 | 0.41 | 2.0E-03 | blue | turquoise |
| *SLC38A2* | 54407 | 9.5E-04 | -0.62 | 4.8E-07 | brown | darkred |
| *HLA-DPB1* | 3115 | 9.6E-04 | -0.70 | 3.6E-09 | blue | turquoise |
| *USP25* | 29761 | 9.7E-04 | 0.68 | 1.5E-08 | black | red |
| *CEBPB* | 1051 | 9.8E-04 | 0.47 | 3.6E-04 | brown | turquoise |
| *IDH2* | 3418 | 9.8E-04 | 0.69 | 9.1E-09 | brown | turquoise |
| *CAST* | 831 | 9.9E-04 | 0.52 | 5.2E-05 | purple | red |
| *BTG3* | 10950 | 1.0E-03 | 0.82 | 5.5E-14 | black | turquoise |
| *LGR4* | 55366 | 1.0E-03 | 0.61 | 8.0E-07 | black | turquoise |

DE = differentially expressed; MM=module membership
