# Supplementary material for: Coexpression Network Analysis in Abdominal and Gluteal Adipose Tissue Reveals Regulatory Genetic Loci for Metabolic Syndrome and Related Phenotypes
Source: PLoS Genet. 2012 Feb 23;8(2):e1002505. doi: 10.1371/journal.pgen.1002505 (PMC3285582; doi:10.1371/journal.pgen.1002505)
Supplement: Table S2 — Genes differentially expressed between MetS cases and controls in GLU (‘single gene analysis’). (DOC) [file pgen.1002505.s009.doc]

**Table S2** Genes differentially expressed between MetS cases and controls in GLU (‘single gene analysis’)

| **HGNC** | **GENE_ID** | **MetS DE pvalue** | **MM** | **MM pvalue** | **Module** | **Consensus module** |
| --- | --- | --- | --- | --- | --- | --- |
| *NAALAD2* | 10003 | 4.3E-07 | 0.53 | 6.5E-06 | turquoise | yellow |
| *RANBP9* | 10048 | 1.4E-04 | 0.85 | 1.7E-19 | royalblue | darkgreen |
| *FAM13A* | 10144 | 2.2E-04 | 0.76 | 2.6E-13 | royalblue | red |
| *AASS* | 10157 | 7.3E-07 | 0.67 | 7.4E-10 | brown | red |
| *LHFPL2* | 10184 | 1.0E-04 | 0.87 | 9.7E-21 | black | yellow |
| *CALCRL* | 10203 | 1.3E-05 | 0.62 | 4.3E-08 | royalblue | yellow |
| *PRG4* | 10216 | 2.5E-05 | 0.65 | 4.7E-09 | darkred | yellow |
| *PLIN3* | 10226 | 1.4E-07 | 0.77 | 1.0E-13 | darkgreen | yellow |
| *MRPS31* | 10240 | 1.3E-04 | 0.64 | 8.2E-09 | brown | turquoise |
| *GPHN* | 10243 | 1.5E-04 | 0.73 | 6.8E-12 | brown | yellow |
| *HRSP12* | 10247 | 4.2E-06 | 0.82 | 3.1E-17 | turquoise | turquoise |
| *CDKN1C* | 1028 | 9.4E-05 | 0.53 | 5.8E-06 | brown | turquoise |
| *TNIP1* | 10318 | 5.6E-05 | 0.55 | 2.2E-06 | red | turquoise |
| *CDKN2C* | 1031 | 1.8E-06 | 0.80 | 2.1E-15 | turquoise | turquoise |
| *ABCA8* | 10351 | 3.6E-04 | -0.24 | 5.3E-02 | turquoise | turquoise |
| *CITED2* | 10370 | 2.0E-05 | 0.79 | 3.2E-15 | royalblue | red |
| *ST3GAL6* | 10402 | 4.9E-06 | 0.65 | 3.5E-09 | brown | yellow |
| *SPON1* | 10418 | 1.2E-04 | 0.71 | 3.5E-11 | royalblue | red |
| *SEC24B* | 10427 | 1.0E-06 | -0.60 | 1.0E-07 | darkred | red |
| *OLFM1* | 10439 | 1.6E-04 | -0.60 | 1.4E-07 | turquoise | yellow |
| *CLEC10A* | 10462 | 2.7E-04 | 0.71 | 3.1E-11 | darkred | yellow |
| *HEXIM1* | 10614 | 1.7E-04 | 0.66 | 2.4E-09 | darkgreen | purple |
| *TGOLN2* | 10618 | 2.2E-06 | 0.57 | 7.5E-07 | black | yellow |
| *ALDH1L1* | 10840 | 5.1E-05 | 0.85 | 4.2E-19 | turquoise | turquoise |
| *CKAP4* | 10970 | 4.5E-06 | 0.56 | 1.5E-06 | darkred | yellow |
| *LILRB5* | 10990 | 3.3E-04 | 0.84 | 4.0E-18 | darkred | yellow |
| *FSTL1* | 11167 | 6.5E-06 | 0.73 | 5.3E-12 | darkred | yellow |
| *INMT* | 11185 | 1.5E-05 | -0.74 | 2.1E-12 | royalblue | yellow |
| *NISCH* | 11188 | 3.2E-04 | -0.70 | 7.5E-11 | tan | green |
| *CHP* | 11261 | 2.6E-07 | 0.72 | 1.1E-11 | turquoise | turquoise |
| *C1orf85* | 112770 | 3.7E-05 | 0.73 | 5.3E-12 | darkred | yellow |
| *GLCCI1* | 113263 | 6.2E-05 | 0.65 | 4.2E-09 | brown | yellow |
| *PHB2* | 11331 | 2.1E-04 | 0.27 | 2.7E-02 | turquoise | turquoise |
| *C1QTNF5* | 114902 | 7.6E-06 | 0.74 | 2.6E-12 | darkgreen | yellow |
| *VASN* | 114990 | 2.6E-04 | 0.60 | 1.1E-07 | darkgreen | yellow |
| *CKB* | 1152 | 3.0E-04 | 0.75 | 3.9E-13 | turquoise | turquoise |
| *GPR146* | 115330 | 2.6E-04 | 0.69 | 1.4E-10 | royalblue | red |
| *C7orf30* | 115416 | 2.8E-04 | 0.81 | 2.1E-16 | turquoise | turquoise |
| *TOP1MT* | 116447 | 1.0E-04 | -0.58 | 4.4E-07 | brown | turquoise |
| *CLU* | 1191 | 2.9E-05 | 0.78 | 2.7E-14 | darkgreen | yellow |
| *CPXM2* | 119587 | 3.3E-04 | -0.37 | 2.5E-03 | turquoise | yellow |
| *TPP1* | 1200 | 1.5E-04 | -0.73 | 6.1E-12 | brown | yellow |
| *FAT3* | 120114 | 3.1E-05 | 0.68 | 4.4E-10 | turquoise | turquoise |
| *NTAN1* | 123803 | 1.6E-04 | 0.66 | 2.4E-09 | black | yellow |
| *CMTM3* | 123920 | 3.9E-04 | 0.73 | 5.8E-12 | darkred | yellow |
| *NCRNA00188* | 125144 | 9.6E-07 | 0.60 | 1.2E-07 | brown | turquoise |
| *ADH1B* | 125 | 9.5E-05 | 0.76 | 1.2E-13 | pink | red |
| *C1orf216* | 127703 | 2.7E-05 | 0.63 | 1.9E-08 | darkgreen | yellow |
| *C22orf25* | 128989 | 1.5E-05 | -0.58 | 5.3E-07 | brown | turquoise |
| *COL5A2* | 1290 | 1.9E-05 | 0.74 | 2.7E-12 | darkgreen | yellow |
| *ACVR1C* | 130399 | 1.6E-04 | 0.68 | 3.2E-10 | turquoise | yellow |
| *ZFAND2B* | 130617 | 2.8E-05 | -0.38 | 1.8E-03 | turquoise | turquoise |
| *COL16A1* | 1307 | 7.4E-06 | 0.81 | 1.6E-16 | darkred | yellow |
| *COMP* | 1311 | 2.4E-04 | -0.62 | 4.7E-08 | brown | NA |
| *NUDT16* | 131870 | 3.8E-04 | 0.44 | 2.9E-04 | brown | turquoise |
| *CD109* | 135228 | 3.1E-04 | 0.72 | 1.2E-11 | black | yellow |
| *CSF1R* | 1436 | 2.9E-05 | 0.81 | 1.8E-16 | darkred | yellow |
| *SLC38A6* | 145389 | 1.4E-04 | 0.70 | 6.0E-11 | black | yellow |
| *CSRP2* | 1466 | 2.2E-06 | 0.81 | 2.3E-16 | pink | purple |
| *CCDC80* | 151887 | 2.4E-07 | 0.75 | 4.4E-13 | darkred | yellow |
| *CCDC50* | 152137 | 1.5E-04 | 0.80 | 6.7E-16 | royalblue | red |
| *CYB5A* | 1528 | 5.8E-05 | 0.72 | 2.2E-11 | brown | turquoise |
| *CYB561* | 1534 | 7.8E-06 | -0.63 | 1.7E-08 | royalblue | yellow |
| *DAB2* | 1601 | 2.6E-06 | 0.83 | 2.0E-17 | darkred | yellow |
| *AP2A2* | 161 | 8.8E-05 | 0.61 | 9.0E-08 | darkred | royalblue |
| *AP1B1* | 162 | 6.6E-05 | 0.56 | 1.3E-06 | red | turquoise |
| *DCI* | 1632 | 3.1E-05 | 0.74 | 1.2E-12 | turquoise | turquoise |
| *DECR1* | 1666 | 1.9E-05 | 0.86 | 1.8E-20 | turquoise | turquoise |
| *DFNA5* | 1687 | 2.0E-04 | 0.68 | 5.8E-10 | black | yellow |
| *NQO1* | 1728 | 3.5E-07 | 0.75 | 6.3E-13 | darkgreen | yellow |
| *DNM1* | 1759 | 8.6E-05 | 0.82 | 3.4E-17 | darkred | royalblue |
| *LDHD* | 197257 | 1.4E-04 | 0.87 | 1.5E-20 | turquoise | turquoise |
| *ESD* | 2098 | 7.1E-05 | 0.73 | 6.7E-12 | royalblue | red |
| *ETFA* | 2108 | 1.0E-04 | 0.73 | 4.2E-12 | turquoise | turquoise |
| *EXT1* | 2131 | 1.8E-05 | 0.61 | 5.9E-08 | darkred | yellow |
| *PLAC9* | 219348 | 8.3E-06 | 0.70 | 6.5E-11 | darkgreen | yellow |
| *STOX1* | 219736 | 4.9E-07 | -0.71 | 4.5E-11 | darkred | yellow |
| *FBN1* | 2200 | 2.7E-04 | 0.76 | 2.1E-13 | darkred | yellow |
| *EFEMP1* | 2202 | 3.9E-04 | 0.72 | 1.1E-11 | darkred | yellow |
| *ALDH1A3* | 220 | 2.7E-07 | 0.85 | 1.6E-19 | darkred | yellow |
| *LNX2* | 222484 | 1.4E-04 | -0.57 | 6.3E-07 | darkgreen | royalblue |
| *DLGAP4* | 22839 | 2.5E-04 | 0.67 | 1.1E-09 | darkgreen | royalblue |
| *PPRC1* | 23082 | 1.9E-06 | -0.51 | 1.6E-05 | turquoise | royalblue |
| *TBC1D2B* | 23102 | 9.1E-05 | -0.36 | 3.1E-03 | turquoise | green |
| *STAB1* | 23166 | 5.1E-06 | 0.80 | 1.1E-15 | darkred | yellow |
| *GPD1L* | 23171 | 1.1E-07 | 0.71 | 4.8E-11 | royalblue | yellow |
| *LPIN1* | 23175 | 2.5E-04 | 0.63 | 1.8E-08 | brown | yellow |
| *SEPT8* | 23176 | 6.3E-06 | 0.73 | 3.8E-12 | darkgreen | yellow |
| *RGL1* | 23179 | 6.1E-05 | 0.57 | 8.5E-07 | darkred | yellow |
| *MESDC2* | 23184 | 1.4E-06 | 0.72 | 2.0E-11 | darkgreen | yellow |
| *COBL* | 23242 | 7.2E-05 | 0.61 | 5.3E-08 | turquoise | NA |
| *FKBP15* | 23307 | 3.4E-04 | 0.75 | 6.2E-13 | black | yellow |
| *C22orf9* | 23313 | 2.4E-05 | 0.89 | 1.8E-23 | black | yellow |
| *WDR7* | 23335 | 3.6E-05 | 0.49 | 2.9E-05 | magenta | yellow |
| *PIP5K1C* | 23396 | 7.8E-05 | 0.70 | 8.4E-11 | red | black |
| *SIRT1* | 23411 | 5.5E-05 | 0.66 | 2.0E-09 | pink | red |
| *CBX6* | 23466 | 2.3E-04 | -0.49 | 3.5E-05 | royalblue | royalblue |
| *FPR3* | 2359 | 3.0E-04 | 0.73 | 5.7E-12 | darkred | yellow |
| *PLA2G15* | 23659 | 1.6E-04 | 0.71 | 2.6E-11 | black | yellow |
| *CADM1* | 23705 | 2.2E-05 | 0.69 | 2.4E-10 | turquoise | turquoise |
| *FRG1* | 2483 | 7.5E-05 | 0.61 | 7.5E-08 | royalblue | darkgreen |
| *FTH1* | 2495 | 1.6E-04 | 0.73 | 8.2E-12 | black | yellow |
| *FUCA1* | 2517 | 4.6E-06 | 0.88 | 1.5E-21 | darkred | yellow |
| *TNFAIP8* | 25816 | 7.3E-05 | 0.37 | 2.4E-03 | turquoise | turquoise |
| *BACE2* | 25825 | 3.5E-05 | -0.52 | 1.1E-05 | turquoise | yellow |
| *LETMD1* | 25875 | 1.5E-04 | 0.67 | 1.1E-09 | brown | red |
| *GALNS* | 2588 | 7.5E-05 | 0.41 | 6.1E-04 | green | blue |
| *OLFML2B* | 25903 | 1.2E-04 | 0.69 | 2.1E-10 | darkred | yellow |
| *GALNT2* | 2590 | 3.5E-04 | 0.42 | 5.7E-04 | darkred | yellow |
| *GORASP2* | 26003 | 3.6E-04 | 0.63 | 2.4E-08 | red | yellow |
| *LRIG1* | 26018 | 7.6E-05 | 0.69 | 1.7E-10 | brown | yellow |
| *GATA3* | 2625 | 4.8E-05 | 0.62 | 3.2E-08 | turquoise | yellow |
| *GBAS* | 2631 | 3.4E-04 | 0.73 | 4.3E-12 | turquoise | turquoise |
| *GCDH* | 2639 | 1.5E-04 | 0.85 | 3.1E-19 | turquoise | turquoise |
| *GCHFR* | 2644 | 1.5E-05 | 0.61 | 5.1E-08 | turquoise | turquoise |
| *MYOF* | 26509 | 2.3E-04 | 0.68 | 6.4E-10 | darkgreen | yellow |
| *GDF10* | 2662 | 3.7E-04 | 0.73 | 3.3E-12 | darkred | yellow |
| *DKK3* | 27122 | 1.4E-04 | -0.70 | 1.0E-10 | royalblue | yellow |
| *SLC39A1* | 27173 | 9.6E-05 | 0.62 | 4.5E-08 | darkgreen | royalblue |
| *GLB1* | 2720 | 3.8E-05 | 0.81 | 2.6E-16 | darkgreen | purple |
| *ZNF544* | 27300 | 2.8E-04 | 0.39 | 1.4E-03 | magenta | turquoise |
| *GLUL* | 2752 | 3.8E-07 | -0.89 | 1.1E-22 | darkgreen | yellow |
| *GMFB* | 2764 | 4.7E-05 | -0.51 | 1.7E-05 | brown | yellow |
| *GNA15* | 2769 | 3.9E-04 | -0.83 | 8.2E-18 | brown | yellow |
| *GNAI1* | 2770 | 2.4E-05 | 0.69 | 2.7E-10 | brown | yellow |
| *ABL2* | 27 | 2.3E-05 | 0.59 | 2.8E-07 | black | yellow |
| *GOLGA3* | 2802 | 2.5E-06 | 0.58 | 4.7E-07 | red | yellow |
| *GPC1* | 2817 | 1.9E-06 | 0.66 | 1.6E-09 | black | yellow |
| *CD163L1* | 283316 | 2.5E-05 | 0.74 | 1.7E-12 | darkred | yellow |
| *SLC46A3* | 283537 | 4.0E-04 | 0.52 | 1.1E-05 | darkgreen | red |
| *SUMF1* | 285362 | 6.1E-06 | 0.58 | 4.4E-07 | darkred | yellow |
| *SH3PXD2B* | 285590 | 3.7E-05 | 0.54 | 4.2E-06 | darkred | royalblue |
| *C5orf51* | 285636 | 6.3E-05 | 0.70 | 1.0E-10 | pink | red |
| *GRB14* | 2888 | 2.9E-05 | 0.57 | 8.3E-07 | darkgreen | red |
| *SNX24* | 28966 | 3.4E-05 | 0.74 | 1.3E-12 | darkred | yellow |
| *KLF15* | 28999 | 2.5E-05 | 0.58 | 3.3E-07 | turquoise | turquoise |
| *SLC43A3* | 29015 | 2.8E-05 | 0.77 | 1.1E-13 | black | yellow |
| *THYN1* | 29087 | 9.8E-05 | 0.55 | 1.7E-06 | turquoise | turquoise |
| *USP25* | 29761 | 1.4E-05 | 0.68 | 3.2E-10 | royalblue | red |
| *TAX1BP3* | 30851 | 3.7E-05 | 0.62 | 3.3E-08 | darkred | yellow |
| *ANXA11* | 311 | 2.6E-04 | 0.42 | 4.3E-04 | darkgreen | royalblue |
| *HSD11B1* | 3290 | 4.0E-04 | 0.67 | 7.5E-10 | darkred | yellow |
| *TNC* | 3371 | 1.4E-09 | 0.72 | 1.6E-11 | darkred | yellow |
| *C7orf46* | 340277 | 6.2E-05 | 0.63 | 2.6E-08 | brown | yellow |
| *NANOS1* | 340719 | 8.6E-05 | 0.67 | 1.3E-09 | darkgreen | purple |
| *IFNAR1* | 3454 | 2.1E-04 | 0.42 | 4.4E-04 | darkgreen | turquoise |
| *TUBB2B* | 347733 | 5.8E-05 | 0.74 | 1.8E-12 | darkgreen | purple |
| *APOE* | 348 | 4.3E-07 | -0.43 | 3.3E-04 | darkred | turquoise |
| *ACADM* | 34 | 4.6E-05 | 0.78 | 1.0E-14 | turquoise | turquoise |
| *IL1R1* | 3554 | 1.4E-04 | -0.63 | 1.8E-08 | turquoise | yellow |
| *IRS1* | 3667 | 2.6E-05 | 0.65 | 3.8E-09 | brown | turquoise |
| *ITGA5* | 3678 | 2.4E-04 | -0.48 | 5.8E-05 | brown | green |
| *ITGAV* | 3685 | 5.8E-07 | -0.69 | 2.0E-10 | pink | red |
| *ITGB5* | 3693 | 1.0E-10 | 0.84 | 1.1E-18 | darkgreen | yellow |
| *ACADSB* | 36 | 2.8E-04 | 0.77 | 4.6E-14 | turquoise | turquoise |
| *LRRC33* | 375387 | 2.9E-04 | -0.72 | 1.9E-11 | brown | turquoise |
| *IER5L* | 389792 | 8.4E-05 | 0.73 | 4.8E-12 | darkred | yellow |
| *ACAT1* | 38 | 2.1E-04 | 0.70 | 8.9E-11 | turquoise | turquoise |
| *LAMP1* | 3916 | 9.2E-07 | 0.65 | 4.0E-09 | black | yellow |
| *LBP* | 3929 | 6.0E-06 | 0.70 | 1.2E-10 | darkred | yellow |
| *LEP* | 3952 | 2.1E-04 | 0.77 | 4.8E-14 | cyan | purple |
| *LGALS3* | 3958 | 3.4E-04 | 0.42 | 5.5E-04 | greenyellow | turquoise |
| *C4orf48* | 401115 | 5.5E-08 | 0.56 | 9.5E-07 | black | yellow |
| *LTBP2* | 4053 | 5.3E-05 | -0.65 | 4.5E-09 | turquoise | yellow |
| *MAP1B* | 4131 | 1.2E-07 | -0.73 | 4.2E-12 | turquoise | turquoise |
| *MARS* | 4141 | 1.8E-04 | -0.43 | 3.4E-04 | brown | turquoise |
| *MGAT1* | 4245 | 6.5E-05 | 0.60 | 1.7E-07 | black | turquoise |
| *MMP19* | 4327 | 4.5E-05 | 0.65 | 4.0E-09 | black | yellow |
| *MN1* | 4330 | 1.6E-04 | -0.51 | 1.3E-05 | darkgrey | purple |
| *ABCC1* | 4363 | 2.7E-07 | 0.83 | 1.3E-17 | darkgreen | yellow |
| *MSX1* | 4487 | 1.8E-05 | 0.53 | 6.2E-06 | darkred | royalblue |
| *NAGA* | 4668 | 1.1E-04 | 0.84 | 2.1E-18 | black | yellow |
| *NDUFB5* | 4711 | 1.8E-04 | 0.88 | 1.4E-22 | turquoise | turquoise |
| *NDUFB8* | 4714 | 2.0E-04 | 0.72 | 1.2E-11 | turquoise | turquoise |
| *NDUFS4* | 4724 | 3.0E-04 | 0.62 | 3.9E-08 | turquoise | turquoise |
| *NNMT* | 4837 | 7.8E-05 | 0.67 | 1.2E-09 | darkred | yellow |
| *NTRK3* | 4916 | 5.5E-05 | 0.61 | 6.0E-08 | brown | turquoise |
| *PHOSPHO2* | 493911 | 1.5E-04 | 0.30 | 1.6E-02 | turquoise | turquoise |
| *OGN* | 4969 | 1.9E-04 | 0.55 | 1.6E-06 | darkred | yellow |
| *ALDH7A1* | 501 | 1.2E-05 | 0.71 | 4.5E-11 | brown | turquoise |
| *PAM* | 5066 | 1.3E-06 | 0.71 | 2.5E-11 | darkred | yellow |
| *CNTN3* | 5067 | 1.6E-04 | -0.53 | 5.5E-06 | brown | yellow |
| *PCBD1* | 5092 | 2.5E-06 | 0.76 | 1.4E-13 | turquoise | turquoise |
| *ATP5C1* | 509 | 2.1E-04 | 0.85 | 4.6E-19 | turquoise | turquoise |
| *NMD3* | 51068 | 6.5E-05 | 0.67 | 1.2E-09 | royalblue | darkgreen |
| *LACTB2* | 51110 | 1.2E-05 | 0.84 | 1.8E-18 | turquoise | turquoise |
| *GOLM1* | 51280 | 1.6E-04 | 0.68 | 6.5E-10 | darkred | yellow |
| *NRN1* | 51299 | 2.8E-04 | 0.56 | 1.1E-06 | brown | yellow |
| *GULP1* | 51454 | 1.0E-06 | 0.56 | 1.2E-06 | brown | yellow |
| *CWC15* | 51503 | 1.9E-04 | 0.71 | 5.0E-11 | lightgreen | turquoise |
| *C14orf166* | 51637 | 4.3E-05 | 0.67 | 1.2E-09 | royalblue | turquoise |
| *BRP44L* | 51660 | 1.9E-04 | 0.71 | 3.5E-11 | turquoise | turquoise |
| *PENK* | 5179 | 2.5E-06 | 0.64 | 8.7E-09 | black | yellow |
| *PFDN2* | 5202 | 3.4E-04 | 0.77 | 6.4E-14 | turquoise | turquoise |
| *PFKFB3* | 5209 | 7.4E-05 | 0.67 | 1.3E-09 | brown | red |
| *SERPINE2* | 5270 | 5.7E-05 | -0.63 | 2.4E-08 | royalblue | red |
| *PKP2* | 5318 | 1.2E-04 | 0.77 | 1.1E-13 | pink | red |
| *SPA17* | 53340 | 1.5E-04 | 0.57 | 9.3E-07 | darkred | yellow |
| *PMM1* | 5372 | 7.1E-05 | 0.83 | 1.2E-17 | royalblue | red |
| *CHPF2* | 54480 | 1.9E-05 | -0.62 | 4.6E-08 | brown | turquoise |
| *FAM105A* | 54491 | 1.2E-04 | 0.73 | 3.5E-12 | darkred | NA |
| *NDFIP2* | 54602 | 1.1E-06 | 0.61 | 5.9E-08 | turquoise | turquoise |
| *EPDR1* | 54749 | 1.5E-04 | 0.72 | 1.7E-11 | darkgreen | yellow |
| *C20orf27* | 54976 | 8.6E-05 | 0.70 | 7.6E-11 | greenyellow | turquoise |
| *C2orf18* | 54978 | 2.1E-04 | 0.56 | 1.0E-06 | darkred | darkturquoise |
| *HCFC1R1* | 54985 | 3.2E-04 | 0.51 | 1.4E-05 | black | royalblue |
| *CCDC109B* | 55013 | 3.7E-04 | 0.73 | 7.0E-12 | darkred | yellow |
| *ARHGEF10L* | 55160 | 1.3E-05 | 0.67 | 9.3E-10 | darkred | yellow |
| *SLC38A7* | 55238 | 1.1E-05 | 0.62 | 3.8E-08 | red | black |
| *RHOT1* | 55288 | 4.8E-05 | 0.75 | 4.9E-13 | brown | yellow |
| *SLC29A3* | 55315 | 3.4E-04 | 0.88 | 6.5E-22 | black | yellow |
| *NIPSNAP3B* | 55335 | 1.4E-04 | 0.85 | 3.3E-19 | royalblue | red |
| *TMEM176A* | 55365 | 1.6E-04 | 0.81 | 3.4E-16 | darkred | royalblue |
| *PPT1* | 5538 | 2.4E-04 | 0.83 | 1.4E-17 | black | yellow |
| *H2AFY2* | 55506 | 4.3E-05 | 0.73 | 6.2E-12 | darkgreen | yellow |
| *SLC39A4* | 55630 | 3.1E-05 | 0.50 | 2.2E-05 | darkgreen | royalblue |
| *TMEM127* | 55654 | 8.2E-06 | -0.71 | 3.7E-11 | brown | yellow |
| *ADAP2* | 55803 | 2.7E-06 | 0.76 | 3.2E-13 | darkred | yellow |
| *PECR* | 55825 | 1.8E-04 | 0.71 | 4.0E-11 | darkturquoise | turquoise |
| *GLT8D1* | 55830 | 6.1E-05 | -0.58 | 4.6E-07 | turquoise | darkred |
| *GSDMB* | 55876 | 5.3E-05 | 0.71 | 4.7E-11 | royalblue | red |
| *BCAP29* | 55973 | 3.0E-04 | 0.76 | 1.4E-13 | turquoise | turquoise |
| *SLC7A10* | 56301 | 5.6E-05 | 0.79 | 3.5E-15 | turquoise | turquoise |
| *AZGP1* | 563 | 4.7E-05 | 0.78 | 3.2E-14 | turquoise | turquoise |
| *LGMN* | 5641 | 2.1E-05 | 0.86 | 2.2E-20 | darkred | yellow |
| *HTRA1* | 5654 | 3.6E-05 | 0.81 | 4.0E-16 | darkgreen | yellow |
| *SLC2A4RG* | 56731 | 2.1E-04 | 0.68 | 3.6E-10 | brown | turquoise |
| *SLAMF8* | 56833 | 1.3E-04 | 0.89 | 3.5E-23 | black | yellow |
| *NDUFA4L2* | 56901 | 2.3E-04 | 0.64 | 1.2E-08 | brown | turquoise |
| *SEMA3G* | 56920 | 5.5E-05 | -0.62 | 3.4E-08 | turquoise | turquoise |
| *MRPS22* | 56945 | 2.0E-05 | 0.73 | 5.4E-12 | turquoise | turquoise |
| *PRTFDC1* | 56952 | 1.9E-05 | -0.59 | 2.8E-07 | turquoise | yellow |
| *CABC1* | 56997 | 5.8E-07 | 0.70 | 9.4E-11 | brown | yellow |
| *CD248* | 57124 | 3.0E-06 | 0.84 | 3.6E-18 | darkgreen | yellow |
| *PLXDC1* | 57125 | 1.3E-05 | 0.81 | 2.5E-16 | darkred | yellow |
| *SLC39A10* | 57181 | 3.0E-04 | 0.53 | 6.4E-06 | darkgreen | turquoise |
| *PTEN* | 5728 | 4.0E-04 | -0.68 | 3.2E-10 | darkgreen | yellow |
| *RCN3* | 57333 | 2.0E-06 | 0.61 | 8.7E-08 | darkred | royalblue |
| *PTGFRN* | 5738 | 1.0E-05 | 0.80 | 7.1E-16 | darkred | yellow |
| *GALNTL1* | 57452 | 2.9E-05 | 0.74 | 1.2E-12 | darkred | yellow |
| *ALPK3* | 57538 | 6.3E-05 | 0.74 | 1.2E-12 | brown | yellow |
| *KLHDC5* | 57542 | 4.7E-05 | -0.48 | 4.8E-05 | brown | turquoise |
| *TSHZ3* | 57616 | 2.2E-05 | -0.60 | 1.1E-07 | turquoise | turquoise |
| *KIAA1598* | 57698 | 7.7E-05 | 0.80 | 2.5E-15 | black | yellow |
| *BCKDHB* | 594 | 3.3E-04 | 0.85 | 2.4E-19 | turquoise | turquoise |
| *RPL30* | 6156 | 1.8E-04 | 0.70 | 6.1E-11 | royalblue | red |
| *RPN1* | 6184 | 3.6E-04 | 0.63 | 2.4E-08 | darkred | red |
| *RPN2* | 6185 | 6.6E-08 | 0.82 | 1.0E-16 | darkgreen | purple |
| *BDKRB2* | 624 | 4.4E-05 | 0.65 | 6.4E-09 | darkred | yellow |
| *S100A11* | 6282 | 1.1E-05 | 0.78 | 2.2E-14 | darkgreen | royalblue |
| *CFB* | 629 | 9.2E-05 | 0.78 | 2.3E-14 | darkred | yellow |
| *CCL19* | 6363 | 3.3E-04 | -0.55 | 2.4E-06 | brown | turquoise |
| *PKNOX2* | 63876 | 1.9E-04 | 0.55 | 1.8E-06 | turquoise | turquoise |
| *THADA* | 63892 | 4.6E-06 | 0.59 | 1.8E-07 | black | yellow |
| *XYLT1* | 64131 | 8.2E-05 | 0.77 | 3.6E-14 | darkgreen | yellow |
| *SFRP4* | 6424 | 8.4E-08 | 0.74 | 1.7E-12 | darkgreen | yellow |
| *CREB3L2* | 64764 | 1.8E-08 | -0.51 | 1.6E-05 | turquoise | yellow |
| *GINS3* | 64785 | 1.1E-05 | 0.79 | 2.8E-15 | pink | red |
| *FNDC4* | 64838 | 2.8E-04 | 0.57 | 6.9E-07 | darkgreen | royalblue |
| *MRPL32* | 64983 | 3.4E-05 | 0.69 | 2.0E-10 | royalblue | turquoise |
| *NDRG4* | 65009 | 3.5E-07 | 0.79 | 3.4E-15 | pink | red |
| *SMARCA4* | 6597 | 4.1E-05 | 0.63 | 2.4E-08 | darkgreen | purple |
| *SIGLEC1* | 6614 | 4.8E-05 | 0.74 | 1.5E-12 | darkred | yellow |
| *BNIP3L* | 665 | 1.2E-04 | -0.45 | 1.7E-04 | darkred | turquoise |
| *ST5* | 6764 | 1.2E-04 | 0.52 | 8.7E-06 | darkred | royalblue |
| *SULT1A2* | 6799 | 1.6E-05 | -0.42 | 5.0E-04 | brown | yellow |
| *SVIL* | 6840 | 3.4E-04 | -0.51 | 1.3E-05 | black | yellow |
| *THY1* | 7070 | 1.0E-04 | 0.48 | 6.0E-05 | darkred | yellow |
| *GPR137B* | 7107 | 1.4E-05 | 0.80 | 1.7E-15 | darkred | yellow |
| *TNFRSF1A* | 7132 | 4.4E-05 | -0.63 | 2.2E-08 | brown | yellow |
| *C1QB* | 713 | 6.4E-05 | 0.80 | 7.6E-16 | darkred | yellow |
| *C1QC* | 714 | 1.5E-04 | 0.87 | 4.3E-21 | darkred | yellow |
| *TPD52L1* | 7164 | 1.3E-06 | 0.71 | 3.6E-11 | turquoise | yellow |
| *C1S* | 716 | 2.3E-04 | 0.80 | 1.1E-15 | darkred | yellow |
| *C2* | 717 | 1.5E-05 | 0.86 | 2.0E-20 | darkred | yellow |
| *C3AR1* | 719 | 4.7E-05 | 0.77 | 4.4E-14 | darkred | yellow |
| *PHLDA2* | 7262 | 3.9E-08 | 0.84 | 9.5E-19 | darkgreen | yellow |
| *TWIST1* | 7291 | 2.0E-08 | 0.79 | 7.8E-15 | royalblue | red |
| *TXNRD1* | 7296 | 6.3E-05 | 0.81 | 2.8E-16 | darkgreen | yellow |
| *UCHL1* | 7345 | 1.3E-05 | 0.60 | 1.4E-07 | darkred | yellow |
| *VEGFA* | 7422 | 1.4E-04 | -0.72 | 8.7E-12 | black | yellow |
| *SLC30A1* | 7779 | 1.2E-04 | 0.73 | 3.6E-12 | black | yellow |
| *PTP4A1* | 7803 | 1.6E-04 | 0.65 | 5.3E-09 | brown | darkgreen |
| *BTG2* | 7832 | 1.2E-04 | -0.62 | 4.4E-08 | darkgreen | yellow |
| *REEP5* | 7905 | 3.7E-04 | 0.62 | 3.4E-08 | darkgreen | yellow |
| *C11orf49* | 79096 | 8.6E-05 | 0.66 | 2.1E-09 | darkred | yellow |
| *ACSS3* | 79611 | 3.6E-07 | 0.72 | 1.6E-11 | brown | yellow |
| *PLEKHF2* | 79666 | 7.6E-05 | 0.48 | 5.2E-05 | royalblue | royalblue |
| *AAGAB* | 79719 | 1.1E-04 | 0.51 | 1.5E-05 | darkgreen | yellow |
| *MAFK* | 7975 | 3.1E-04 | -0.50 | 1.8E-05 | turquoise | darkorange |
| *TXNDC15* | 79770 | 7.7E-06 | 0.70 | 1.1E-10 | darkgreen | yellow |
| *ATP8B4* | 79895 | 3.2E-04 | 0.70 | 9.3E-11 | royalblue | red |
| *TMEM22* | 80723 | 1.3E-05 | 0.63 | 1.9E-08 | turquoise | turquoise |
| *ITIH5* | 80760 | 2.2E-06 | 0.69 | 2.4E-10 | darkgreen | purple |
| *C12orf39* | 80763 | 5.2E-06 | -0.62 | 4.7E-08 | darkgreen | yellow |
| *MFAP5* | 8076 | 2.8E-04 | 0.66 | 1.7E-09 | darkgreen | yellow |
| *FXR1* | 8087 | 2.9E-04 | -0.51 | 1.3E-05 | red | red |
| *SYNC* | 81493 | 6.2E-07 | 0.81 | 3.4E-16 | darkgreen | purple |
| *NRIP1* | 8204 | 3.9E-07 | 0.81 | 2.8E-16 | royalblue | red |
| *CAST* | 831 | 3.2E-04 | 0.61 | 5.4E-08 | pink | red |
| *GRWD1* | 83743 | 3.7E-04 | 0.59 | 1.8E-07 | red | black |
| *KCTD10* | 83892 | 9.2E-08 | -0.66 | 3.2E-09 | royalblue | yellow |
| *RASSF4* | 83937 | 1.3E-04 | 0.42 | 4.3E-04 | darkred | turquoise |
| *LOXL4* | 84171 | 2.9E-05 | 0.59 | 2.5E-07 | black | yellow |
| *TMEM117* | 84216 | 6.3E-05 | 0.54 | 4.1E-06 | magenta | turquoise |
| *RPAIN* | 84268 | 1.6E-05 | 0.63 | 1.7E-08 | turquoise | turquoise |
| *CARD6* | 84674 | 2.0E-09 | -0.61 | 6.3E-08 | brown | yellow |
| *PPFIBP2* | 8495 | 3.6E-06 | 0.80 | 9.6E-16 | darkgreen | yellow |
| *MFSD5* | 84975 | 3.6E-04 | 0.64 | 9.7E-09 | darkred | purple |
| *COPS3* | 8533 | 9.5E-05 | 0.53 | 5.7E-06 | turquoise | turquoise |
| *YARS* | 8565 | 1.7E-04 | 0.52 | 7.4E-06 | darkred | yellow |
| *PRKRA* | 8575 | 1.5E-04 | 0.80 | 1.3E-15 | lightgreen | turquoise |
| *IRS2* | 8660 | 5.1E-05 | 0.61 | 5.1E-08 | brown | yellow |
| *MPDZ* | 8777 | 1.9E-04 | 0.69 | 1.8E-10 | brown | yellow |
| *WISP2* | 8839 | 5.3E-06 | -0.57 | 5.9E-07 | turquoise | yellow |
| *P4HA2* | 8974 | 3.4E-05 | 0.76 | 2.7E-13 | darkred | yellow |
| *TRIM47* | 91107 | 2.5E-05 | 0.63 | 1.7E-08 | darkred | royalblue |
| *PDLIM1* | 9124 | 1.2E-04 | 0.71 | 4.3E-11 | darkred | yellow |
| *ARRDC4* | 91947 | 3.2E-06 | -0.61 | 7.4E-08 | brown | yellow |
| *MRPS36* | 92259 | 4.6E-05 | 0.61 | 6.6E-08 | turquoise | turquoise |
| *CCPG1* | 9236 | 1.8E-05 | 0.77 | 9.7E-14 | darkgreen | yellow |
| *FAM114A1* | 92689 | 3.3E-04 | 0.41 | 7.2E-04 | grey60 | turquoise |
| *SLIT2* | 9353 | 1.4E-05 | -0.59 | 2.4E-07 | royalblue | red |
| *MRFAP1* | 93621 | 2.5E-06 | 0.59 | 3.1E-07 | darkred | turquoise |
| *ORMDL3* | 94103 | 1.5E-04 | -0.74 | 1.5E-12 | orange | turquoise |
| *TJP2* | 9414 | 6.2E-05 | 0.61 | 5.5E-08 | royalblue | yellow |
| *BAG3* | 9531 | 2.0E-06 | 0.81 | 5.2E-16 | darkgreen | purple |
| *PRDX6* | 9588 | 7.9E-05 | 0.77 | 1.1E-13 | royalblue | red |
| *PDIA4* | 9601 | 8.3E-05 | 0.66 | 1.7E-09 | black | yellow |
| *FEZ1* | 9638 | 1.6E-05 | 0.62 | 3.4E-08 | darkgreen | yellow |
| *TRAM2* | 9697 | 8.6E-06 | 0.74 | 1.4E-12 | darkred | yellow |
| *TOMM20* | 9804 | 2.0E-04 | 0.71 | 3.3E-11 | lightgreen | turquoise |
| *SCRN1* | 9805 | 1.7E-04 | -0.77 | 5.7E-14 | darkorange | yellow |
| *PSMD6* | 9861 | 2.1E-04 | 0.63 | 1.8E-08 | lightgreen | green |
| *KIAA0415* | 9907 | 3.7E-04 | -0.45 | 1.9E-04 | brown | turquoise |

DE = differentially expressed; MM=module membership
