# Supplementary material for: Coexpression Network Analysis in Abdominal and Gluteal Adipose Tissue Reveals Regulatory Genetic Loci for Metabolic Syndrome and Related Phenotypes
Source: PLoS Genet. 2012 Feb 23;8(2):e1002505. doi: 10.1371/journal.pgen.1002505 (PMC3285582; doi:10.1371/journal.pgen.1002505)
Supplement: Table S3 — Module membership and association with MetS and BMI in the MolOBB ABD dataset of genes identified by Emilsson et al. [11] as part of a macrophage-enriched metabolic network in subcutaneous adipose tissue and associated with obesity-related traits. (DOC) [file pgen.1002505.s010.doc]

**Table S3** Module membership and association with MetS and BMI in the MolOBB ABD dataset of genes identified by Emilsson *et al.* [11] as part of a macrophage-enriched metabolic network in subcutaneous adipose tissue and associated with obesity-related traits.

| **HGNC symbol** | **MetS DE pvalue** | **BMI DE pvalue** | **ABD-GLU DE pvalue** | **MM** | **MM pvalue** | **Module** |
| --- | --- | --- | --- | --- | --- | --- |
| *LPL* | 3.9E-04 | 3.1E-03 | 0.16 | 0.82 | 3.0E-14 | brown |
| *ALOX5AP* | 2.0E-01 | 1.9E-03 | 0.32 | -0.73 | 4.2E-10 | blue |
| *C3AR1* | 5.3E-05 | 6.7E-07 | 2.9E-03 | 0.88 | 8.2E-19 | cyan |
| *TGFBR2* | 1.4E-01 | 0.14 | 0.01 | 0.62 | 6.6E-07 | darkturquoise |
| *ZFP90* | 1.1E-01 | 0.31 | 0.41 | 0.58 | 3.7E-07 | darkred |
| *HSD11B1* | 1.7E-04 | 1.0E-05 | 0.07 | 0.82 | 3.3E-10 | cyan |
| *CD68* | 1.3E-03 | 1.7E-04 | 0.33 | 0.77 | 9.8E-12 | cyan |
| *EMR1* | NA | NA | NA | NA | NA | NA |

MM = module membership; DE = differentially expressed
