# Supplementary material for: Coexpression Network Analysis in Abdominal and Gluteal Adipose Tissue Reveals Regulatory Genetic Loci for Metabolic Syndrome and Related Phenotypes
Source: PLoS Genet. 2012 Feb 23;8(2):e1002505. doi: 10.1371/journal.pgen.1002505 (PMC3285582; doi:10.1371/journal.pgen.1002505)
Supplement: Table S4 — Evaluation of GWA SNPs previously associated with MetS. (DOC) [file pgen.1002505.s011.doc]

**Table S4** Evaluation of GWA SNPs previously associated with MetS.

|  |  |  |  |  |  |  |  | **ABD** | | | | **GLU** | | | |
| --- | --- | --- | --- | --- | --- | --- | --- | --- | --- | --- | --- | --- | --- | --- | --- |
| **HGNC** | **SNP (GWA)** | **Trait** | **Chr** | **Position** | **SNP tested** | **r^2^** | **ABD-GLU DE pvalue** | **eQTL pvalue** | **MetS DE pvalue** | **MM pvalue** | **Module** | **eQTL pvalue** | **MetS DE pvalue** | **MM pvalue** | **Module** |
| *CAD* | rs1919128 | WC-TG | 2 | 27655263 | rs13030973 | 0.88 | 0.15 | 0.72 | 0.86 | 2.4E-05 | green | 0.12 | 0.72 | 2.6E-06 | red |
| *GTF3C2* | rs1919128 | WC-TG | 2 | 27655263 | rs13030973 | 0.88 | 0.27 | 0.66 | 0.62 | 1.7E-04 | green | 0.87 | 0.08 | 0.01 | brown |
| *MPV17* | rs1919128 | WC-TG | 2 | 27655263 | rs13030973 | 0.88 | 0.07 | 0.52 | 0.17 | 2.3E-06 | turquoise | 0.14 | 0.49 | 6.2E-09 | lightyellow |
| *PPM1G* | rs1919128 | WC-TG | 2 | 27655263 | rs13030973 | 0.88 | 0.20 | 0.22 | 0.15 | 9.8E-04 | brown | 0.9 | 0.78 | 5.6E-10 | darkturquoise |
| *SLC5A6* | rs1919128 | WC-TG | 2 | 27655263 | rs13030973 | 0.88 | 0.39 | 0.09 | 0.01 | 1.2E-06 | blue | 0.12 | 0.71 | 5.6E-14 | darkgrey |
| *EIF2B4* | rs1919128 | WC-TG | 2 | 27655263 | rs13030973 | 0.88 | 0.23 | 0.54 | 0.98 | 0.02 | blue | 0.18 | 0.3 | 5.7E-06 | red |
| *MRPL33* | rs1919128 | WC-TG | 2 | 27655263 | rs13030973 | 0.88 | 0.65 | 0.71 | 0.09 | 4.2E-08 | blue | 0.43 | 0.48 | 1.2E-06 | lightyellow |
| *SNX17* | rs1919128 | WC-TG | 2 | 27655263 | rs13030973 | 0.88 | 0.06 | 0.59 | 0.29 | 1.6E-04 | lightyellow | 0.43 | 0.25 | 9.9E-05 | turquoise |
| *SUPT7L* | rs1919128 | WC-TG | 2 | 27655263 | rs13030973 | 0.88 | 0.99 | 0.92 | 0.52 | 1.6E-03 | brown | 0.77 | 0.15 | 2.9E-04 | darkgrey |
| *GPN1* | rs1919128 | WC-TG | 2 | 27655263 | rs13030973 | 0.88 | 0.08 | 0.85 | 0.13 | 0.02 | brown | 0.42 | 0.49 | 1.4E-04 | magenta |
| *SLC4A1AP* | rs1919128 | WC-TG | 2 | 27655263 | rs13030973 | 0.88 | 0.77 | 0.62 | 0.02 | 2.4E-03 | brown | 0.68 | 0.87 | 2.6E-04 | orange |
| *NRBP1* | rs1919128 | WC-TG | 2 | 27655263 | rs13030973 | 0.88 | 0.23 | 0.34 | 0.14 | 0.28 | black | 0.99 | 0.67 | 1.6E-12 | greenyellow |
| *FNDC4* | rs1919128 | WC-TG | 2 | 27655263 | rs13030973 | 0.88 | 0.50 | 0.89 | 0.02 | 1.4E-06 | cyan | 0.43 | 2.8E-04 | 6.9E-07 | darkgreen |
| *CCDC121* | rs1919128 | WC-TG | 2 | 27655263 | rs13030973 | 0.88 | 0.95 | 0.1 | 0.06 | 0.01 | black | 0.19 | 0.2 | 5.1E-03 | brown |
| *ZNF512* | rs1919128 | WC-TG | 2 | 27655263 | rs13030973 | 0.88 | 0.38 | 0.23 | 0.8 | 1.7E-09 | magenta | 0.7 | 0.86 | 1.8E-07 | red |
| *ZNF513* | rs1919128 | WC-TG | 2 | 27655263 | rs13030973 | 0.88 | 0.70 | 0.68 | 0.47 | 2.6E-04 | grey60 | 0.79 | 0.63 | 2.2E-03 | greenyellow |
| *ATP6V1B2* | rs2197089 | TG-GLUC | 8 | 19870653 | rs894210 | 0.87 | 0.85 | 0.99 | 0.33 | 7.0E-12 | turquoise | 0.17 | 0.03 | 7.8E-13 | brown |
| *LPL* | rs301 | HDL-WC | 8 | 19861214 | rs4523270 | 0.77 | 0.16 | 0.02 | 3.9E-04 | 3.0E-14 | brown | 0.37 | 1.2E-03 | 2.3E-15 | turquoise |
| *LPL* | rs13702 | HDL-TG | 8 | 19868772 | rs4523270 | 0.7 | 0.16 | 0.02 | 3.9E-04 | 3.0E-14 | brown | 0.37 | 1.2E-03 | 2.3E-15 | turquoise |
| *LPL* | rs15285 | TG-BP | 8 | 19868947 | rs4523270 | 0.7 | 0.16 | 0.02 | 3.9E-04 | 3.0E-14 | brown | 0.37 | 1.2E-03 | 2.3E-15 | turquoise |
| *LPL* | rs2197089 | TG-GLUC | 8 | 19870653 | rs894210 | 0.87 | 0.16 | 0.2 | 3.9E-04 | 3.0E-14 | brown | 0.17 | 1.2E-03 | 2.3E-15 | turquoise |
| *INTS10* | rs301 | HDL-WC | 8 | 19861214 | rs4523270 | 0.77 | 0.20 | 0.34 | 0.46 | 5.8E-05 | magenta | 0.99 | 0.18 | 3.5E-03 | magenta |
| *INTS10* | rs301 | HDL-WC | 8 | 19861214 | rs2083637 | 0.88 | 0.20 | 0.38 | 0.46 | 5.8E-05 | magenta | 0.73 | 0.18 | 3.5E-03 | magenta |
| *INTS10* | rs13702 | HDL-TG | 8 | 19868772 | rs4523270 | 0.7 | 0.20 | 0.34 | 0.46 | 5.8E-05 | magenta | 0.99 | 0.18 | 3.5E-03 | magenta |
| *INTS10* | rs13702 | HDL-TG | 8 | 19868772 | rs2083637 | 0.81 | 0.20 | 0.38 | 0.46 | 5.8E-05 | magenta | 0.73 | 0.18 | 3.5E-03 | magenta |
| *INTS10* | rs15285 | TG-BP | 8 | 19868947 | rs4523270 | 0.7 | 0.20 | 0.34 | 0.46 | 5.8E-05 | magenta | 0.99 | 0.18 | 3.5E-03 | magenta |
| *INTS10* | rs15285 | TG-BP | 8 | 19868947 | rs2083637 | 0.81 | 0.20 | 0.38 | 0.46 | 5.8E-05 | magenta | 0.73 | 0.18 | 3.5E-03 | magenta |
| *INTS10* | rs2197089 | TG-GLUC | 8 | 19870653 | rs894210 | 0.87 | 0.20 | 0.54 | 0.46 | 5.8E-05 | magenta | 0.84 | 0.18 | 3.5E-03 | magenta |
| *FAT3* | rs1387153 | BP-GLUC | 11 | 92313476 | rs1387153 | 1 | 0.58 | 0.94 | 2.7E-04 | 4.0E-11 | brown | 0.08 | 3.1E-05 | 4.4E-10 | turquoise |
| *FAT3* | rs1387153 | HDL-GLUC | 11 | 92313476 | rs1387153 | 1 | 0.58 | 0.94 | 2.7E-04 | 4.0E-11 | brown | 0.08 | 3.1E-05 | 4.4E-10 | turquoise |
| *BBS2* | rs3764261 | BP-HDL | 16 | 55550825 | rs3764261 | 1 | 0.46 | 0.02 | 0.55 | 2.9E-05 | magenta | 0.45 | 0.87 | 2.6E-03 | darkgrey |
| *CETP* | rs3764261 | BP-HDL | 16 | 55550825 | rs3764261 | 1 | 0.03 | 0.25 | 0.06 | 8.5E-04 | cyan | 0.01 | 0.18 | 6.6E-06 | darkorange |
| *MT1F* | rs3764261 | BP-HDL | 16 | 55550825 | rs3764261 | 1 | 0.47 | 0.95 | 0.43 | 2.0E-04 | cyan | 0.77 | 0.06 | 3.6E-06 | darkred |
| *MT1M* | rs3764261 | BP-HDL | 16 | 55550825 | rs3764261 | 1 | 0.53 | 1 | 0.29 | 9.7E-06 | cyan | 0.53 | 0.05 | 1.7E-05 | darkred |
| *MT1X* | rs3764261 | BP-HDL | 16 | 55550825 | rs3764261 | 1 | 0.75 | 0.04 | 0.11 | 1.4E-03 | turquoise | 0.47 | 0.05 | 1.1E-03 | pink |
| *MT2A* | rs3764261 | BP-HDL | 16 | 55550825 | rs3764261 | 1 | 0.45 | 0.88 | 0.2 | 1.8E-07 | cyan | 0.61 | 1.6E-03 | 4.5E-12 | darkred |
| *CX3CL1* | rs3764261 | BP-HDL | 16 | 55550825 | rs3764261 | 1 | 0.01 | 0.58 | 0.92 | 3.1E-09 | salmon | 0.55 | 0.8 | 9.2E-11 | lightcyan |
| *HERPUD1* | rs3764261 | BP-HDL | 16 | 55550825 | rs3764261 | 1 | 0.11 | 4.9E-06 | 0.37 | 6.1E-04 | midnightblue | 6.5E-05 | 0.48 | 6.0E-04 | greenyellow |
| *ARL2BP* | rs3764261 | BP-HDL | 16 | 55550825 | rs3764261 | 1 | 0.10 | 0.98 | 1.9E-03 | 5.8E-07 | pink | 0.92 | 0.14 | 2.7E-12 | lightgreen |
| *OGFOD1* | rs3764261 | BP-HDL | 16 | 55550825 | rs3764261 | 1 | 4.2E-03 | 0.34 | 0.33 | 3.6E-06 | darkgrey | 0.54 | 0.01 | 3.8E-12 | cyan |
| *COQ9* | rs3764261 | BP-HDL | 16 | 55550825 | rs3764261 | 1 | 1.1E-03 | 0.98 | 5.7E-03 | 1.8E-15 | midnightblue | 0.12 | 0.02 | 1.9E-22 | turquoise |
| *FAM192A* | rs3764261 | BP-HDL | 16 | 55550825 | rs3764261 | 1 | 0.28 | 0.32 | 0.13 | 7.2E-04 | purple | 0.07 | 5.2E-03 | 0.07 | magenta |
| *NLRC5* | rs3764261 | BP-HDL | 16 | 55550825 | rs3764261 | 1 | 0.07 | 0.02 | 0.12 | 3.5E-11 | turquoise | 0.85 | 0.36 | 1.4E-10 | white |
| *MT4* | rs3764261 | BP-HDL | 16 | 55550825 | rs3764261 | 1 | 0.34 | 0.41 | 0.96 | 1.6E-04 | darkgreen | 0.89 | 0.68 | 3.0E-04 | green |
| *RSPRY1* | rs3764261 | BP-HDL | 16 | 55550825 | rs3764261 | 1 | 0.05 | 0.41 | 0.82 | 3.4E-04 | lightgreen | 0.35 | 0.09 | 1.1E-10 | red |
| *APOC1* | rs439401 | HDL-TG | 19 | 50106291 | rs439401 | 1 | 0.01 | 0.07 | 0.03 | 6.0E-09 | cyan | 0.27 | 0.09 | 3.3E-13 | black |
| *APOE* | rs439401 | HDL-TG | 19 | 50106291 | rs439401 | 1 | 0.88 | 8.5E-04 | 0.07 | 0.19 | black | 0.04 | 4.3E-07 | 3.3E-04 | darkred |
| *CLPTM1* | rs439401 | HDL-TG | 19 | 50106291 | rs439401 | 1 | 0.59 | 0.06 | 0.24 | 2.5E-13 | lightgreen | 0.85 | 0.07 | 3.7E-12 | grey60 |
| *ERCC1* | rs439401 | HDL-TG | 19 | 50106291 | rs439401 | 1 | 0.66 | 0.33 | 0.25 | 2.5E-04 | green | 0.3 | 0.43 | 3.5E-04 | blue |
| *ZNF180* | rs439401 | HDL-TG | 19 | 50106291 | rs439401 | 1 | 0.05 | 0.67 | 0.67 | 0.07 | blue | 0.77 | 0.32 | 0.01 | turquoise |
| *CLASRP* | rs439401 | HDL-TG | 19 | 50106291 | rs439401 | 1 | 0.24 | 0.27 | 0.68 | 0.06 | pink | 0.87 | 0.86 | 2.0E-10 | grey60 |
| *MARK4* | rs439401 | HDL-TG | 19 | 50106291 | rs439401 | 1 | 0.35 | 5.2E-03 | 0.48 | 1.0E-14 | green | 0.67 | 0.32 | 3.6E-07 | turquoise |
| *TRAPPC6A* | rs439401 | HDL-TG | 19 | 50106291 | rs439401 | 1 | 0.78 | 0.13 | 0.72 | 0.23 | blue | 0.17 | 0.02 | 1.3E-06 | magenta |

WC = waist circumference; GLUC = fasting glucose; BP= blood pressure; MM = module membership; DE = differentially expressed
