# Supplementary material for: Coexpression Network Analysis in Abdominal and Gluteal Adipose Tissue Reveals Regulatory Genetic Loci for Metabolic Syndrome and Related Phenotypes
Source: PLoS Genet. 2012 Feb 23;8(2):e1002505. doi: 10.1371/journal.pgen.1002505 (PMC3285582; doi:10.1371/journal.pgen.1002505)
Supplement: Table S5 — Hubgenes (genes with highest rank of module membership) in the modules strongest associated with MetS in the ABD and GLU single-tissue networks. (DOC) [file pgen.1002505.s012.doc]

**Table S5** Hubgenes (genes with highest rank of module membership) in the modules strongest associated with MetS in the ABD and GLU single-tissue networks.

| **Tissue** | **HGNC** | **Gene ID** | **MetS DE pvalue** | **MM** | **MM pvalue** | **Module** | **Consensus module** |
| --- | --- | --- | --- | --- | --- | --- | --- |
| ABD | *MOSC2* | 54996 | 1.0E-04 | 0.93 | 8.9E-24 | brown | turquoise |
| ABD | *CDKN2C* | 1031 | 2.5E-06 | 0.93 | 1.3E-23 | brown | turquoise |
| ABD | *MOSC1* | 64757 | 2.7E-05 | 0.92 | 3.9E-23 | brown | turquoise |
| ABD | *ACO2* | 50 | 2.1E-04 | 0.91 | 7.9E-22 | brown | turquoise |
| ABD | *ATP5B* | 506 | 3.8E-05 | 0.90 | 1.3E-20 | brown | turquoise |
| ABD | *LONP2* | 83752 | 4.5E-04 | 0.89 | 7.7E-20 | brown | turquoise |
| ABD | *UQCRC2* | 7385 | 2.4E-06 | 0.89 | 1.0E-19 | brown | turquoise |
| ABD | *ECHS1* | 1892 | 2.4E-04 | 0.89 | 1.8E-19 | brown | turquoise |
| ABD | *SUCLG1* | 8802 | 2.8E-05 | 0.89 | 2.1E-19 | brown | turquoise |
| ABD | *LDHD* | 197257 | 8.1E-06 | 0.89 | 4.7E-19 | brown | turquoise |
| ABD | *ALDH1A3* | 220 | 2.7E-06 | 0.92 | 5.6E-23 | cyan | yellow |
| ABD | *NPC2* | 10577 | 3.8E-04 | 0.91 | 1.5E-21 | cyan | yellow |
| ABD | *CD163* | 9332 | 1.1E-04 | 0.90 | 3.0E-20 | cyan | yellow |
| ABD | *MS4A4A* | 51338 | 2.1E-04 | 0.89 | 8.7E-20 | cyan | yellow |
| ABD | *GPNMB* | 10457 | 1.5E-04 | 0.89 | 1.5E-19 | cyan | yellow |
| ABD | *BCAT1* | 586 | 2.6E-04 | 0.89 | 3.0E-19 | cyan | yellow |
| ABD | *C3AR1* | 719 | 5.3E-05 | 0.88 | 8.2E-19 | cyan | yellow |
| ABD | *FOLR2* | 2350 | 5.1E-04 | 0.88 | 1.4E-18 | cyan | yellow |
| ABD | *C1QC* | 714 | 3.6E-04 | 0.88 | 3.0E-18 | cyan | yellow |
| ABD | *C1QB* | 713 | 2.3E-04 | 0.87 | 8.8E-18 | cyan | yellow |
| GLU | *GLUL* | 2752 | 3.8E-07 | -0.89 | 1.1E-22 | darkgreen | yellow |
| GLU | *PHLDA2* | 7262 | 3.9E-08 | 0.84 | 9.5E-19 | darkgreen | yellow |
| GLU | *ITGB5* | 3693 | 1.0E-10 | 0.84 | 1.1E-18 | darkgreen | yellow |
| GLU | *CD248* | 57124 | 3.0E-06 | 0.84 | 3.6E-18 | darkgreen | yellow |
| GLU | *ABCC1* | 4363 | 2.7E-07 | 0.83 | 1.3E-17 | darkgreen | yellow |
| GLU | *RPN2* | 6185 | 6.6E-08 | 0.82 | 1.0E-16 | darkgreen | purple |
| GLU | *GLB1* | 2720 | 3.8E-05 | 0.81 | 2.6E-16 | darkgreen | purple |
| GLU | *TXNRD1* | 7296 | 6.3E-05 | 0.81 | 2.8E-16 | darkgreen | yellow |
| GLU | *SYNC* | 81493 | 6.2E-07 | 0.81 | 3.4E-16 | darkgreen | purple |
| GLU | *HTRA1* | 5654 | 3.6E-05 | 0.81 | 4.0E-16 | darkgreen | yellow |

DE = differentially expressed; MM=module membership
