# Supplementary material for: Coexpression Network Analysis in Abdominal and Gluteal Adipose Tissue Reveals Regulatory Genetic Loci for Metabolic Syndrome and Related Phenotypes
Source: PLoS Genet. 2012 Feb 23;8(2):e1002505. doi: 10.1371/journal.pgen.1002505 (PMC3285582; doi:10.1371/journal.pgen.1002505)
Supplement: Table S6 — Modules for which eigengenes were significantly correlated (FDR p<0.01) with MetS in ABD (N = 6) and GLU (N = 6). FDR corrected pvalues for the associations with MetS and six quantitative metabolic traits are shown. (DOC) [file pgen.1002505.s013.doc]

**Table S6 Modules for which eigengenes were significantly correlated (FDR p<0.01) with MetS in ABD (N =6) and GLU (N=6). FDR corrected pvalues for the associations with MetS and six quantitative metabolic traits are shown.**

| **Module** | **Genes (N)** | **No. ABD-GLU DE genes (%)** | **MetS** |  | **Waist** |  | **HDL** |  | **TG** |  | **Diastbp** |  | **Systbp** |  | **Glucose** | |
| --- | --- | --- | --- | --- | --- | --- | --- | --- | --- | --- | --- | --- | --- | --- | --- | --- |
|  |  |  | **ABD** | **GLU** | **ABD** | **GLU** | **ABD** | **GLU** | **ABD** | **GLU** | **ABD** | **GLU** | **ABD** | **GLU** | **ABD** | **GLU** |
| yellow | 620 | 94 (15%) | 1.4E-05 | 4.6E-06 | 8.7E-11 | 9.4E-10 | 1.3E-09 | 5.8E-08 | 7.2E-04 | 2.3E-05 | 2.1E-04 | 3.6E-05 | 7.8E-03 | 1.5E-04 | 0.04 | 0.22 |
| turquoise | 2642 | 177 (7%) | 1.8E-04 | 4.0E-03 | 1.9E-06 | 2.9E-05 | 1.9E-06 | 5.3E-06 | 0.07 | 8.2E-04 | 5.8E-03 | 5.7E-03 | 0.08 | 8.5E-03 | 0.19 | 0.65 |
| darkgreen | 92 | 9 (10%) | 6.0E-04 | 4.0E-03 | 0.01 | 0.02 | 0.02 | 8.3E-03 | 0.40 | 8.7E-03 | 9.5E-04 | 0.11 | 0.03 | 0.06 | 0.40 | 0.56 |
| red | 569 | 95 (17%) | 1.2E-03 | 2.3E-03 | 7.8E-07 | 5.4E-06 | 2.5E-03 | 7.2E-03 | 0.07 | 0.05 | 2.1E-04 | 5.7E-03 | 2.0E-03 | 2.0E-03 | 0.10 | 0.22 |
| darkred | 97 | 11 (11%) | 3.9E-03 | 0.02 | 1.8E-03 | 2.2E-03 | 6.2E-05 | 8.0E-05 | 2.6E-03 | 2.1E-03 | 0.09 | 5.7E-03 | 0.35 | 0.03 | 0.19 | 0.35 |
| royalblue | 98 | 9 (9%) | 8.7E-03 | 4.6E-06 | 6.5E-03 | 6.2E-06 | 2.5E-03 | 1.5E-05 | 0.04 | 1.3E-05 | 6.6E-03 | 4.5E-04 | 0.07 | 4.1E-04 | 0.48 | 0.38 |
| purple | 224 | 63 (28%) | 0.03 | 8.5E-04 | 2.5E-06 | 1.0E-05 | 0.02 | 0.04 | 1.3E-03 | 0.03 | 9.5E-04 | 5.7E-03 | 2.6E-04 | 0.01 | 0.04 | 0.25 |

DE: differentially expressed between adipose fat depots
