# Supplementary material for: Coexpression Network Analysis in Abdominal and Gluteal Adipose Tissue Reveals Regulatory Genetic Loci for Metabolic Syndrome and Related Phenotypes
Source: PLoS Genet. 2012 Feb 23;8(2):e1002505. doi: 10.1371/journal.pgen.1002505 (PMC3285582; doi:10.1371/journal.pgen.1002505)
Supplement: Table S7 — Enrichment of Biological Processes GO terms in yellow consensus modules. (DOC) [file pgen.1002505.s014.doc]

**Table S7** Enrichment of Biological Processes GO terms in yellow consensus modules.

| **Depot** | **N genes** | **Term** | **Count** | **%** | **P value^**^** | **FE^***^** | **FDR P** |
| --- | --- | --- | --- | --- | --- | --- | --- |
| ABD_GLU | 620 | GO:0009611~response to wounding | 55 | 8.9 | 2.7E-12 | 2.9 | 1.8E-09 |
| ABD_GLU | 620 | GO:0006952~defense response | 57 | 9.2 | 9.1E-11 | 2.6 | 3.1E-08 |
| ABD_GLU | 620 | GO:0006959~humoral immune response | 17 | 2.7 | 1.5E-08 | 6.0 | 3.3E-06 |
| ABD_GLU | 620 | GO:0010033~response to organic substance | 50 | 8.1 | 1.1E-05 | 1.9 | 1.4E-03 |
| ABD_GLU | 620 | GO:0006956~complement activation | 10 | 1.6 | 1.4E-05 | 6.7 | 1.5E-03 |
| ABD_GLU | 620 | GO:0031589~cell-substrate adhesion | 15 | 2.4 | 9.5E-06 | 4.3 | 1.6E-03 |
| ABD_GLU | 620 | GO:0042592~homeostatic process | 49 | 7.9 | 6.3E-05 | 1.8 | 3.0E-03 |
| ABD_GLU | 620 | GO:0050778~positive regulation of immune response | 17 | 2.7 | 6.1E-05 | 3.3 | 3.1E-03 |
| ABD_GLU | 620 | GO:0045087~innate immune response | 17 | 2.7 | 3.3E-05 | 3.4 | 3.2E-03 |
| ABD_GLU | 620 | GO:0032101~regulation of response to external stimulus | 18 | 2.9 | 5.4E-05 | 3.2 | 3.3E-03 |
| ABD_GLU | 620 | GO:0002684~positive regulation of immune system process | 23 | 3.7 | 4.4E-05 | 2.7 | 3.3E-03 |
| ABD_GLU | 620 | GO:0048583~regulation of response to stimulus | 35 | 5.6 | 6.1E-05 | 2.1 | 3.4E-03 |
| ABD_GLU | 620 | GO:0010324~membrane invagination | 22 | 3.5 | 4.1E-05 | 2.8 | 3.5E-03 |
| ABD_GLU | 620 | GO:0006897~endocytosis | 22 | 3.5 | 4.1E-05 | 2.8 | 3.5E-03 |
| ABD_GLU | 620 | GO:0002682~regulation of immune system process | 31 | 5.0 | 5.3E-05 | 2.2 | 3.5E-03 |
| ABD_GLU | 620 | GO:0002250~adaptive immune response | 12 | 1.9 | 8.6E-05 | 4.4 | 3.6E-03 |
| ABD_GLU | 620 | GO:0030198~extracellular matrix organization | 14 | 2.3 | 8.3E-05 | 3.8 | 3.7E-03 |
| ABD_GLU | 620 | GO:0048584~positive regulation of response to stimulus | 22 | 3.5 | 1.1E-04 | 2.6 | 4.5E-03 |
| ABD_GLU | 620 | GO:0051128~regulation of cellular component organization | 33 | 5.3 | 2.3E-04 | 2.0 | 8.6E-03 |
| ABD_GLU^*^ | 94 | GO:0009611~response to wounding | 14 | 14.9 | 8.0E-06 | 4.5 | 2.3E-03 |

^*^GO enrichment analysis on genes differentially expressed between adipose depots only; ^**^P value = Fisher Exact Test; ^***^FE = Fold Enrichment
