# Supplementary material for: Coexpression Network Analysis in Abdominal and Gluteal Adipose Tissue Reveals Regulatory Genetic Loci for Metabolic Syndrome and Related Phenotypes
Source: PLoS Genet. 2012 Feb 23;8(2):e1002505. doi: 10.1371/journal.pgen.1002505 (PMC3285582; doi:10.1371/journal.pgen.1002505)
Supplement: Table S8 — Hubgenes (genes with highest rank of module membership) in the yellow consensus module in both the ABD and GLU networks. (DOC) [file pgen.1002505.s015.doc]

**Table S8** Hubgenes (genes with highest rank of module membership) in the yellow consensus module in both the ABD and GLU networks.

| **HGNC** | **Gene ID** | **MetS DE pvalue** | **MM** | **MM pvalue** | **MetS DE pvalue** | **MM** | **MM pvalue** | **DE pvalue** |
| --- | --- | --- | --- | --- | --- | --- | --- | --- |
|  |  | **ABD** | **ABD** | **ABD** | **GLU** | **GLU** | **GLU** | **ABD vs GLU** |
| *LHFPL2* | 10184 | 3.0E-05 | 0.86 | 4.1E-17 | 1.0E-04 | 0.84 | 9.4E-19 | 1.8E-03 |
| *GPNMB* | 10457 | 1.5E-04 | 0.89 | 3.7E-19 | 0.01 | 0.85 | 8.7E-19 | 0.71 |
| *NPC2* | 10577 | 3.8E-04 | 0.91 | 1.5E-21 | 5.0E-03 | 0.86 | 3.2E-20 | 4.1E-03 |
| *C22orf9* | 23313 | 3.0E-05 | 0.90 | 1.3E-20 | 2.4E-05 | 0.92 | 2.5E-27 | 4.6E-03 |
| *ADAP2* | 55803 | 1.6E-05 | 0.88 | 1.7E-18 | 2.7E-06 | 0.84 | 3.0E-18 | 6.9E-04 |
| *BCAT1* | 586 | 2.6E-04 | 0.90 | 2.0E-20 | 2.8E-03 | 0.84 | 2.5E-18 | 0.08 |
| *GPR137B* | 7107 | 2.4E-05 | 0.89 | 1.4E-19 | 1.4E-05 | 0.87 | 1.7E-21 | 0.12 |
| *C1QC* | 714 | 3.6E-04 | 0.87 | 1.9E-17 | 1.5E-04 | 0.84 | 2.4E-18 | 4.0E-05 |
| *C3AR1* | 719 | 5.3E-05 | 0.90 | 1.4E-20 | 4.7E-05 | 0.89 | 9.5E-24 | 7.6E-04 |
| *CD163* | 9332 | 1.1E-04 | 0.92 | 2.8E-22 | 5.6E-04 | 0.89 | 6.4E-23 | 2.7E-05 |

DE = differentially expressed; MM=module membership
