# Supplementary material for: Coexpression Network Analysis in Abdominal and Gluteal Adipose Tissue Reveals Regulatory Genetic Loci for Metabolic Syndrome and Related Phenotypes
Source: PLoS Genet. 2012 Feb 23;8(2):e1002505. doi: 10.1371/journal.pgen.1002505 (PMC3285582; doi:10.1371/journal.pgen.1002505)
Supplement: Table S9 — Median familiality estimates, assessed in MolTWIN ABD and WB, of four groups of MolOBB MetS-associated probesets identified using the single-gene approach, compared with probesets not associated with MetS. (DOC) [file pgen.1002505.s016.doc]

**Table S9 Median familiality estimates, assessed in MolTWIN ABD and WB, of four groups of MolOBB MetS-associated probesets identified using the single-gene approach, compared with probesets not associated with MetS.**

| **Probeset Group** | **Disease status** | **N** | **Depot (MolOBB)** | **Depot (MolTWIN)** | **Median familiality** | **IQR^**^** | **P value***** | **Median heritability** | **IQR^**^** | **P value***** |
| --- | --- | --- | --- | --- | --- | --- | --- | --- | --- | --- |
| 1) MolOBB ABD | MetS | 626 | ABD | ABD | 0.34 | 0.26 | <1.0E-15 | 0.39 | 0.46 | <1.0E-15 |
|  | No MetS | 6161 | ABD | ABD | 0.20 | 0.24 |  | 0.18 | 0.43 |  |
|  | MetS | 626 | ABD | WB | 0.24 | 0.22 | 1.7E-07 | 3.9E-10 | 0.27 | 0.05 |
|  | No MetS | 6161 | ABD | WB | 0.21 | 0.21 |  | 1.6E-10 | 0.23 |  |
| 2) MolOBB ABD+GLU | MetS | 121 | ABD+GLU | ABD | 0.43 | 0.18 | <1.0E-15 | 0.54 | 0.48 | <1.0E-15 |
|  | No MetS | 6666 | ABD+GLU | ABD | 0.21 | 0.24 |  | 0.2 | 0.44 |  |
|  | MetS | 121 | ABD+GLU | WB | 0.31 | 0.14 | 4.7E-08 | 7.7E-10 | 0.39 | 0.008 |
|  | No MetS | 6666 | ABD+GLU | WB | 0.21 | 0.12 |  | 1.6E-10 | 0.23 |  |
| 3) MolOBB ABD+GLU DE* | MetS | 22 | ABD+GLU | ABD | 0.41 | 0.15 | 9.7E-09 | 0.49 | 0.57 | 2.8E-10 |
|  | No MetS | 6765 | ABD+GLU | ABD | 0.21 | 0.24 |  | 0.2 | 0.45 |  |
|  | MetS | 22 | ABD+GLU | WB | 0.38 | 0.17 | 2.7E-06 | 2.8E-10 | 0.57 | 8.0E-04 |
|  | No MetS | 6765 | ABD+GLU | WB | 0.22 | 0.21 |  | 1.6E-10 | 0.23 |  |
| 4) MolOBB GLU | MetS | 205 | GLU | ABD | 0.38 | 0.22 | <1.0E-15 | 0.47 | 0.53 | <1.0E-15 |
|  | No MetS | 6582 | GLU | ABD | 0.20 | 0.24 |  | 0.2 | 0.44 |  |
|  | MetS | 205 | GLU | WB | 0.28 | 0.22 | 7.3E-09 | 7.7E-10 | 0.29 | 0.01 |
|  | No MetS | 6582 | GLU | WB | 0.21 | 0.21 |  | 1.6E-10 | 0.23 |  |

^*^DE = differentially expressed between adipose fat depots;^**^IQR = Interquartile Range; **^***^**Pvalues were calculated with Wilcoxon Rank Sum Test
