# Supplementary material for: Coexpression Network Analysis in Abdominal and Gluteal Adipose Tissue Reveals Regulatory Genetic Loci for Metabolic Syndrome and Related Phenotypes
Source: PLoS Genet. 2012 Feb 23;8(2):e1002505. doi: 10.1371/journal.pgen.1002505 (PMC3285582; doi:10.1371/journal.pgen.1002505)
Supplement: Text S1 — Members of GIANT consortium and members of MolPAGE consortium. (DOC) [file pgen.1002505.s017.doc]

**Text S1**

**^§^members of GIANT consortium**

(In alphabetical order)

Albert Vernon Smith^1,2^, Aaron R. Folsom^3^, Aila Rissanen^4^, Aimo Ruokonen^5^, Aki S. Havulinna^6^, Alan F. Wright^7^, Alan L. James^8,9^, Alan R. Sanders^10^, Alan R. Shuldiner^11,12^, Albert Hofman^13,14^, Albert Vernon Smith^1,2^, Aldi T. Kraja^15^, Alex N. Parker^16^, Alexander Teumer^17^, Alexandra I.F. Blakemore^18^, Alice M. Arnold^19,20^, Alistair S. Hall^21^, Amanda J. Bennett^22^, Amy J. Swift^23^, Anders Hamsten^24^, Andre Scherag^25^, André Uitterlinden^13,14,26^, Andreas Ziegler^27^, Andres Metspalu^28,29,30^, Andrew A. Hicks^31^, Andrew C. Heath^32^, Andrew P. Morris^33^, Andrew R. Wood^34^, Andrew T. Hattersley^34^, Anette P. Gjesing^35^, Anke Hinney^36^, Anke Tönjes^37,38^, Anna L. Dixon^39^, Anna-Liisa Hartikainen^40^, Ann-Christine Syvanen^41^, Anne McCarthy^42^, Anne U. Jackson^43^, Anneli Pouta^40,44^, Annette Peters^45^, Anthony J. Balmforth^21^, Antti Jula^46^, Anuj Goel^33,47^,Åsa Johansson^48,49^, Ayellet V. Segrè^50,51,52^, Barbara Ludwig^53^, Barbara McKnight^19^, Ben Oostra^54^, Benjamin F. Voight^50,51,52^, Beverley Balkau^55,56^, Bo Isomaa^57,58^, Brenda W. Penninx^59,60,61^, Brian Thomson^62^, Bruce M. Psaty^63,64^, Camilla Sandholt^35^, Candace Guiducci^62^, Carl G.P. Platou^65,66^, Carla I. G. Vogel^36^, Carlos Iribarren^67,68^, Caroline Hayward^7^, Caroline S. Fox^69^, Cecile Lecoeur^70,71^, Cecilia M. Lindgren^22,33^, Charles C. White^72^, Chiara Lanzani^73^, Chih-Mei Chen^45^, Christian Gieger^45^, Christian Hengstenberg^74,75^, Christina Barlassina^76^, Christine Cavalcanti-Proença^70,71^, Christopher C. Holmes^77,78^, Christopher J. Groves^22^, Christopher J. O'Donnell^79,80^, Claes Ohlsson^81^, Claudia B. Volpato^31^, Claudia Lamina^45,82^, Cliona Molony^83^, Cornelia M. van Duijn^13,14^, Cristen J. Willer*^43^, Cristen J. Willer^43^, Dale R. Nyholt^84^, Daniel I. Chasman^85,86^, Daniel R. Witte^87^, Danielle M. Greenawalt^83^, David J. Hunter^88,89,90^, David Meyre^70,71^, David P. Strachan^91^, David S. Siscovick^92,93^, David Schlessinger^94^, Debbie A. Lawlor^95^, Devin Absher^96^, Devin M. Absher^96^, Diana Marek^97,98^, Dorret I. Boomsma^99^, Douglas F. Levinson^100^, Eco J.C. Geus^99^, Eero Kajantie^101,102^, Eleanor Wheeler^103^, Elisabeth Thiering^45^, Elisabeth Widen^104^, Elizabeth Jewell^43^, Elizabeth K. Speliotes^62,105^, Eric Boerwinkle^106^, Eric E. Schadt^107,108^, Erik Ingelsson^109^, Eva Fisher^110^, Fabio Busonero^111^, Fabio Macciardi^76,112^, Fernando Rivadeneira^13,14,26^, Francesco P. Cappuccio^113^, Francis S. Collins^23^, Frank B. Hu^88,89,90^, Fredrik Karpe^22,114^, Fredrik Wiklund^109^, G. Bragi Walters^115^, G. Mark Lathrop^116^, G.Bragi Walters^115^, Georg Homuth^17^, George Davey Smith^95^, George Nicholson^78^, Gérard Waeber^117^, Gert-Jan van Ommen^118,119^, Gianluca Usala^111^, Gonçalo R. Abecasis^43^, Gonneke Willemsen^99^, Grant W. Montgomery^120^, Gudmar Thorleifsson*^115^, Gudmar Thorleifsson^115^, Gudny Eiriksdottir^1^, Guillaume Lettre^121,122^, Guillaume Paré^123^, H.-Erich Wichmann^45,124,125^, Hana Lango Allen^34^, Harald Grallert^45^, Harry Campbell^126^, Heather M. Stringham^43^, Heike Biebermann^127^, Heikki V. Huikuri^128,128^, Heiko Krude^127^, Heiner Boeing^110^, Helene Alavere^28^, Henri Wallaschofski^129^, Henrik Grönberg^109^, Henry Völzke^130^, Heribert Schunkert^131^, Heyo K. Kroemer^132^, Hugh Watkins on behalf of Procardis Consortium^33,47^, I. Sadaf Farooqi^133^, Ian N.M. Day^95^, Ida Surakka^104,134^, Igor Rudan^126,135^, Inês Barroso^103,136^, Inga Prokopenko^22,33^, Ingrid B. Borecki^15,137^, Inke R. König^27^, Irene Pichler^31^, Iris M. Heid^45,138^, Ivana Kolcic^139^, Ivonne Jarick^140^, Jaakko Kaprio^104,141,142^, Jaakko Tuomilehto^143,144,145^, Jaana Laitinen^146^, Jacqueline C. Witteman^13,14^, Jacques S. Beckmann^97,147^, James F. Wilson^126^, James Nemesh^51^, Jan H. Smit^61^, Jean-Claude Tardif^121,122^, Jeanette Erdmann^131^, Jeffrey R. O'Connell^11^, Jennie Hui^9,148,149^, Jennifer R. Kulzer^150^, Jian Yang^151^, Jian'an Luan^152^, Jianfeng Xu^153^, Jianjun Liu^154^, Jianxin Shi^155,155^, Jing Hua Zhao^152^, Joachim Heinrich^45^, Joel N. Hirschhorn*^62,156,157^, Joel N. Hirschhorn^62,156,157^, Johan G. Eriksson^57,101,158,159,160^, Johanna Kuusisto^161^, Johannes Hebebrand^36^, Johannes Kettunen^104,134^, John Connell^162^, John F. Peden^33,47^, John P. Beilby^9,148,163^, John R. Thompson^164,165^, John R.B. Perry^34^, John-Olov Jansson^166^, Jonathan P. Tyrer^167^, Jonathan Stephens^168,169^, Jorma Viikari^170^, Joshua C. Randall^33^, Joshua W. Knowles^171^, Josine L. Min^172^, Jouke-Jan Hottenga^99^, Jouko Saramies^173^, Joyce B.J. van Meurs^13,14,26^, Jubao Duan^10^, Juha Sinisalo^174^, Ju-Hyun Park^155^, Julius S. Ngwa^72^, Jürgen Gräßler^175^, Kaisa Silander^104,134^, Karen Kapur^97,98^, Karen L. Mohlke^150^, Kari E. North^176,177^, Kari Stefansson^115,178^, Karl-Heinz Herzig^179,180,181^, Karol Estrada^13,14,26^, Katja K. Aben^182^, Kay-Tee Khaw^183^, Ken K. Ong^152^, Keri L. Monda^176^, Kevin B. Jacobs^184^, Kirsi H. Pietiläinen^4,141^, Kirsti Kvaløy^65^, Klaus Stark^185^, Krina T. Zondervan^186^, Kristian Hveem^65^, Kristian Midthjell^65^, L. Adrienne Cupples^72^, Lachlan Coin^187^, Lambertus A. Kiemeney^182,188,189^, Larry D. Atwood^190^, Laura J. Scott^43^, Lee M. Kaplan^86,105,191^, Leena Kinnunen^143^, Leena Peltonen^103,104,134,192,193^, Leif C. Groop^194^, Lenore J. Launer^195^, Liesbeth Vandenput^81^, Liming Liang^88,196^, Lina Zgaga^139^, Lindsay L. Waite^96^, Lori L. Bonnycastle^23^, Lu Qi^89,90^, Lyle J. Palmer^9,149^, M.Carola Zillikens^14,26^, Manjinder S. Sandhu^103,152,183^, Manuela Uda^111^, Mao Fu^11^, Marcus Dörr^197^, Mari Nelis^28,29,30^, Maria T. S. Alibrandi^73^, Mariano Dei^111^, Marika Kaakinen^181,198^, Mari-Liis Tammesoo^28^, Mario A. Morken^23^, Maris Teder-Laving^29,30^, Marja-Liisa Lokki^199^, Marjolein J. Peters^14,26^, Marjo-Riitta Jarvelin^44,181,187,198^, Mark I. McCarthy^22,33,114^, Mark J. Caulfield^200^, Markku J. Savolainen^201^, Markku Laakso^161^, Markku S. Nieminen^174,174^, Markus Perola^104,134^, Martin den Heijer^202,203^, Martin Ridderstråle^204^, Martin Wabitsch^205^, Mary F. Feitosa^15^, Massimo Mangino^206^, Matt J. Neville^22^, Matthew N. Cooper^149^, Mattias Lorentzon^81^, Maurizio F. Facheris^31,207^, Michael Boehnke^43^, Michael E. Goddard^208,209^, Michael F. Leitzmann^138^, Michael N. Weedon^34^, Michael Preuss^27^, Michael R. Erdos^23^, Michael Stumvoll^37,210^, Michel Marre^211,212^, Mika Kähönen^213^, Miriam F. Moffatt^214^, Morris J. Brown^215^, Nabila Bouatia-Naji^70,71^, Najaf Amin^13^, Nancy L. Heard-Costa^190^, Narisu Narisu^23^, Neelam Hassanali^22^, Neil R. Robertson^22,33^, Nele Friedrich^129^, Nelson B. Freimer^216^, Nicholas G. Martin^217^, Nicholas J. Timpson^95^, Nicholas J. Wareham^152^, Nicole L. Glazer^92,218^, Nicole Soranzo^103,206^, Nigel W. Rayner^22,33^, Niina Pellikka^104,134^, Nilanjan Chatterjee^155^, Nilesh J. Samani^164,219^, Olivier Lantieri^220^, Olle Melander^204^, Olli Raitakari^202^, Oluf Pedersen^35,221,222^, Ozren Polasek^139,223^, Paavo Zitting^224^, Pablo V. Gejman^10^, Pamela Fischer-Posovszky^205^, Panos Deloukas^103^, Paolo Manunta^73^, Patricia B. Munroe^200^, Paul Elliott^187,225^, Paul M. Ridker^85,86^, Pekka Jousilahti^6^, Per Hall^109^, Peter Almgren^194^, Peter E.H. Schwarz^226^, Peter Kovacs^227^, Peter Kraft^88,196^, Peter M. Visscher^151^, Peter P. Pramstaller^31,207,228^, Peter Rzehak^45,125^, Peter S. Chines^23^, Peter Vollenweider^117^, Philippe Froguel^18,70,71^, Philippe Goyette^121^, Qunyuan Zhang^15^, Reedik Mägi^33^, Reiner Biffar^229^, Richard B. Hayes^230^, Richard H. Myers^231^, Richard M. Watanabe^232,233^, Richard N. Bergman^232^, Robert C. Kaplan^234^, Robert Clarke^235^, Robert J. Weyant^43^, Robert N. Luben^183^, Robert W. Lawrence^149^, Roberto Elosua^236^, Rona J. Strawbridge^24^, Rosanda Mulic^135^, Ruth J.F. Loos^152^, Ryan Welch^43^, Sabine Schipf^129,130^, Sailaja Vedantam^62,156^, Samuli Ripatti^104,134^, Sarah H. Wild^126^, Sekar Kathiresan^50,51,79,237,238^, Seppo Koskinen^6^, Serena Sanna^111^, Shah Ebrahim^239,240^, Shamika Ketkar^15^, Shaun Purcell^50,193,241^, Shengxu Li^152^, Sonja I. Berndt^155^, Sophie van Wingerden^13^, Sophie Visvikis-Siest^242^, Soumya Raychaudhuri^51,243^, Stefan Gaget^70,71^, Stefan Gustafsson^109^, Stefan R. Bornstein^53^, Stefan Schreiber^244^, Stephan B. Felix^197^, Stephen J. Chanock^155^, Stephen O’Rahilly^133^, Steven A. McCarroll^50,51,52^, Susann Scherag^36^, Susanna Wiegand^127^, Suzanne Rafelt^219^, Sven Bergmann^97,98^, Talin Haritunians^245^, Tamara B. Harris^195^, Tanja Boes^25^, Tanya M. Teslovich^43^, Teresa Ferreira^33^, Terho Lehtimäki^246^, Themistocles L. Assimes^171^, Thomas A. Buchanan^232,247^, Thomas Illig^45^, Thomas Meitinger^248,249^, Thomas Quertermous^171^, Thomas Reinehr^250^, Thomas W. Winkler^138^, Thor Aspelund^1,2^, Tiinamaija Tuomi^57,251,252^, Timo T. Valle^143^, Timothy D. Spector^206^, Timothy M. Frayling^34^, Toby Johnson^97,98,200,253^, Tõnu Esko^28,29,30^, Torben Hansen^35,254^, Torben Jørgensen^255,256^, Tsegaselassie Workalemahu^89^, Tuomas O. Kilpeläinen^152^, Ulf Gyllensten^48^, Ulla Sovio^187^, Ulrich John^257^, Unnur Thorsteinsdottir^115,178^, Valgerdur Steinthorsdottir^115^, Veikko Salomaa^6^, Veronique Vitart^7^, Vilmundur Gudnason^1,2^, Vincent Vatin^70,71^, Vjekoslav Krzelj^135^, Volker Hoesel^258^, W.H. Linda Kao^259^, Wendy L. McArdle^260^, Willem H. Ouwehand^103,168,169^, Wilmar Igl^48^, Winfried Rief^261^, Yii-Der Ida Chen^245^, Yoav Ben-Shlomo^262^, Zoltán Kutalik^97,98^

Reference List

1. Icelandic Heart Association, Kopavogur, Iceland

2. University of Iceland, Reykjavik, Iceland

3. Division of Epidemiology and Community Health, School of Public Health, University of Minnesota, Minneapolis Minnesota 55454, USA

4. Obesity Research unit, Department of Psychiatry, Helsinki University Central Hospital, Helsinki, Finland

5. Department of Clinical Sciences/Clinical Chemistry, University of Oulu, 90014 Oulu, Finland

6. National Institute for Health and Welfare, Department of Chronic Disease Prevention, Chronic Disease Epidemiology and Prevention Unit, 00014, Helsinki, Finland

7. MRC Human Genetics Unit, Institute for Genetics and Molecular Medicine, Western General Hospital, Edinburgh, EH4 2XU, Scotland, UK

8. School of Medicine and Pharmacology, University of Western Australia, Perth, Western Australia 6009, Australia

9. Busselton Population Medical Research Foundation Inc., Sir Charles Gairdner Hospital, Nedlands, Western Australia 6009, Australia

10. Northshore University Healthsystem, Evanston, Ilinois 60201, USA

11. Department of Medicine, University of Maryland School of Medicine, Baltimore, Maryland 21201, USA

12. Geriatrics Research and Education Clinical Center, Baltimore Veterans Administration Medical Center, Baltimore, Maryland 21201, USA

13. Department of Epidemiology, Erasmus MC, Rotterdam, 3015GE, The Netherlands

14. Netherlands Genomics Initiative (NGI)-sponsored Netherlands Consortium for Healthy Aging (NCHA)

15. Department of Genetics, Washington University School of Medicine, St Louis, Missouri 63110, USA

16. Amgen, Cambridge, Massachusetts 02139, USA

17. Interfaculty Institute for Genetics and Functional Genomics, Ernst-Moritz-Arndt-University Greifswald, 17487 Greifswald, Germany

18. Department of Genomics of Common Disease, School of Public Health, Imperial College London, W12 0NN, London, UK

19. Departments of Biostatistics, University of Washington, Seattle, Washington 98195, USA

20. Collaborative Health Studies Coordinating Center, Seattle, Washington 98115, USA

21. Multidisciplinary Cardiovascular Research Centre (MCRC), Leeds Institute of Genetics, Health and Therapeutics (LIGHT), University of Leeds, Leeds LS2 9JT, UK

22. Oxford Centre for Diabetes, Endocrinology and Metabolism, University of Oxford, Oxford, OX3 7LJ, UK

23. National Human Genome Research Institute, National Institutes of Health, Bethesda, Maryland 20892, USA

24. Atherosclerosis Research Unit, Department of Medicine, Solna,Karolinska Institutet, Karolinska University Hospital, 171 76 Stockholm, Sweden

25. Institute for Medical Informatics, Biometry and Epidemiology, University of Duisburg-Essen, 45122 Essen, Germany

26. Department of Internal Medicine, Erasmus MC, Rotterdam, 3015GE, The Netherlands

27. Institut für Medizinische Biometrie und Statistik, Universität zu Lübeck, Universitätsklinikum Schleswig-Holstein, Campus Lübeck, 23562 Lübeck, Germany

28. Estonian Genome Center, University of Tartu, Tartu 50410, Estonia

29. Estonian Biocenter, Tartu 51010, Estonia

30. Institute of Molecular and Cell Biology, University of Tartu, Tartu 51010, Estonia

31. Institute of Genetic Medicine, European Academy Bozen/Bolzano (EURAC), Bolzano/Bozen, 39100, Italy. Affiliated Institute of the University of Lübeck, Lübeck, Germany.

32. Department of Psychiatry and Midwest Alcoholism Research Center, Washington University School of Medicine, St Louis, Missouri 63108, USA

33. Wellcome Trust Centre for Human Genetics, University of Oxford, Oxford, OX3 7BN, UK

34. Genetics of Complex Traits, Peninsula College of Medicine and Dentistry, University of Exeter, Exeter, EX1 2LU, UK

35. Hagedorn Research Institute, 2820 Gentofte, Denmark

36. Department of Child and Adolescent Psychiatry, University of Duisburg-Essen, 45147 Essen, Germany

37. Department of Medicine, University of Leipzig, 04103 Leipzig, Germany

38. Coordination Centre for Clinical Trials, University of Leipzig, Härtelstr. 16-18, 04103 Leipzig, Germany

39. Department of Pharmacy and Pharmacology, University of Bath, Bath, BA1 1RL, UK

40. Department of Clinical Sciences/Obstetrics and Gynecology, University of Oulu, 90014 Oulu, Finland

41. Uppsala University / Dept. of Medical Sciences, Molecular Medicine, 751 85 Uppsala, Sweden

42. Division of Health, Research Board, An Bord Taighde Sláinte, Dublin, 2, Ireland

43. Department of Biostatistics, Center for Statistical Genetics, University of Michigan, Ann Arbor, Michigan 48109, USA

44. National Institute for Health and Welfare, 90101 Oulu, Finland

45. Institute of Epidemiology, Helmholtz Zentrum München - German Research Center for Environmental Health, 85764 Neuherberg, Germany

46. National Institute for Health and Welfare, Department of Chronic Disease Prevention, Population Studies Unit, 20720 Turku, Finland

47. Department of Cardiovascular Medicine, University of Oxford, Level 6 West Wing, John Radcliffe Hospital, Headley Way, Headington, Oxford, OX3 9DU

48. Department of Genetics and Pathology, Rudbeck Laboratory, University of Uppsala, SE-75185 Uppsala, Sweden

49. Department of Cancer Research and Molecular Medicine, Faculty of Medicine, Norwegian University of Science and Technology (NTNU), Trondheim, N-7489, Norway

50. Center for Human Genetic Research, Massachusetts General Hospital, Boston, Massachusetts 02114, USA.

51. Program in Medical and Population Genetics, Broad Institute of Harvard and Massachusetts Institute of Technology, Cambridge, Massachusetts 02142, USA

52. Department of Molecular Biology, Massachusetts General Hospital, Boston, Massachusetts 02114, USA

53. Department of Medicine III, University of Dresden, 01307 Dresden, Germany

54. Department of Clinical Genetics, Erasmus MC, Rotterdam, 3015GE, The Netherlands

55. INSERM CESP Centre for Research in Epidemiology and Public Health U1018, Epidemiology of diabetes, obesity and chronic kidney disease over the lifecourse, 94807 Villejuif, France

56. University Paris Sud 11, UMRS 1018, 94807 Villejuif, France

57. Folkhalsan Research Centre, 00250 Helsinki, Finland

58. Department of Social Services and Health Care, 68601 Jakobstad, Finland

59. Department of Psychiatry, Leiden University Medical Centre, 2300 RC Leiden, The Netherlands

60. Department of Psychiatry, University Medical Centre Groningen, 9713 GZ Groningen, The Netherlands

61. Department of Psychiatry/EMGO Institute, VU University Medical Center, 1081 BT Amsterdam, The Netherlands

62. Metabolism Initiative and Program in Medical and Population Genetics, Broad Institute, Cambridge, Massachusetts 02142, USA

63. Departments of Epidemiology, Medicine and Health Services, University of Washington, Seattle, Washington 98195, USA

64. Group Health Research Institute, Group Health, Seattle, Washington 98101, USA

65. HUNT Research Centre, Department of Public Health and General Practice, Norwegian University of Science and Technology, 7600 Levanger, Norway

66. Department of Medicine, Levanger Hospital, The Nord-Trøndelag Health Trust, 7600 Levanger, Norway

67. Division of Research, Kaiser Permanente Northern California, Oakland, California 94612, USA

68. Department of Epidemiology and Biostatistics, University of California, San Francisco, San Francisco, California 94107, USA

69. Division of Intramural Research, National Heart, Lung and Blood Institute, Framingham Heart Study, Framingham, Massachusetts 01702, USA

70. CNRS UMR8199-IBL-Institut Pasteur de Lille, F-59019 Lille, France

71. University Lille Nord de France, 59000 Lille, France

72. Department of Biostatistics, Boston University School of Public Health, Boston, Massachusetts 02118, USA

73. University Vita-Salute San Raffaele, Division of Nephrology and Dialysis, 20132 Milan, Italy

74. Klinik und Poliklinik für Innere Medizin II, Universität Regensburg, 93053 Regensburg, Germany

75. Regensburg University Medical Center, Innere Medizin II, 93053 Regensburg, Germany

76. University of Milan, Department of Medicine, Surgery and Dentistry, 20139 Milano, Italy

77. MRC Harwell, Harwell Science and Innovation Campus, Oxfordshire, OX11 0RD, UK

78. Department of Statistics, University of Oxford, Oxford OX1 3TG, UK

79. Framingham Heart Study of the National, Heart, Lung, and Blood Institute and Boston University, Framingham, Massachusetts 01702, USA

80. National, Lung, and Blood Institute, National Institutes of Health, Framingham, Massachusetts 01702, USA

81. Department of Internal Medicine, Institute of Medicine, Sahlgrenska Academy, University of Gothenburg, 413 45 Gothenburg, Sweden

82. Division of Genetic Epidemiology, Department of Medical Genetics, Molecular and Clinical Pharmacology, Innsbruck Medical University, 6020 Innsbruck, Austria

83. Merck Research Laboratories, Merck & Co., Inc., Boston, Massachusetts 02115, USA

84. Neurogenetics Laboratory, Queensland Institute of Medical Research, Queensland 4006, Australia

85. Division of Preventive Medicine, Brigham and Women's Hospital, Boston, Massachusetts 02215, USA

86. Harvard Medical School, Boston, Massachusetts 02115, USA

87. Steno Diabetes Center, 2820 Gentofte, Denmark

88. Department of Epidemiology, Harvard School of Public Health, Boston, Massachusetts 02115, USA

89. Department of Nutrition, Harvard School of Public Health, Boston, Massachusetts 02115, USA

90. Channing Laboratory, Department of Medicine, Brigham and Women's Hospital and Harvard Medical School, Boston, Massachusetts 02115, USA

91. Division of Community Health Sciences, St George's, University of London, London, SW17 0RE, UK

92. Cardiovascular Health Research Unit, University of Washington, Seattle, Washington 98101, USA

93. Departments of Medicine and Epidemiology, University of Washington, Seattle, Washington 98195, USA

94. Laboratory of Genetics, National Institute on Aging, Baltimore, Maryland 21224, USA

95. MRC Centre for Causal Analyses in Translational Epidemiology, Department of Social Medicine, Oakfield House, Bristol, BS8 2BN, UK

96. Hudson Alpha Institute for Biotechnology, Huntsville, Alabama 35806, USA

97. Department of Medical Genetics, University of Lausanne, 1005 Lausanne, Switzerland

98. Swiss Institute of Bioinformatics, 1015 Lausanne, Switzerland

99. Department of Biological Psychology, VU University Amsterdam, 1081 BT Amsterdam, The Netherlands

100. Stanford University School of Medicine, Stanford, California 93405, USA

101. National Institute for Health and Welfare, 00271 Helsinki, Finland

102. Hospital for Children and Adolescents, Helsinki University Central Hospital and University of Helsinki, 00029 HUS, Finland

103. Wellcome Trust Sanger Institute, Hinxton, Cambridge, CB10 1SA, UK

104. Institute for Molecular Medicine Finland (FIMM), University of Helsinki, 00014, Helsinki, Finland

105. Division of Gastroenterology, Massachusetts General Hospital, Boston, Massachusetts 02114, USA

106. Human Genetics Center and Institute of Molecular Medicine, University of Texas Health Science Center, Houston, Texas 77030, USA

107. Pacific Biosciences, Menlo Park, California 94025, USA

108. Sage Bionetworks, Seattle, Washington 98109, USA

109. Department of Medical Epidemiology and Biostatistics, Karolinska Institutet, 171 77 Stockholm, Sweden

110. Department of Epidemiology, German Institute of Human Nutrition Potsdam-Rehbruecke, 14558 Nuthetal, Germany

111. Istituto di Neurogenetica e Neurofarmacologia del CNR, Monserrato, 09042, Cagliari, Italy

112. Department of Psychiatry and Human Behavior, University of California, Irvine (UCI), Irvine, California 92617, USA

113. University of Warwick, Warwick Medical School, Coventry, CV2 2DX, UK

114. NIHR Oxford Biomedical Research Centre, Churchill Hospital, Oxford, OX3 7LJ, UK

115. deCODE Genetics, 101 Reykjavik, Iceland

116. Centre National de Genotypage, Evry, Paris 91057, France

117. Department of Internal Medicine, Centre Hospitalier Universitaire Vaudois (CHUV) University Hospital, 1011 Lausanne, Switzerland

118. Department of Human Genetics, Leiden University Medical Center, 2333 ZC Leiden, the Netherlands

119. Center of Medical Systems Biology, Leiden University Medical Center, 2333 ZC Leiden, the Netherlands

120. Molecular Epidemiology Laboratory, Queensland Institute of Medical Research, Queensland 4006, Australia

121. Montreal Heart Institute, Montreal, Quebec, H1T 1C8, Canada

122. Department of Medicine, Université de Montréal, Montreal, Quebec, H3T 1J4, Canada

123. Department of Pathology and Molecular Medicine, McMaster University, Hamilton, Ontario L8N3Z5, Canada

124. Klinikum Grosshadern, 81377 Munich, Germany

125. Ludwig-Maximilians-Universität, Institute of Medical Informatics, Biometry and Epidemiology, Chair of Epidemiology, 81377 Munich, Germany

126. Centre for Population Health Sciences, University of Edinburgh, Teviot Place, Edinburgh, EH8 9AG, Scotland

127. Institute of Experimental Paediatric Endocrinology, Charité Universitätsmedizin Berlin, 13353 Berlin, Germany

128. Department of Internal Medicine, University of Oulu, 90014 Oulu, Finland

129. Institut für Klinische Chemie und Laboratoriumsmedizin, Universität Greifswald, 17475 Greifswald, Germany

130. Institut für Community Medicine, 17489 Greifswald, Germany

131. Universität zu Lübeck, Medizinische Klinik II, 23562 Lübeck, Germany

132. Institut für Pharmakologie, Universität Greifswald, 17487 Greifswald, Germany

133. University of Cambridge Metabolic Research Laboratories, Institute of Metabolic Science, Addenbrooke's Hospital, Cambridge CB2 0QQ, UK

134. National Institute for Health and Welfare, Department of Chronic Disease Prevention, Unit of Public Health Genomics, 00014, Helsinki, Finland

135. Croatian Centre for Global Health, School of Medicine, University of Split, Split 21000, Croatia

136. University of Cambridge Metabolic Research Labs, Institute of Metabolic Science Addenbrooke's Hospital, CB2 OQQ, Cambridge, UK

137. Division of Biostatistics,Washington University School of Medicine,St.Louis, Missouri 63110, USA

138. Regensburg University Medical Center, Department of Epidemiology and Preventive Medicine, 93053 Regensburg, Germany

139. Andrija Stampar School of Public Health, Medical School, University of Zagreb, 10000 Zagreb, Croatia

140. Institute of Medical Biometry and Epidemiology, University of Marburg, 35037 Marburg, Germany

141. Finnish Twin Cohort Study, Department of Public Health, University of Helsinki,
00014, Helsinki, Finland

142. National Institute for Health and Welfare, Department of Mental Health and Substance Abuse Services, Unit for Child and Adolescent Mental Health, 00271 Helsinki, Finland

143. National Institute for Health and Welfare, Diabetes Prevention Unit, 00271 Helsinki, Finland

144. Hjelt Institute, Department of Public Health, University of Helsinki, 00014 Helsinki, Finland

145. South Ostrobothnia Central Hospital, 60220 Seinajoki, Finland

146. Finnish Institute of Occupational Health, 90220 Oulu, Finland

147. Service of Medical Genetics, Centre Hospitalier Universitaire Vaudois (CHUV) University Hospital, 1011 Lausanne, Switzerland

148. PathWest Laboratory of Western Australia, Department of Molecular Genetics, J Block, QEII Medical Centre, Nedlands, Western Australia 6009, Australia

149. Centre for Genetic Epidemiology and Biostatistics, University of Western Australia, Crawley, Western Australia 6009, Australia

150. Department of Genetics, University of North Carolina, Chapel Hill, North Carolina 27599, USA

151. Queensland Statistical Genetics Laboratory, Queensland Institute of Medical Research, Queensland 4006, Australia

152. MRC Epidemiology Unit, Institute of Metabolic Science, Addenbrooke's Hospital, Cambridge, CB2 0QQ, UK

153. Center for Human Genomics, Wake Forest University, Winston-Salem, North Carolina 27157, USA

154. Human Genetics, Genome Institute of Singapore, Singapore 138672, Singapore

155. Division of Cancer Epidemiology and Genetics, National Cancer Institute, National Institutes of Health, Department of Health and Human Services, Bethesda, Maryland 20892, USA

156. Divisions of Genetics and Endocrinology and Program in Genomics, Children's Hospital, Boston, Massachusetts 02115, USA

157. Department of Genetics, Harvard Medical School, Boston, Massachusetts 02115, USA

158. Department of General Practice and Primary health Care, University of Helsinki, Helsinki, Finland

159. Helsinki University Central Hospital, Unit of General Practice, 00280 Helsinki, Finland

160. Vasa Central Hospital, 65130 Vasa, Finland

161. Department of Medicine, University of Kuopio and Kuopio University Hospital, 70210 Kuopio, Finland

162. University of Dundee, Ninewells Hospital &Medical School, Dundee, DD1 9SY, UK

163. School of Pathology and Laboratory Medicine, University of Western Australia, Nedlands, Western Australia 6009,Australia

164. Leicester NIHR Biomedical Research Unit in Cardiovascular Disease, Glenfield Hospital, Leicester, LE3 9QP, UK

165. Department of Health Sciences, University of Leicester, University Road, Leicester, LE1 7RH, UK

166. Department of Physiology, Institute of Neuroscience and Physiology, Sahlgrenska Academy, University of Gothenburg, 405 30 Gothenburg, Sweden

167. Department of Oncology, University of Cambridge, Cambridge, CB1 8RN, UK

168. Department of Haematology, University of Cambridge, Cambridge CB2 0PT, UK

169. NHS Blood and Transplant, Cambridge Centre, Cambridge, CB2 0PT, UK

170. Department of Medicine, University of Turku and Turku University Hospital, 20520 Turku, Finland

171. Department of Medicine, Stanford University School of Medicine, Stanford, California 94305, USA

172. Human Genetics, Leiden University Medical Center, Leiden 2333, The Netherlands

173. South Karelia Central Hospital, 53130 Lappeenranta, Finland

174. Division of Cardiology, Cardiovascular Laboratory, Helsinki University Central Hospital, 00029 Helsinki, Finland

175. Department of Medicine III, Pathobiochemistry, University of Dresden, 01307 Dresden, Germany

176. Department of Epidemiology, School of Public Health, University of North Carolina at Chapel Hill, Chapel Hill, North Carolina 27514, USA

177. Carolina Center for Genome Sciences, School of Public Health, University of North Carolina Chapel Hill, Chapel Hill, North Carolina 27514, USA

178. Faculty of Medicine, University of Iceland, 101 Reykjavík, Iceland

179. Institute of Biomedicine, Department of Physiology, University of Oulu, 90014 Oulu, Finland

180. Department of Psychiatry, Kuopio University Hospital and University of Kuopio, 70210 Kuopio, Finland

181. Biocenter Oulu, University of Oulu, 90014 Oulu, Finland

182. Comprehensive Cancer Center East, 6501 BG Nijmegen, The Netherlands

183. Department of Public Health and Primary Care, Institute of Public Health, University of Cambridge, Cambridge CB2 2SR, UK

184. Core Genotyping Facility, SAIC-Frederick, Inc., NCI-Frederick, Frederick, Maryland 21702, USA

185. Regensburg University Medical Center, Clinic and Policlinic for Internal Medicine II, 93053 Regensburg, Germany

186. Genetic and Genomic Epidemiology Unit, Wellcome Trust Centre for Human Genetics, OX3 7BN, Oxford

187. Department of Epidemiology and Biostatistics, School of Public Health, Faculty of Medicine, Imperial College London, London, W2 1PG, UK

188. Department of Epidemiology, Biostatistics and HTA, Radboud University Nijmegen Medical Centre, 6500 HB Nijmegen, The Netherlands

189. Department of Urology, Radboud University Nijmegen Medical Centre, 6500 HB Nijmegen, The Netherlands

190. Department of Neurology, Boston University School of Medicine, Boston, Massachusetts 02118, USA

191. MGH Weight Center, Massachusetts General Hospital, Boston, Massachusetts 02114, USA

192. Department of Medical Genetics, University of Helsinki, 00014 Helsinki, Finland

193. The Broad Institute of Harvard and MIT, Cambridge, Massachusetts 02142, USA

194. Lund University Diabetes Centre, Department of Clinical Sciences, Lund University, 20502 Malmö, Sweden

195. Laboratory of Epidemiology, Demography, Biometry, National Institute on Aging, National Institutes of Health, Bethesda, Maryland 20892, USA

196. Department of Biostatistics, Harvard School of Public Health, Boston, Massachusetts 02115, USA

197. Department of Internal Medicine B, Ernst-Moritz-Arndt University, 17475 Greifswald, Germany

198. Institute of Health Sciences, University of Oulu, 90014 Oulu, Finland

199. Transplantation Laboratory, Haartman Institute, University of Helsinki, 00014, Helsinki, Finland

200. Clinical Pharmacology and Barts and The London Genome Centre, William Harvey Research Institute, Barts and The London School of Medicine and Dentistry, Queen Mary University of London, Charterhouse Square, London EC1M 6BQ, UK

201. Department of Clinical Sciences/Internal Medicine, University of Oulu, 90014 Oulu, Finland

202. Research Centre of Applied and Preventive Cardiovascular Medicine, University of Turku, 20520 Turku, Finland

203. Department of Endocrinology, Radboud University Nijmegen Medical Centre, 6500 HB Nijmegen, The Netherlands

204. Department of Clinical Sciences, Lund University, 20502 Malmö, Sweden

205. Pediatric Endocrinology, Diabetes and Obesity Unit, Department of Pediatrics and Adolescent Medicine, 89075 Ulm, Germany

206. Department of Twin Research and Genetic Epidemiology, King's College London, London, SE1 7EH, UK

207. Department of Neurology, General Central Hospital, Bolzano, Italy

208. University of Melbourne, Parkville 3010, Australia

209. Department of Primary Industries, Melbourne, Victoria 3001, Australia

210. LIFE Study Centre, University of Leipzig, Leipzig, Germany

211. Department of Endocrinology, Diabetology and Nutrition, Bichat-Claude Bernard University Hospital, Assistance Publique des Hôpitaux de Paris, F-75018 Paris, France

212. Cardiovascular Genetics Research Unit, Université Henri Poincaré-Nancy 1, 54000, Nancy, France

213. Department of Clinical Physiology, University of Tampere and Tampere University Hospital, 33520 Tampere, Finland;

214. National Heart and Lung Institute, Imperial College London, London SW3 6LY, UK

215. Clinical Pharmacology Unit, University of Cambridge, Addenbrooke's Hospital, Hills Road, Cambridge CB2 2QQ, UK

216. Center for Neurobehavioral Genetics, University of California, Los Angeles, California 90095, USA

217. Genetic Epidemiology Laboratory, Queensland Institute of Medical Research, Queensland 4006, Australia

218. Department of Medicine, University of Washington, Seattle, Washington 98101, USA

219. Department of Cardiovascular Sciences, University of Leicester, Glenfield Hospital, Leicester, LE3 9QP, UK

220. Institut inter-regional pour la sante (IRSA), F-37521 La Riche, France.

221. Institute of Biomedical Sciences, University of Copenhagen, 2200 Copenhagen, Denmark

222. Faculty of Health Science, University of Aarhus, 8000 Aarhus, Denmark

223. Gen-Info Ltd, 10000 Zagreb, Croatia

224. Department of Physiatrics, Lapland Central Hospital, 96101 Rovaniemi, Finland

225. MRC-HPA Centre for Environment and Health, London W2 1PG, UK

226. Department of Medicine III, Prevention and Care of Diabetes, University of Dresden, 01307 Dresden,Germany

227. Interdisciplinary Centre for Clinical Research, University of Leipzig, 04103 Leipzig, Germany

228. Department of Neurology, University of Lübeck, Lübeck, Germany.

229. Zentrum für Zahn-, Mund- und Kieferheilkunde, 17489 Greifswald, Germany

230. New York University Medical Center, New York, New York 10016, USA

231. Department of Neurology, Boston University School of Medicine, Boston, Massachusetts 02118, USA

232. Department of Physiology and Biophysics, Keck School of Medicine, University of Southern California, Los Angeles, California 90033, USA

233. Department of Preventive Medicine, Keck School of Medicine, University of Southern California, Los Angeles, California 90089, USA

234. Department of Epidemiology and Population Health, Albert Einstein College of Medicine, Bronx, New York 10461, USA

235. Clinical Trial Service Unit, Richard Doll Building, Old Road Campus, Roosevelt Drive, Oxford, OX3 7LF, UK

236. Cardiovascular Epidemiology and Genetics, Institut Municipal D'investigacio Medica and CIBER Epidemiología y Salud Pública, Barcelona, Spain

237. Cardiovascular Research Center and Cardiology Division, Massachusetts General Hospital, Boston, Massachusetts 02114, USA.

238. Department of Medicine, Harvard Medical School, Boston, Massachusetts 02115, USA

239. The London School of Hygiene and Tropical Medicine, London, WC1E 7HT, UK

240. South Asia Network for Chronic Disease

241. Department of Psychiatry, Harvard Medical School, Boston, Massachusetts 02115, USA

242. INSERM Cardiovascular Genetics team, CIC 9501, 54000 Nancy, France

243. Division of Rheumatology, Immunology and Allergy, Brigham and Women's Hospital, Harvard Medical School, Boston, Massachusetts 02115 USA

244. Christian-Albrechts-University, University Hospital Schleswig-Holstein, Institute for Clinical Molecular Biology and Department of Internal Medicine I, 24105 Kiel, Germany

245. Medical Genetics Institute, Cedars-Sinai Medical Center, Los Angeles, California 90048, USA

246. Department of Clinical Chemistry, University of Tampere and Tampere University Hospital, 33520 Tampere, Finland

247. Division of Endocrinology, Keck School of Medicine, University of Southern California, Los Angeles, California 90033, USA

248. Institute of Human Genetics, Klinikum rechts der Isar der Technischen Universität München, 81675 Munich, Germany

249. Institute of Human Genetics, Helmholtz Zentrum München - German Research Center for Environmental Health, 85764 Neuherberg, Germany

250. Institute for Paediatric Nutrition Medicine, Vestische Hospital for Children and Adolescents, University of Witten-Herdecke, 45711 Datteln, Germany

251. Department of Medicine, Helsinki University Central Hospital, 00290 Helsinki, Finland

252. Research Program of Molecular Medicine, University of Helsinki, 00014 Helsinki, Finland

253. Clinical Pharmacology, William Harvey Research Institute, Barts and The London School of Medicine and Dentistry, Queen Mary, University of London, London, UK

254. Faculty of Health Science, University of Southern Denmark, 5000 Odense, Denmark

255. Research Centre for Prevention and Health, Glostrup University Hospital, 2600 Glostrup, Denmark

256. Faculty of Health Science, University of Copenhagen, 2100 Copenhagen, Denmark

257. Institut für Epidemiologie und Sozialmedizin, Universität Greifswald, 17475 Greifswald, Germany

258. Technical University Munich, Chair of Biomathematics, Boltzmannstrasse 3, 85748 Garching

259. Department of Epidemiology and Medicine, Johns Hopkins Bloomberg School of Public Health, Baltimore, Maryland 21205, USA

260. Avon Longitudinal Study of Parents and Children (ALSPAC) Laboratory, Department of Social Medicine, University of Bristol, Bristol, BS8 2BN, UK

261. Clinical Psychology and Psychotherapy, University of Marburg, Gutenbergstrasse 18, 35032 Marburg, Germany

262. Department of Social Medicine, University of Bristol, Bristol, BS8 2PS, UK

**#MolPAGE consortium partners**

(In no particular order)

John Bell^1^, Mark McCarthy^1^, Lon Cardon^1^, Peter Donnelly^1^, Edwin Southern^2^, John Anson^2^, Mark Lathrop^3^, Ivo Gut^3^, Matthias Schuster^4^, Kurt Berlin^4^, Esper Boel^5^, Jan Fleckner^5^, Mathias Uhlén^6^, Thomas Bergman^7^, Vladimir Stich^8^, Alvis Brazma^9^, Ugis Sarkans^9^, Juris Viksna^10^, Jeremy Nicholson^11^, Luisa Bernardinelli^12^, Stephen Hoffmann^13^, Mats Inganas^13^, Tim Spector^14^, Dominique Langin^15^, Fredrik Ponten^16^ , Hanno Langen^17^, Stefan Evers^17^

1. University of Oxford, Oxford, UK

2. Oxford Gene Technology, Oxford, UK

3. Centre National de Génotypage, Evry, France

4. Epigenomics AG, Berlin, Germany

5. Novo Nordisk, Copenhagen, Denmark

6. Royal Institute of Technology, Stockholm, Sweden

7. Affibody AB, Bromma, Sweden

8. Charles University Prague, Czech Republic

9. European Bioinformatics Institute, Cambridge, UK

10. Institute of Mathematics and Computer Science, Riga, Latvia

11. Imperial College of Science, Technology and Medicine, London, UK

12. University of Pavia, Pavia, Italy

13. Gyros AB, Uppsala, Sweden

14. Guys and St Thomas Hospital NHS Trust, London, UK

15. Obesity Research Unit, INSERM, Toulouse, France

16. University of Uppsala, Uppsala, Sweden

17. Roche, Basel, Switzerland
