# Supplementary material for: Coexpression Network Analysis in Abdominal and Gluteal Adipose Tissue Reveals Regulatory Genetic Loci for Metabolic Syndrome and Related Phenotypes
Source: PLoS Genet. 2012 Feb 23;8(2):e1002505. doi: 10.1371/journal.pgen.1002505 (PMC3285582; doi:10.1371/journal.pgen.1002505)
Supplement: Text S2 — Variability of MetS-associated gene expression. (DOC) [file pgen.1002505.s018.doc]

**Text S2**

**Variability of MetS-associated gene expression**

In addition to familiality, the proportion of variation within individuals attributable to multiple visits (individual visit effect) was significantly higher in ABD (median = 0.22, IQR = 0.19) than in WB (median =0.14, IQR = 0.17, p =5.9*10^-26^) for the 626 ABD MetS-associated probesets (Figure 2A). In contrast, the proportion of variation contributed by twins visiting the hospital in pairs (common visit effect) was significantly lower in ABD (median = 0.09, IQR = 0.14) than in WB (median =0.19, IQR = 0.21, p =1.2*10^-32^). The same pattern was observed for the 205 GLU MetS-associated probesets (Figure 2B). Variability of the 20 module eigengenes from MolOBB ABD due to individual visit effects was greater in MolTWIN ABD (median = 0.29 ,IQR = 0.27) than in WB (median = 0.13, IQR = 0.06, p= 0.009). Variability due to common visit effects was greater in MolTWIN WB (median 0.34, IQR = 0.09) than in ABD (median = 0.15, IQR = 0.16, p=1.9*10^-6^). Overall, our findings showed increased patterns of familiality for MetS-associated probesets in ABD and WB. In addition, our results suggested that, within individuals, variation in expression across fat biopsies is greater than across WB samples, whilst the latter displayed a larger influence of variation in expression attributable to twins visiting the clinic in pairs to donate samples.
